# Microclimate monitoring in the Carcer Tullianum: temporal and spatial correlation and gradients evidenced by multivariate analysis; first campaign

**DOI:** 10.1186/1752-153X-6-S2-S11

**Published:** 2012-05-02

**Authors:** Giovanni Visco, Susanne Heidi Plattner, Patrizia Fortini, Serena Di Giovanni, Maria Pia Sammartino

**Affiliations:** 1 Rome University, La Sapienza, Math. Phy. Nat. Science Faculty, Piazzale A.Moro 5, 00185 Rome, Italy; 2 Cultural Heritage Superintendence of Rome City Council, Pz. Lovatelli 35, 00186 Rome, Italy

## Abstract

Too often microclimate studies in the field of cultural heritage are published without any or scarce information on sampling design, sensors (type, number, position) and instrument validation. Lacking of this fundamental information does not allow an open discussion in the scientific community. This work aims to be an invitation for a different approach.

Three main parameters (temperature, humidity, luminance) were monitored in a selected part of a complex construction by an inexpensive self-assembled system along some horizontal and vertical vectors. All data was then processed and analyse by chemometric methods. Some measurements of oxygen, carbon monoxide and dioxide and pressure were also performed.

Correlation of some indoor and outdoor data was shown for temperature and humidity. In case of outdoor changes the indoor environment reacted with a certain delay which is position-dependent and more evident for humidity data. The two observed rooms (Carcer and Tullianum) behave differently and the hypogean one is less influenced by the outdoor environment. Instrument validation before and after the campaign, allows to consider detected variations as significant.

The fundamental importance of Sampling Design and of instrument validation before and after the monitoring campaign was enhanced. The choice of two main and two minor vectors allowed detection of different behaviour for the two rooms, also permitting to detect for both rooms a trend towards a spontaneous microclimate necessary for a conservation project. In the next campaign we will focus on the choice of the best sampling frequency to use more sophisticated statistical methods.

## Background

Microclimate never can be univocally defined in space; should one consider the indoor part of a building or restrict it to a single room?

Obviously, correct spatial definition of a microclimate should also consider all surfaces (walls, statues, coins, etc.); their climatic conditions are indeed influenced by that of the surrounding environment, but depend also on the material.

Very often the definition is referred to a building or at least to a partially isolated area.

For a building a ‘mesoclimate’ can be defined, which is influenced by the building itself, its structure, geometry, shape and materials and by the surroundings.

For our purpose we define a macroclimate, characterised by locally measured meteor-climatic values, which are not influenced by the building itself [1].

In the case of historic buildings, one or more centuries old, both the meso- and the microclimate should have reached an equilibrium condition, that can include small natural variation; however higher change and or fluctuation of climatic parameters might occur due to restorations, excavations, reinforcements (sometimes with improper materials) or to the presence of humans (visitors, restorers) making measurement campaigns more complex. For conservation such fluctuations mean that, for example, a wall structure of stone, ceramics, glass, frescos, metals and also organic material which were conserved for centuries or millennia due to equilibrium conditions are put in danger in a very short time range compared to the sites life-time.

The present study concerns with a hypogean, which is a very particular part of a building and its investigation can be difficult; one reason is that - while identification of the spontaneous condition of the research environment is particularly important and fundamental for a conservation project - it is impossible to define *a priori* for a hypogean the time necessary to reach constancy or at least spontaneous periodicity of fluctuation, referring to Banham’s definitions [2].

Once macro- and microclimate are sufficiently defined, one can compare both in order to identify their correlation type, e.g. “Conservative Mode”, where the walls or the burying soil exclude or strongly slow down any exchange; “Selective Mode”, where some parameters are filtered by the barrier while others are not; “Regenerative Mode”, where an independent internal microclimate is created, which instead leads to retroactive mechanisms (typical for modern living environments with high waste of energy). For a hypogean the external construction often functions as filter for the macroclimate and might guarantee good microclimatic and air quality conditions. Two significant parameters, for conservation, are not studied in this research; the humidity distribution *inside* the walls [3] and the presence of microorganisms like fungi, bacteria, yeast, moss and also anaerobic organisms [4], both related to microclimatic conditions and necessary in a conservation project.

But what are good microclimatic conservation conditions? Ideal or limit values of microclimate parameters are defined in some official Italian publications [5] as well as in scientific ones [6], but knowledge on these values is still poor. Given that none of these values derive from an accurate (long-lasting?) experimental study with double blind technique as in the case of new pharmaceuticals and instead they are often the result of intensive expert-arguing [personal communication by one component of Normal commission], there is an urgent need for many, accurate, reproducible, comparable works, in order to avoid that one day microclimate parameters are imposed by law [7]. Unfortunately many papers on microclimate studies of indoor environments, especially in the field of cultural heritage conservation, could be discussed critically, because they do not state the type, number or position of the sensors used. Often the Design of Experiment (DoE), the result and instrument validation is not described. Generally few sensors, sometimes only one, are applied and time series obtained, which are then analysed by means of control cards and/or univariate graphs, but surely there is a correlation between temperature and humidity so a multivariate approach is necessary [8].

In this preliminary study we propose a, we think, sufficiently rigorous procedure, but for a too short observation period, where15 inexpensive but accurate data loggers were used for monitoring three environmental parameters (temperature, humidity, lighting) every 10 minutes along some vectors in a hypogean constituted by two rooms one above the other; chemometry was used to extract the most significant information.

## Experimental

The position of the building in the ancient Forum Romanum is shown in fig.A in additional file 1 (all figures in additional file 1 have a capital letter as index); it was probably built in the V. or VI. century b.C., above a water source.

**Figure 1:**
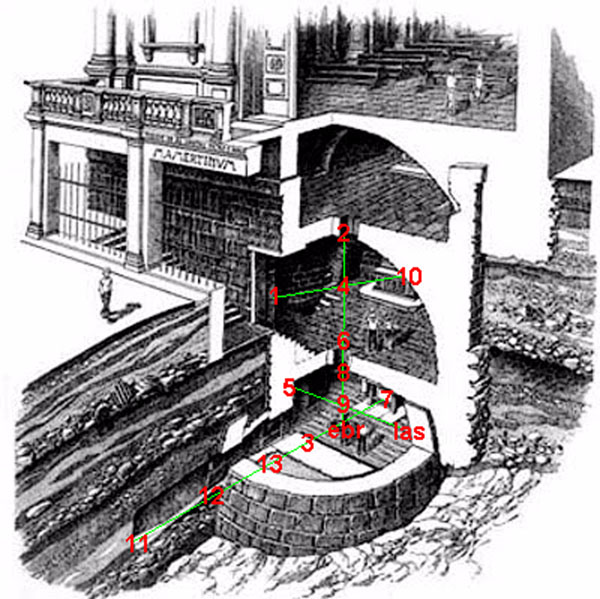
Sensors position according to the Sampling Design

The whole building is composed by a vertical series of rooms of which we monitored only the lower two, the Tullianum and the Carcer while our study does not include the chapel “Santissimo Crocifisso” and the church “San Giuseppe dei Falegnami”; complete information on the buildings history, structure and materials can be found in a paper of D.B.Kerner [9]. Currently the Carcer constitutes a partially buried church accessible by a descending stair with about 20 steps. The Tullianum, completely buried, had the floor continuously covered with a stratum of water the origin of which goes beyond the aims of this study [10]. In fig.1 the position of the sensors.

Basing on the archaeologists maps the dimensions of the two rooms were obtained; for the Tullianum wall thickness of about 1.7 m, an internal surface of ca. 25 m^2^ and a volume of ca. 56 m^3^ were found. For the Carcer walls thickness varies from 1.7 m (external) to 1 m (versus other indoor environments), surface is approximately 60 m^2^ and volume ca. 300 m^3^.

Luckily, the entire complex is not equipped with a heating, ventilation and air-conditioning system (HVAC), so, to ensure against anthropogenic variables, it was sufficient to close both rooms to public for the entire measurement campaign. The pump used to drain water from the Tullianum was set on automatic mode in order to avoid that the water level raised up some cm so ensuring against sensor diving.

For microclimate monitoring we used 13 Hobo U12-012 by Onset USA for measurements of humidity (RH%), temperature (°C) and Luminance (foot-candles or lumens/ft^2^).

A EB20-THP, by Ebro GmbH, Germany was also used as equipped with certified sensors for humidity (RH%), temperature (°C) and pressure (mbar).

In the preliminary coherence study (see paragraph Sensors calibration) two VWR cod. 61027-056, certified Hg thermometers AA grade, were used for controlling and calibrating of data loggers.

A LabQuest data station with sensors for CO_2_, O_2_ and temperature measurements was used as well (CO_2_-bta, O_2_-bta and STS-bta by Vernier Tech., USA).

Two Lascar data loggers (UK) were used for testing (EL-usb-CO for CO in air and EL-usb2 for RH%, °C).

In order to compare micro- and macroclimate, meteorological (macroclimate) data was obtained from "Osservatorio Meteorologico del Collegio Romano", active since 1782 and located in the centre of ancient Rome and on the top of a 63m (above sea level) tower, situated in 41.900°N, 12.480°E. Tullianum instead is situated in 41.893°N, 12.484°E.

Data elaboration software were mainly free ones, e.g. Past, Datalab, Gnumeric, WinIDAMS, Libre Office and some commercial ones like MVSP and Lotus 123.

To read data from data-logger producer software were used (available on brand web site). Only one software was not free and we apologize for this.

## Methods

Our work focused on the monitoring of two principle parameters, temperature (°C) and relative humidity (RH%).

As above said, an other parameter strongly influencing the conservation state of an object is bound to lighting. In a running research we are considering the effect of both the spectra of the light source and its wavelength-weighted power while in the present study only the last was monitored; illuminance was measured due to its greatest significance in studies regarding frescoes, paintings and any object in general.

15 data loggers were employed as passive samplers to monitor the three above said parameters; they were easily fixed also along vertical vectors (weight 46g) without disturbing the spontaneous environmental air flux.

When passive samplers are employed sampling frequency must be lower than the sensors rising time, so we choose a 10 min sampling time (see next paragraph) as the temperature rising time for Hobo is 6 minutes, see table 1.

**Table 1:** Comparison of the values of the 3 data loggers, according to the producer

	hobo			lascar		ebro		
parameter	temp	RH%	light intens.	temp	RH%	temp	RH%	mbar

accuracy	+/- 0.35	+/- 2.5	-	+/- 1	+/- 3.5	+/- 0.5	+/- 3	+/- 5
resolution	0.03	0.03	0.1	0.5	0.5	0.1	0.1	1
drift, one years	0.1	1	-	n.a.	1	0.5	1	5
rise time, min	6	1	-	0.2	0.1	5	2	1

Even if active samplers may be used at a higher frequency with respect to passive ones, we choose the last as, in our opinion, for a long monitoring period the adopted frequency allows to obtain significant information and, overall, the artificial air flux of known flow rate created by a pump and oriented on the active sensor [11], involves a self-heating especially in restricted indoor environments. Really, the self heating of a thermistor is a problem well known to electrical engineers, even a small 2W pump motor can modify the microclimate and thus measured values, when employed for longer periods.

In this first campaign an experimental data station with some sensors was also used in order to measure gas concentrations (e.g. CO_2_ and O_2_) in air. Further an attempt was made to evaluate the CO level as an indicator for contamination of the hypogean with external, urban, air. In order to identify the "spontaneous" values, the two environments were not entered during the 15 days measurement campaign.

### Sampling design

In this work the choice of vectors direction and position for sensor placement was based on Sampling Design (SD) using an expert-sampling among all authors for vector position; on each vector a systematic-sampling with even spaced distances including the extremes, was applied.

Position of the two main vectors is vertical (trespassing the two rooms, 6 sensor points) and horizontal (in the basement, 6 sensor points); the minor two vectors are one along a horizontal axis in the upper room (3 sensor points) from the door to the altar and one in the basement (3 sensor points) from the stairs to the opposite wall (fig.1).

The long horizontal vector is designed at about 50 mm from the floor and at the cross of the two vectors, the concentrations of CO, CO_2_ and O_2_ were measured too.

In the time domain we used a systematic-sampling with a reading every 10 minutes, but the choice of the proper sampling frequency was based on a preliminary 24h measurement campaign, using the faster available sensor, at 2 readings each minute.

Obviously immediately after the campaign one notices that some additional data logger somewhere would have been useful, leaving space for further campaigns and making work even more complex.

### Sensors calibration

Sensors control before and after measurement campaigns is a minimum requirement which is generally not stated in the microclimate studies on Cultural Heritage Sites.

Especially when inexpensive sensors are used problems could arise due to lacking of coherence of the single measurements; the same data-loggers are often available in a certified version, but as our aim was also the assembling of a inexpensive measurement system, the price of these latter was obviously too high. So, at the same cost we preferred a double number of data points buying more data-loggers and to verify by our own the sensors quality.

To do this, in a preliminary measurement campaign all 15 sensors where placed for a week in a hypogean ambient similar to the one to be studied, near to each other and around two certified instruments (the EB20-THP and VWR Hg thermometer); in this way we checked the sensors rising time, accuracy, hysteresis, and coherence before starting our measurement campaign. This allows to estimate the sensors response to environmental variations, e.g. a coherence of 0.5 °C allows us to regard a variation of 0.8 °C between two rooms or along a vertical axis as significant.

Once the sensors coherence was confirmed they were reprogrammed and positioned in the Tullianum for our study purpose.

At the end of our work all sensors were re-checked in the same way and in the same place and results are shown in additional file 1 as fig.B (temperature) and fig.C (humidity); the graphs show that no damages or calibration lacks occurred to the sensors during the measurement campaign because data are coherent considering the accuracy limit of the data-loggers (table 1). A Vernier data station was also used equipped with temperature, CO_2_ and O_2_ sensors (fig.D in additional file 1, CO trend is also reported); temperature data showed an offset of ca.0.5 °C and was therefore excluded even if used by the data station to apply the right correction factor to data coming from the two other sensors.

### Data treatment

The obtained data was put in a 3D-matrix composed by 16 columns, with sensors as variables, 2150 rows for each sampling time and 7 layers for temperature, RH, luminance, barometric pressure, CO_2_, O_2_, CO. Some layer columns are empty as not all data loggers can detect all parameters. For example the barometric pressure layer has only one column (Ebro sensor).

The above described matrix was then studied by Exploratory Data Analysis in order to identify any correlation of indoor and outdoor measured values as well as to identify the rooms spontaneous values.

Data of each measured parameter (layer) was plotted in monovariate graphs. Normally Run-Sequence plots of parameters (temperature, humidity, light intensity..) versus time or a function of time (here minutes from midnight of the first day).

In these graphs prefixed limits can be added and Shewhart Charts are obtained (also known as Control Charts), which enhance the environments characteristics.

The successive step in EDA is data analysis by Box & Whisker Notched Box Plots of a single parameter (a matrix layer), where variables (sensors) are plotted on the X-axis and raw measured distribution on the Y-axis.

This powerful analytical tool shows, here in the McGill [12] version, in the median domain, the distribution, spread, skew and percentile values 5% and 95% by whiskers; no outliers are shown. In our case only one matrix layer was plotted at a time so avoiding problems which can arise when variables with different scaling are plotted together.

Successively the three Scatter Plot Matrixes were designed showing up all possible Var-Var graphs between all variables (sensors), starting from raw data, in order to enhance correlations as well as distribution type of each variable on the diagonal.

On the diagonal of the Scatter Plot Matrix a histogram of ten bars is shown for each variable, which roughly allows identification of the distribution form; the values on histogram show the height of the major bar as the total is normalised to 1.

This important graph is often substituted by a correlation matrix, using the r^2^ Pearson coefficient; this latter works only on linear data, which is not always the case, especially in the cultural heritage sector (please see the complex column of Hobo10, on the altar, on the next figure).

In temperature and humidity layers there is also a column with macroclimate (outdoor) data; correlation of data in this column and those of the others is possible and plausible. However macroclimate data has a 30 minutes sampling interval; therefore a new matrix was constructed in which the indoor data was downsampled in order to allow better comparison of indoor and outdoor data.

Correlation of variable couples was then analysed and this was done not only by computing the common r^2^ coefficient, which may not be sufficient. An example of calculated regression parameters can be seen in table 2 for barometric pressure.

**Table 2:** Evaluation of the delay between internal and external barometric pressure studying the lag correlation.

parameters \ lag (min)	0	30	60	90	120	150	180
sample covariance	13.644	13.631	13.593	13.547	13.496	13.437	13.369
population covariance	13.625	13.612	13.574	13.528	13.477	13.419	13.350
Pearson product-moment correlation coefficient, sample	0.990	0.990	0.988	0.986	0.983	0.980	0.976
square of the Pearson correlation coefficient	0.981	0.980	0.977	0.972	0.966	0.960	0.952
assess of Std Err of Pearson's r	0.005	0.005	0.006	0.006	0.007	0.008	0.008
variance of post-fit residue	0.259	0.270	0.317	0.380	0.451	0.536	0.640
residue sum of squares (RSS), sample	183.467	191.739	224.471	269.190	319.809	380.278	453.889
root mean square deviation (RMSD), sample	0.508	0.520	0.562	0.616	0.671	0.732	0.800
standard error of the Y estimate	0.509	0.520	0.563	0.616	0.672	0.732	0.800
computed Slope	0.972	0.971	0.968	0.965	0.961	0.957	0.952
computed Constant	27.831	28.801	31.486	34.821	38.492	42.711	47.662

If one plots in graphs parameters which best describe correlation, in terms of quality, here rows enhanced in blue, it is possible to compare two or more correlation, which differ for instance for the data amount (many validation techniques like LeaveOneOut or Bootstrap are based on these descriptors).

A building isolation can be evaluated also by Loss/Gain for Transmission graphs, where internal and external data are shown and compared to a perfect correlation which is shown as graph diagonal. Any sign above the diagonal is a gain of the building compared to the outdoor environment, e.g. can be due to a heating system for a temperature graph. By using labels for single data points these graphs allow also evaluation of hysteresis, spontaneous behaviour, changes with time. The sensors accuracy is fundamental in these graphs.

A temperature-humidity correlation study was the next analysis step. Thus two columns of two overlaying layers were compared and for each point variation of both parameters as well as the 98 % confidence ellipses were obtained.

All these data exploration methods together allow evaluation of the buildings isolation from the outdoor environment, as well as enhancing internal gradients; further spontaneous values, not normal behaviour and sudden changes, which are the most dangerous events for a cultural heritage site, can be evaluated.

## Results

### Time plot

**Figure 2:**
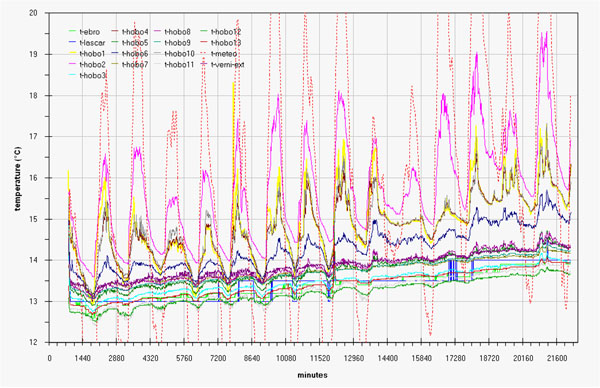
Trend of the temperature during the measurement campaign by all the sensors and comparison with macroclima

**Figure 3:**
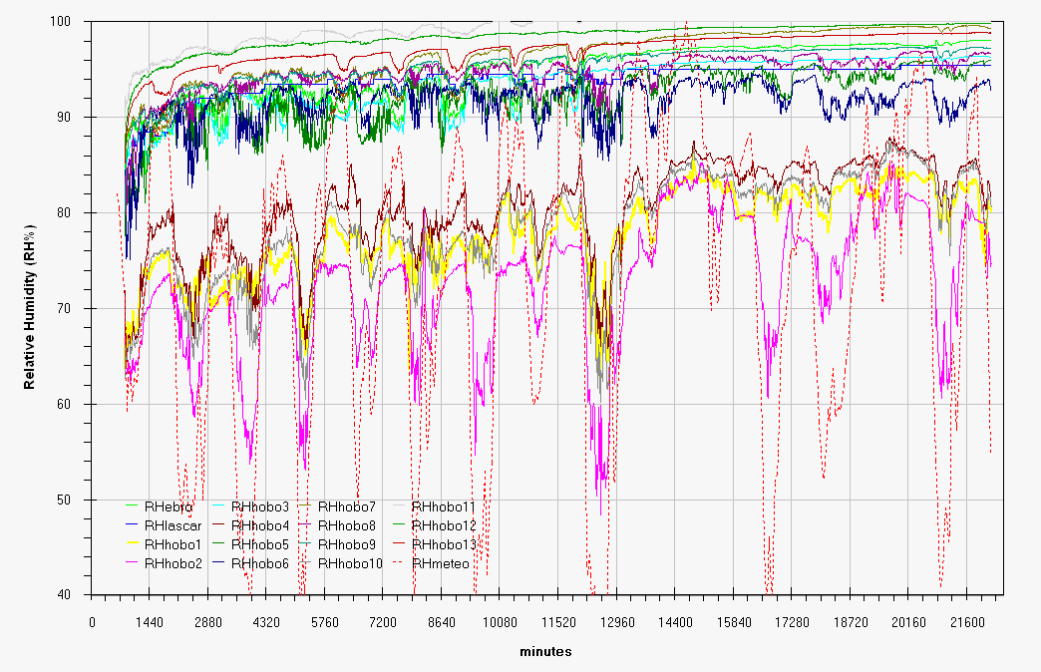
Trend of the relative humidity during the measurement campaign by all the sensors and comparison with macroclima

Microclimate monitoring results are represented, as run-sequence-plots, in fig.2 (temperature in degree Celsius) and fig.3 (relative humidity percentage). In order to allow better data overlapping, macroclimate data (red dotted line) were added in the same graphs (red scale on the right).

These simple graphs already enhance:

- a more or less evident correlation between indoor and outdoor (macroclimate) data.

- a certain delay, more evident for humidity data, for indoor changes with respect to outdoor changes.

- as expected, the two rooms behave differently; the hypogean is less influenced by macroclimate and show a lower variability for both the measured parameters.

- a tendency versus a spontaneous microclimate, more evident for humidity so putting in evidence the need to extend in time the next measurement campaign.

- finally, the coherence among sensors was checked before and after the campaign, (examples of the latter in fig.B and fig.C in additional file 1), allowing to consider all variations shown in graphs of fig.2 and fig.3 as significant.

- careful graph observation allows stating that for these two different rooms the attempt to draw univariate Shewhart or Control Charts and define limit values to be respected by a HVAC would be confusing and stupid.

**Figure 4:**
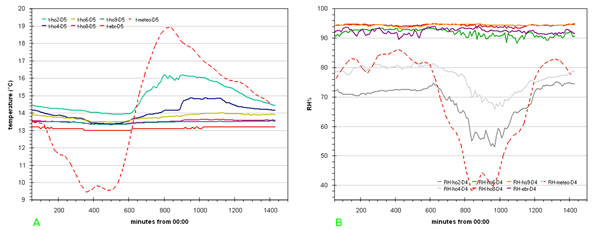
**Daily variation evidencing the delay with respect to macroclima of** a) temperature; b) relative humidity

In order to improve data reading one can draw a series of graphs each one relative to a single day. As an example, in fig.4(a,b) temperature and humidity data, along the vertical vector for day 4 and 5 respectively, enhance the parameters different behaviour according to Benham [2] and the above said delay.

### Study of the values distribution

For remarking of each distribution and mutual behaviour Box&Whisker plots can be used starting from raw data.

**Figure 5:**
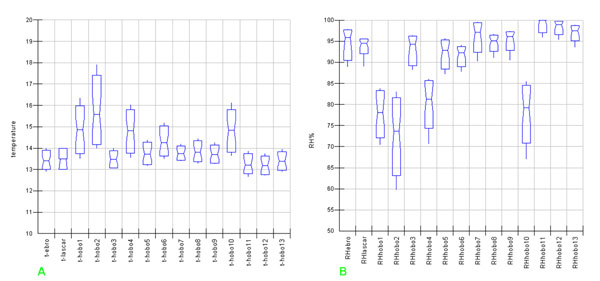
Box&Whisker plots for temperature (a) and relative humidity (b) measured by all the sensors

Including all sampling points the graph in fig.5a (temperature) is obtained where two main groups can be observed, each one including the sensors located in each of the two different room. Really, sensors 1, 2, 4 and 10, located in the upper room, behave completely different; wider ranging values and higher absolute values for the medians in comparison with those located in the Tullianum (ebro, lascar, 3,5,7,8,9,11,12,13) are clearly evidenced. Sensor 6 is also located in the Carcer but just on the grid connecting the two rooms and it is not so easy to insert it in one of the two groups. More in this group the sensors which are part of the horizontal vector (1, 4, 10) behave similarly for what concerns both the median value and range while on the vertical vector the expected trend can be observed; really, coming down, the graph shows a decreasing trend for both median values and spread as well as decreasing differences between points. Along the main horizontal vector of Tullianum a very similar behaviour is evidenced with the exception of point 7 that shows a slightly lower spread and higher median while on the minor vector temperature is almost constant but ranges slightly different.

In fig.5b, the humidity distribution evidences the same two main data groups well separated; one characterised by wider ranging values and a lower median (points 1, 2, 4 and 10) and another one including all remaining data points. Sensor 6, in this case, is clearly included in the Tullianum group even if, together with sensor 5, located on the stairs connecting the rooms at about 2 m from the lower floor, shows a similar median value enough low with respect to all the others. The presence of the subgroup including sensors 11, 12 and 13 is also better evidenced; such sensors are positioned on the main horizontal vector, inside a tunnel probably coming versus the Cloaca Maxima and connected to the room through a metallic door that was left open during the campaign.

### Correlation among points

**Figure 6:**
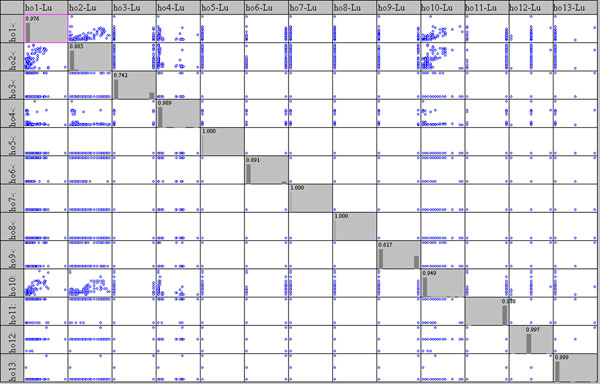
Scatter plot Matrix for luminance measured by all the sensors

As described in methods section, Scatter plot matrix were designed in order to detect possible correlations among the columns of the single matrix layers. Starting with luminance data in fig.6 one observes that only sensors n° 1, 2, 4 and 10 show a series of values (see columns I, II, IV and X); values of sensor n° 4 are distributed in a very narrow range and point 6 has only two values, confirming that the used data loggers do not have a LOD sufficient for the luminance measurements due to the scarce photon number in this hypogean.

**Figure 7:**
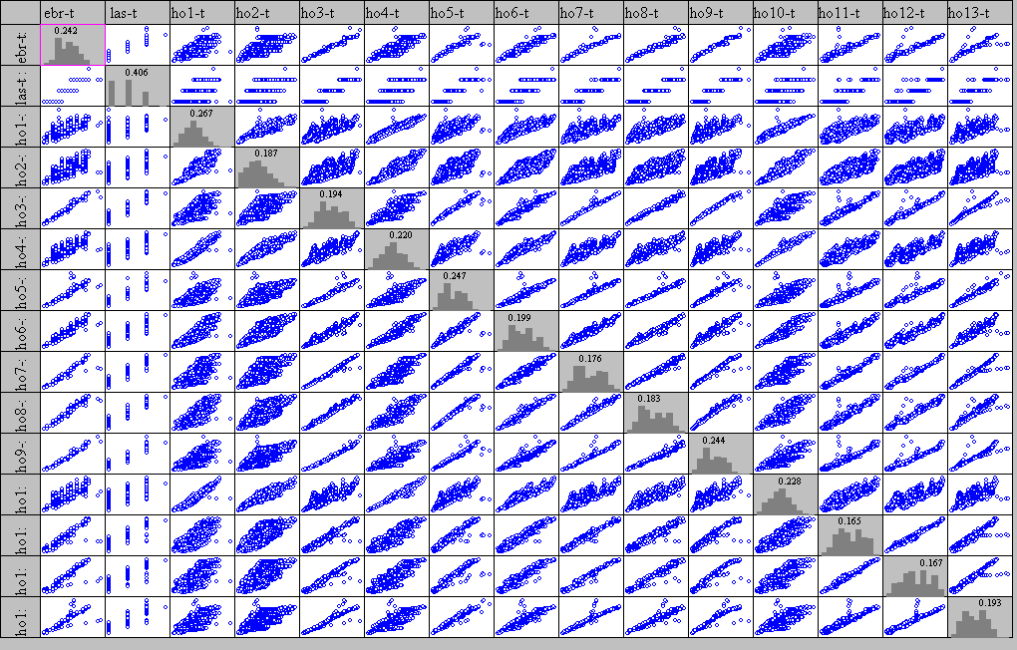
Scatter plot Matrix for temperature measured by all the sensors

Fig.7 shows the matrix of temperature plots and it enhances immediately the low resolution of the Lascar sensor (very similar values compared to those measured by the other sensors). All other sensors show a more or less evident positive correlation. Sensor n° 8 for example, situated below a grating connecting the Carcer and the Tullanium, shows a high linear correlation (r^2^ = 0.9819) with point n° 6 and all Tullianum points; a significatively lower correlation with Carcer points is evidenced (r^2^ = 0.704 with point n° 2).

**Figure 8:**
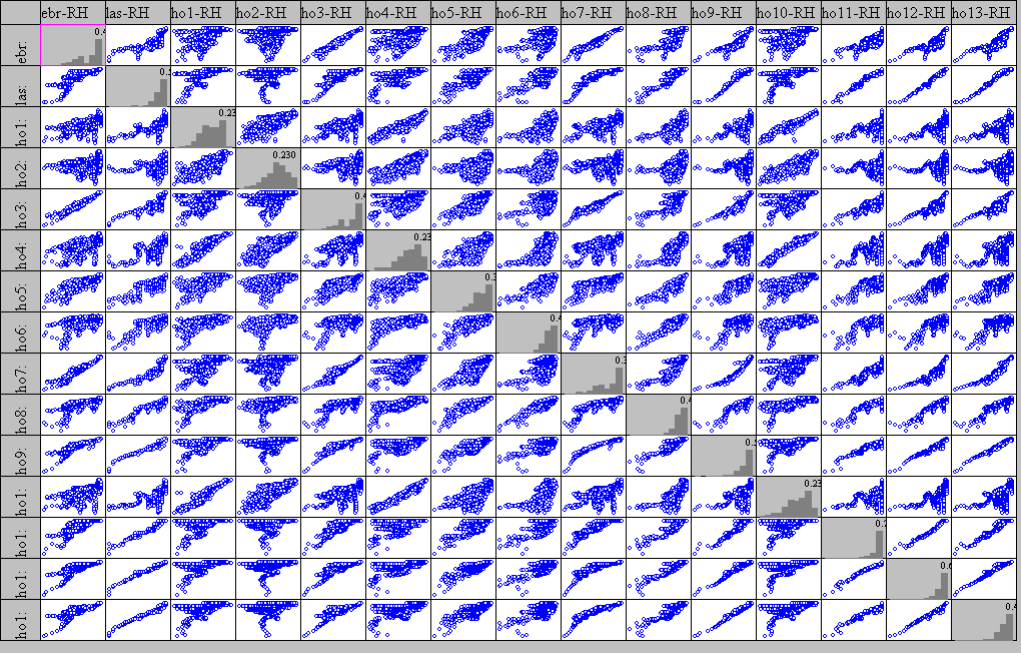
Scatter plot Matrix for relative humidity measured by all the sensors

In fig.8 the scatter plot matrix for relative humidity is shown; even if less evident with respect to fig.7, a positive correlation among sensors can be observed as well as a wider variability for the entire matrix. It can be noted that, as expected, sensors 11, 12 and 13, located in the tunnel and on the Tullianum main vector are well correlated with each other and also with sensors 9 and Lascar that are located on the same vector; even if sensor 3 is also located on the same vector and closer to the tunnel, a lower correlation is evident that can be attributed to a position close to the stair that allow a higher air flow as already evidenced above.

Analysis of the structure’s isolation from the external environment cannot be performed by a simple scatterplot, instead one has to calculate all correlation parameters [13] among two columns of the same layer.

### Daily variation

Before starting lag-correlation analysis it seems useful to identify time-windows hidden in time-series (repetition of the same values with a certain frequency due to cyclic phenomena such as night-day or seasonal variations). A number of software allow detection, expensive ones and free ones like Past [14].

Time series analysis are performed in the same way as Spectral Analysis, starting with Fourier Transformation which allows shifting from the time domain to the frequency domain. For temperature and RH data series of sensors 2 (top of Carcer) and 8 (top of Tullianum), a time window of 1430 minutes is detected for the first peak of the FFT spectra.

Usually in combination with spectral analysis Sinusoidal Regression (often called Sinusoidal Curve Fitting, SCF) is used, where a sum of *n* sinusoids with specified periods is used to fit a known list of time dependent data. Past algorithm of SCF is based on a least-squares criterion and singular value decomposition.

This can be useful to check and model periodic phenomena in time series, as in our case. Further it is interesting to compare the results of FFT and SCF in order to obtain result validation and to avoid overfitting.

Expressing the identified time window in hours, one finds a values close to 24 (1440 minutes), which confirms influence of the external environment on our indoor ones.

To check fast change in values (temp. RH, lux) we must use a sampling frequency more than two times faster than the change under study (Nyquist–Shannon sampling theorem), but this is difficult with a sensor with rise time of 6 or 10 minutes, one other big problem in microclimate monitoring often silenced.

### Lag correlation

The meteorological data used had a 30 minutes sampling interval, thus our measured microclimate data had to be reduced by downsampling as described in methods section.

For correlation analysis of meteorological data (outdoor, X axis) and measured microclimate data (indoor, Y axis) Ordinary Least Square was used; data of the Y axis was then shifted by 0, 30, 60, 90 and 120 minutes and obtained parameters registered in new matrixes, like the one in table 2.

Correlation coefficients were calculated for all available data without using a time window in order to avoid any anomaly. If number of data is much higher (10-30 times) than the width of time window this techniques is sufficiently robust to avoid dependence from some not normal data (e.g. presence of a small heater on day 12 or a window open all night on day 18).

In contrast to other works our first approach was data validation and results coherence; correlation analysis of barometric pressure (mbar) of Tullianum data and meteorological data was performed first. As no HVAC is present in the Tullianum, barometric pressures must be equal, see fig.F in additional file 1.

In table 2 obtained coefficients are shown. A value of r^2^=0.9665 is observed, which is very good considering the distance of the two measurement points (850m) and the different instrument types and cost.

Further, lag-correlation is maximum for t=0 while all blue values worsen the situation, introducing a delay on the values of the Y axis, which are those measured in the Tullianum.

Computing all correlation parameters listed in table 2 for all space points, for temperature and humidity data, graphs containing the data referring to all vectors can be draft.

**Figure 9:**
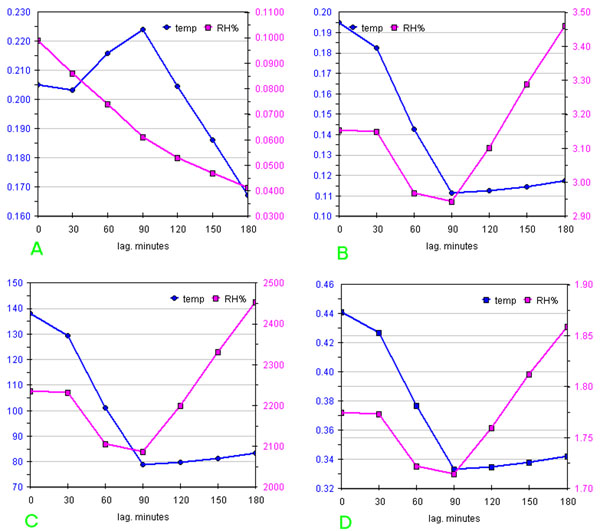
**Trend of the correlation parameters as a function of the delay between measures performed by sensor 8 and macroclima**: a) Pearson’s coefficient of determination, r^2^; b) variance of post-fit residue; c) residues sum of squares (RSS); d) root mean square deviations on residue (RMSD).

As an example figs.9(a-d) show a series of graphs obtained from Table 3 (in additional file 2), using parameters marked in blue, for temperature and humidity measured by sensor 8, situated below the communicating hole of the two rooms. All parameters evidence a delay for temperature equal to 90 minutes, but with a determination coefficient r^2^=0.224; thus only 22.4 % of the variance of Y can be explained by X, this means that the system must be considered an almost completely isolated one.

Humidity always shows the same delay with a r^2^=0.061, thus X and Y must be considered independent.

A simple visual comparison is not sufficient and certainly the common analysis of delays between minima and/or maxima (if detectable) is not useful in order to obtain a correlogramm.

The lag correlation is already evident in figs.4a and 4b, i.e. the line plots of the raw data obtained with a 1440 minutes time window for temperature data on day 5 and for RH data on day 4 respectively (days 4 and 5 were chosen due to strongly varying values of parameters).

The next step in our analysis on the building was to show up r^2^ values and slopes of the linear correlation of each main axe obtained by comparing outdoor and our indoor sampling points using a proper delay prior identified as above described.

**Figure 10:**
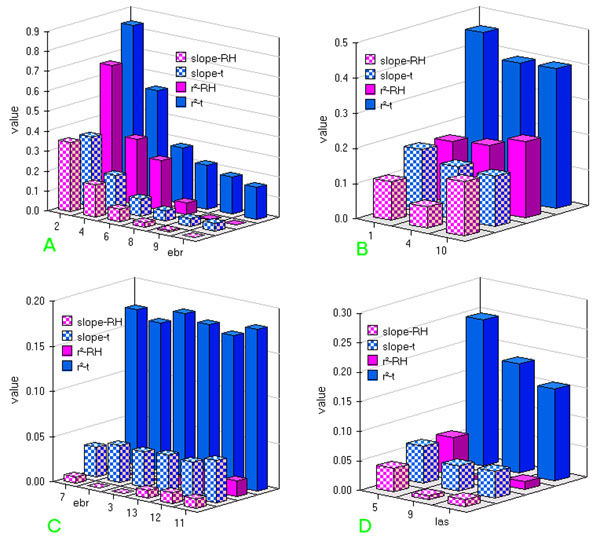
**Slope and r^2^ trend for the best correlation among delay relative to**: a) vertical vector; b) Carcer horizontal vector; c) Tullianum main horizontal vector; d) Tullianum minor horizontal vector.

In fig.10a, trends for r^2^ and slope values, relative to the correlation between macro- and micro-climate on the vertical vector are shown. For both temperature and humidity, it is well evident a decrease starting from position of sensor 2 for both parameters and, as expected, starting values significantly higher as well as a quicker decrease for sensors located in the Carcer.

Fig.10b shows lower values for both r^2^ and slope and a less evident trend for both parameters relative to the series of the shorter horizontal Carcer vector.

A flat trend and a further decrease for both slope (close to zero) and r^2^ values can be seen in fig.10c and d relative to the two horizontal Tullianum vectors, remarking that the ambient has to be considered an isolated one.

For all the vectors, lower slope and r^2^ values are evidenced for humidity with respect to temperature; such behaviour is consistent with a greater influence of the Tullianum humidity with respect to the macroclima.

### Insulation

Rooms isolation can be estimated as well by a Loss/Gain representation; firstly a daily mean is calculated for both the outdoor (meteorological) and the indoor data (the latter for both the Carcer and the Tullianum separately, considering all the relative sensors) then correlation graphs were drawn for temperature and humidity data.

**Figure 11:**
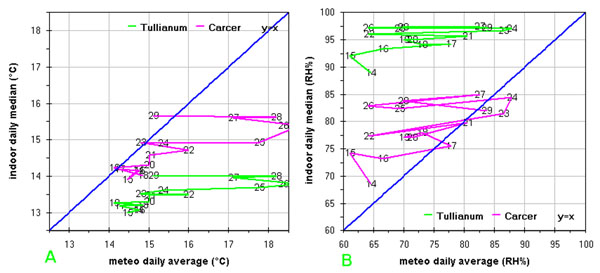
**Correlation between macroclima and microclima (medium values from all the sensors located in the Carcer or in the Tullianum):** a) temperature; b) relative humidity

Fig.11a and 11b shows the temperature and humidity graphs. On temperature, with few exceptions (as an example Carcer on days 19, 29 and 23), both the Tullianum and the Carcer show lower values compared to the external ones. Similar daily variations, enhancing communication between the two rooms, can be seen for Carcer and Tullianum; more, lower variations can be seen in the order: outdoor>Carcer>Tullianum (i.e. maximum variation result about 5, 2 and 1 °C respectively), enhancing the expected higher isolation for the Tullianum.

To understand at the best the above behaviour, it is useful to consider that the position of the building entrance, that is the only point in direct communication with outdoor through an open-work door, is east-south-east oriented, so, sunlight can enter from about 8 until about 14. Further, it must be pointed out that Tullianum is a classical hypogean but also the Carcer is partially underground; as a consequence, illumination level is low everywhere as can be seen in fig.E in additional file 1 where only the upper sensor on the vertical vector (Hobo2) seems to be stimulated. On the other hand the underground position of the Carcer allows a lower influence of eventual external wind through the open-work door. Finally, all the walls are very thick so ensuring a good insulation.

An expected opposite trend, i.e. value are above the outdoor ones, is shown in fig.11b for humidity with the exception of data relative to Carcer on days 17, 21, 23, 24 and 29. The same flatting trend on variations, above seen for temperature, is evidenced with maximum value of about 25, 18 and 10 % for outdoor, Carcer and Tullianum respectively. Really the presence of groundwater ascending in the Tullianum and the huge walls funded directly on the water layer cause high internal humidity levels, like the one shown in the final part of graph in fig.3; any reasonable conservation concept should not interfere on this.

### Multivariate

**Figure 12:**
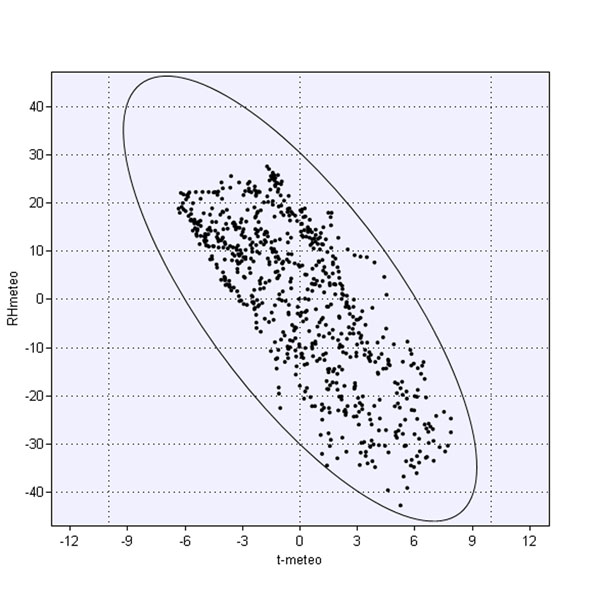
Correlation graph between outdoor temperature and relative humidity

The reply at the question “is there a correlation of humidity and temperature?” is obviously “yes” but it is not unique and exhaustive. A first answer can be based on fig.12, showing a scatterplot, after column centering of the outdoor data (humidity versus temperature) during our measurement campaign; a not so clear reply can be given considering the indoor data.

Column centering is an important data pretreatment because it leads to scatterplots which show directly the swing of the values on the axes; for fig.12 we obtain about 14 °C for temperature and about 70% for humidity with a negative correlation. Further outliers, trends and leverage are easily enhanced while centering does not modify distribution structure; instead it can lead to different results in the successive EDA phase [15].

In order to enhance the study of the distribution form for temperature and humidity data for each sensor, one can compute bar charts with class distribution. Choice of class width is not a trivial task, should consider values well above the instruments resolution level and producing a number of classes ranging from 10 to 30. Here we chose a width of 0.5 °C and of 2 % for temperature and humidity respectively, but different choices can change "the shape".

**Figure 13:**
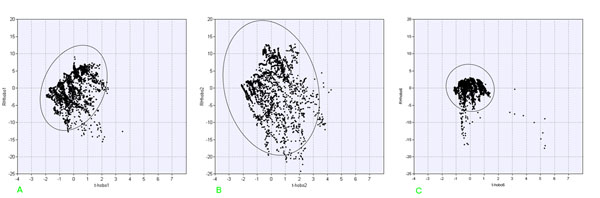
**Correlation graph between indoor temperature and relative humidity measured by:** a) Hobo 1; b) Hobo 2; c) Hobo 6; d) Hobo 5; e) Hobo 8; f) Hobo 11

**Figure 14:**
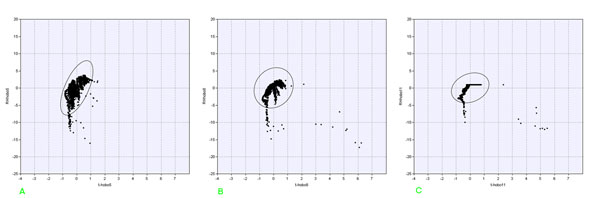
**Distribution of microclima data measured by Hobo 1:** a) temperature; b) relative humidity

**Figure 15:**
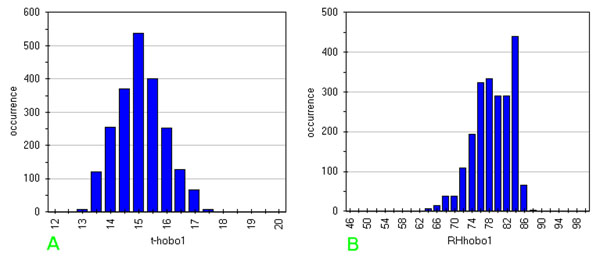
Run Sequence Plot of humidity measured by Hobo 6 with a sampling interval equal to 5 min

Multivariate representation like that in fig.13 for Carcer and fig.14 for Tullianum was calculated for each measurement point and also the class distribution was draft. In figs.15a and 15b the distribution of graphs for the sensor n° 1 (behind the door, see fig.1) are shown. Confidence ellipsis of fig.13a (and of all the other ones here reported) was calculated for 98%; it shows an enough wide variation of both parameters (spread equal to 5.4 °C and 24 %RH) and no clear correlation that anyway seems to be more positive than negative; class distribution of temperature, shown in fig.15a results enough regular with a maximum around 15 °C while in the humidity graph (fig.15b) higher value are significatively prevalent.

The complete series of figures are available in additional file 1, from fig.G-a to fig.V-c.

Among this huge amount of data, one should focus on data from sensor 2 (fig.13b), located at the top of the vertical vector (see fig.1); a wider ranging value for both parameters is evident with respect to the previous sensor (spread equal to 6.4 °C and 37%RH) and a no clear correlation could be seen.

Interesting is also point 5 (located in the lower part of the inner stair, see fig.14a; this figure shows a narrower and high sloping ellipsis with respect to the previous two; it must be also noted an enough crowded queue, out of the ellipsis, where a humidity variation of about 5% corresponds to an almost constant temperature. Humidity spread results equal to 20% so being no so different from that of sensor 1; an other similarity with such sensor is the positive correlation while temperature spread results significatively lower and equal to 2.3 °C. Looking for a similarity due to the position, it can be noted that, with respect to Carcer, sensor 1 is the nearest to outdoor and sensor 5 is the nearest to Tullianum; it is evident that macroclima have the maximum influence on the first while the effect of the very humid Tullianum is higher on the last. In fig.13c, relative to sensor 6, one can observe a near spherical shape for the ellipsis and a queue, similar to the one discussed above; in fig.14b, relative to sensor 8, a narrower shape along y axis results from the grouping of points inside the ellipsis and the external queue is more similar to the one in fig.13c. The two sensors are located above and below the hole connecting the two rooms and the above trends evidence the influence of the high humidity inside Tullianum on that of the Carcer. Basing on fig.14c it can be stated that, as expected, no influence from the macroclima is evidenced by sensor 11, located in the farest position inside the tunnel toward Cloaca Maxima; in particular, humidity is practically 100% for most of the points (see fig.R-c in additional file 1).

The class distributions for all the sensors (fig.G to fig.V, letters b and c in additional file 1) show a decreasing number of classes in the order meteo>Carcer>Tullianum with decreasing temperature and increasing humidity in the same order.

**Figure 16:**
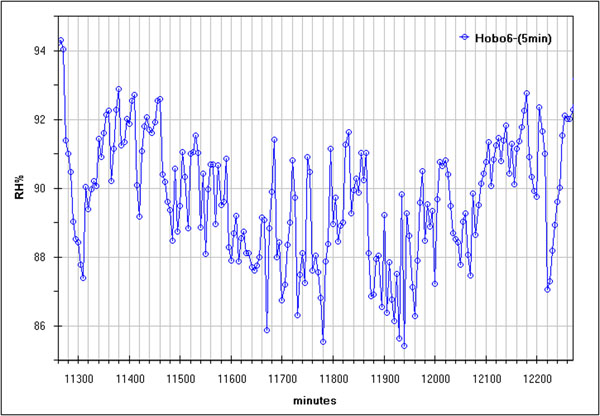
Fourier Transformer of all data measured by Hobo 6 with a sampling interval equal to 5 min

**Figure 17:**
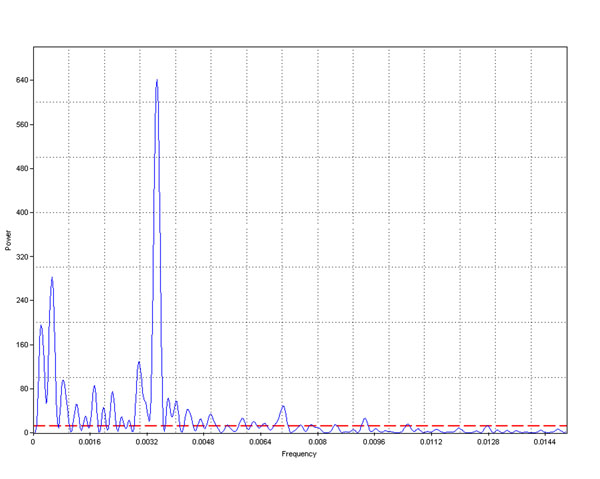
Run Sequence Plot, (after row centering) of humidity measured by all sensors during the last two days of the campaign.

As well known and already said in the introduction, major suffering of any ambient is due to the rapid changes of microclimatic parameters which cause a series of chemical and physical processes in the structures as well as in mobile objects; instead, all slow changes are certainly sustained by materials which survived in these environments for 300 or 2000 years. In order to detect eventual rapid changes FFT and SCF can be very useful. An example is given in fig.16 (humidity from sensor 6 with 5 min. sampling interval) where some enough rapid changes are evidenced. Such changes can be seen better using the FFT, in fig.17 the already identified daily change is well evident but also some less frequent variations at both lower and higher frequency result significative (red line refers to 95% confidence).

## Conclusions

Referring to the Tullianum the main exchange with outdoor seems to take place along the stairs and not through the hole in the ceiling. Really figs.5(a-b) evidence significative differences between sensors 6 and 8 that are located, at only about half meter each, above and behind the grid connecting the two rooms; this can be explained with a scarce air flow through the grid and/or with a bigger influence of the Tullianum microclima on sensor 6. An air flow from the tunnel coming toward Cloaca Maxima, together with a water layer often present on the floor, seem to be the main responsible for the Tullianum microclima; anyway, also an air flow from the stairs plays a role as humidity never reaches 100% that, on the contrary, is the almost constant value evidenced in the tunnel. More, the low macroclima influence is also evident in figs.2 and 3.

**Figure 18:**
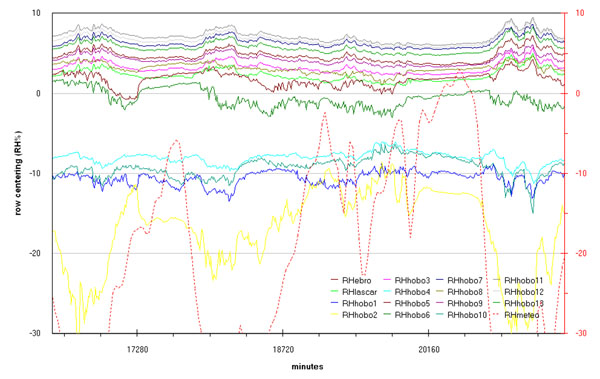


Taking into account that the building was always closed during the measurement campaign, figs.2 and 3 evidence a trend coming toward spontaneous conditions with lower and at lower frequency variations for both temperature and humidity, in fig.18 humidity data registered from midnight of 26 April until the end of the measurement campaign evidence at the best what said above. Matrix layer was subjected to row-centering to enhance the difference among sensors.

In fig.18 the same trend is evidenced for the Carcer except for sensor 2 that, as already seen, is located in the position most influenced by the macroclima. Such figure also reveals that spontaneous values flat toward values more closer to those of Tullianum probably as a results of the good insulation.

## But the main question of a conservator is: such conditions are safe for the artwork?

We cannot give the right reply! Really this paper can give an input to perform a monitoring at the best but time was too short and, as a consequence, no seasonal but only daily variations were obtained; further, it was performed not in real conditions that foresee the entrance of visitors with all the consequent problems. Probably a longer monitoring would allow to obtain more accurate values for the spontaneous conditions an surely in such conditions the bigger damage due to variations can be eliminated, but who can ensure that spontaneous conditions would be the best for all the materials present in the building? Really in the building are present different tuffs, frescoes, stone column …; each material has different chemical-physical characteristics so being differently influenced by the environment. Lastly but not in importance: what conditions can ensure against damage from biological source? The presence of visitors allows microorganism entering the building, increase the carbon dioxide concentration and so on, but the absence of visitors cannot ensure that no biological damage can occur. More, in our case we have a sure capillary rising that can allow presence of salts that must be taken into account.

Probably is exact the statement of F.G. Ojea is exact "*la conservación de las pinturas exige un mantenimiento de la temperatura y humedad a niveles lo más constantes posibles*, *y de un orden aproximado al que tenía el monumento antes de ser abierto*, *condiciones óptimas para su conservación*, *y que*, *obviamente*, *si fueran desfavorables no permitirían que el monumento llegara hasta nosotros*” [16] and it is applicable to our hypogeum.

What must be done? A correct conservation project foresees a perfect knowledge of all the materials that must be saved and of all the surrounding to find a compromise between the best environmental conditions for all. In our case, surely frescoes are the most precious artworks and also those more sensitive to damage; surely the microenvironment must take into account this. A compromise must be adopted to allow visitors to enter the building but surely microclima cannot be projected for their well-being, as stated in some book.

In practice the right procedure is running as the Sovrintendence asked for cooperation to “La Sapienza” University to carry out analysis of constituent materials, map of salts and microclimate monitoring.

For the last a new campaign is running in the same seasonal period to evaluate the influence of visitors. Other rooms will be monitored to evaluate the reciprocal influence and seasonal campaign will follow.

Also citing by reference [17] "…*not only is the relationship among all the variables at any one time important*, *but so is the entire past history of the trajectories of all these variables.*" confirm the use of multivariate analysis as necessary [8].

We may conclude this work stating that careful conservation of an entire ambient, here hypogean, declared cultural heritage, should foresee a triple action:

a) identification of real conditions microclimate over a long time-scale, with season changes and lasting possibly years;

b) comparison of the identified conditions with those indicated in official publications (see [18] for a list) in order to plan possible site tailored interventions, preferring passive methods;

c) publication of the done work in international journals in order to allow wide knowledge diffusion; work description should include details on measurement procedure AND RAW DATA to allow eventual further data elaboration by of other researchers.

## Competing interests

None of the authors received any financing, scholarship, contract or consulting from societies mentioned in this paper. Instruments were bought regularly through national research funding.

## Authors' contributions

Among the authors, who contributed in the same manner to this work, we would like to differentiate S.D.G., our graduating student, whose thesis is focused on the chemical/physical characterisation of some Tullianum parts; further P.F. is responsible for the cultural heritage site for the Roman City Council, who contributed with information on the historic/artistic aspect. The remaining authors contributed in a different manner but at the same level to this work.

## Supplementary Material

Additional file 1 This contains all the figures with letters as fig.A, fig.B cited in the text and necessary to better describe the microclimate of the building. Click here for file

Additional file 2 Here are stored the raw data obtained from every sensor, with also all statistic parameters calculated for every column of the main matrix, so for every distribution. In this file there are also a copy of the tables cited in the text. The two files has been checked, for open, with the major free or open source software as MSviewer 2003, LibreOffice, Smartsuite 9.x, with some OS. Click here for file
