# Supplementary material for: Microclimate monitoring in the Carcer Tullianum: temporal and spatial correlation and gradients evidenced by multivariate analysis; first campaign
Source: Chem Cent J. 2012 May 2;6(Suppl 2):S11. doi: 10.1186/1752-153X-6-S2-S11 (PMC3342126; doi:10.1186/1752-153X-6-S2-S11)
Supplement: Additional file 1 — This contains all the figures with letters as fig.A, fig.B cited in the text and necessary to better describe the microclimate of the building. [file 1752-153X-6-S2-S11-S1.pdf]

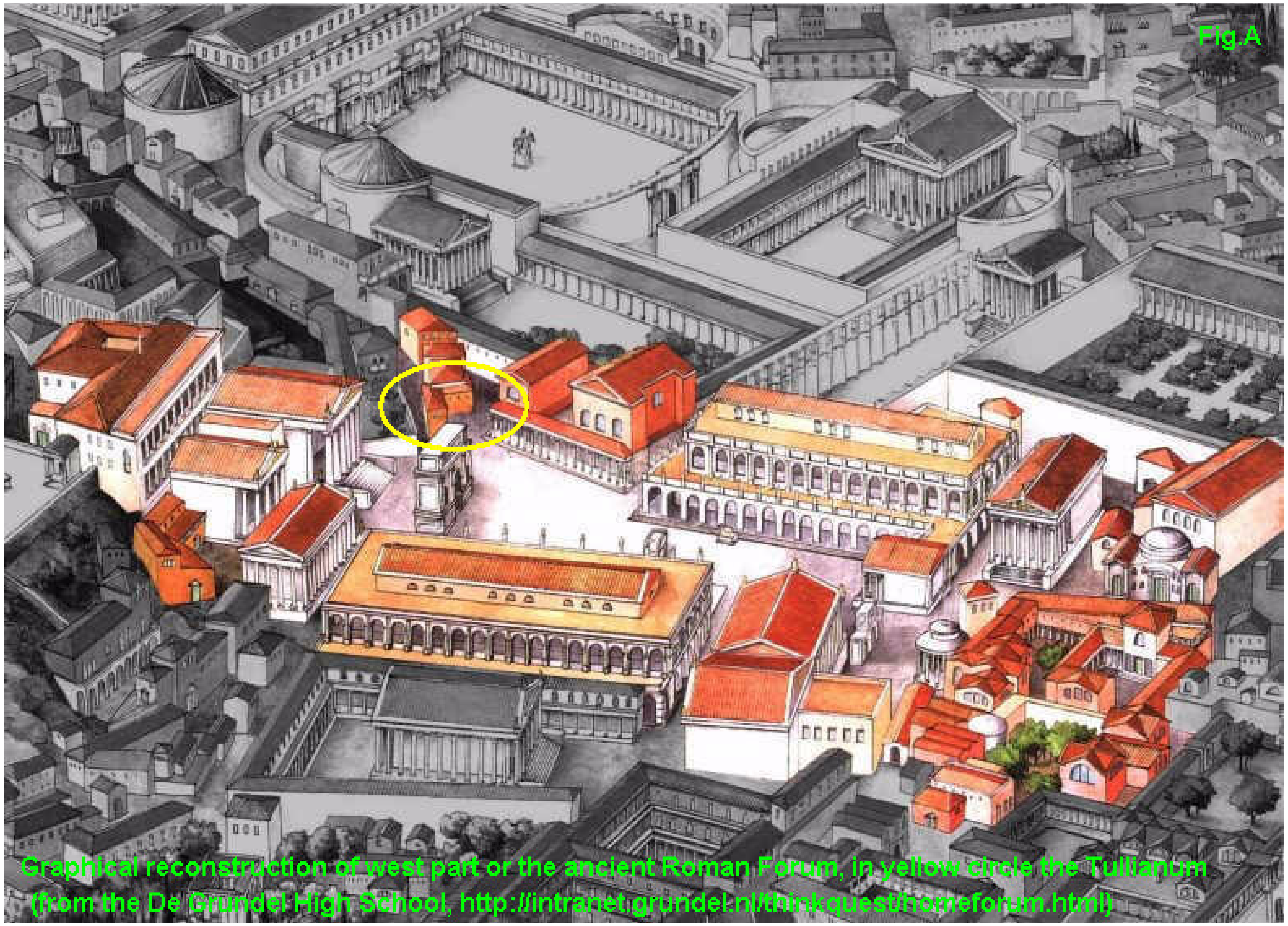

Fig.A

Graphical reconstruction of west part or the ancient Roman Forum, in yellow circle the Tullianum
(from the De Grundel High School, http://intranet.grundel.nl/thinkquest/homeforum.html)

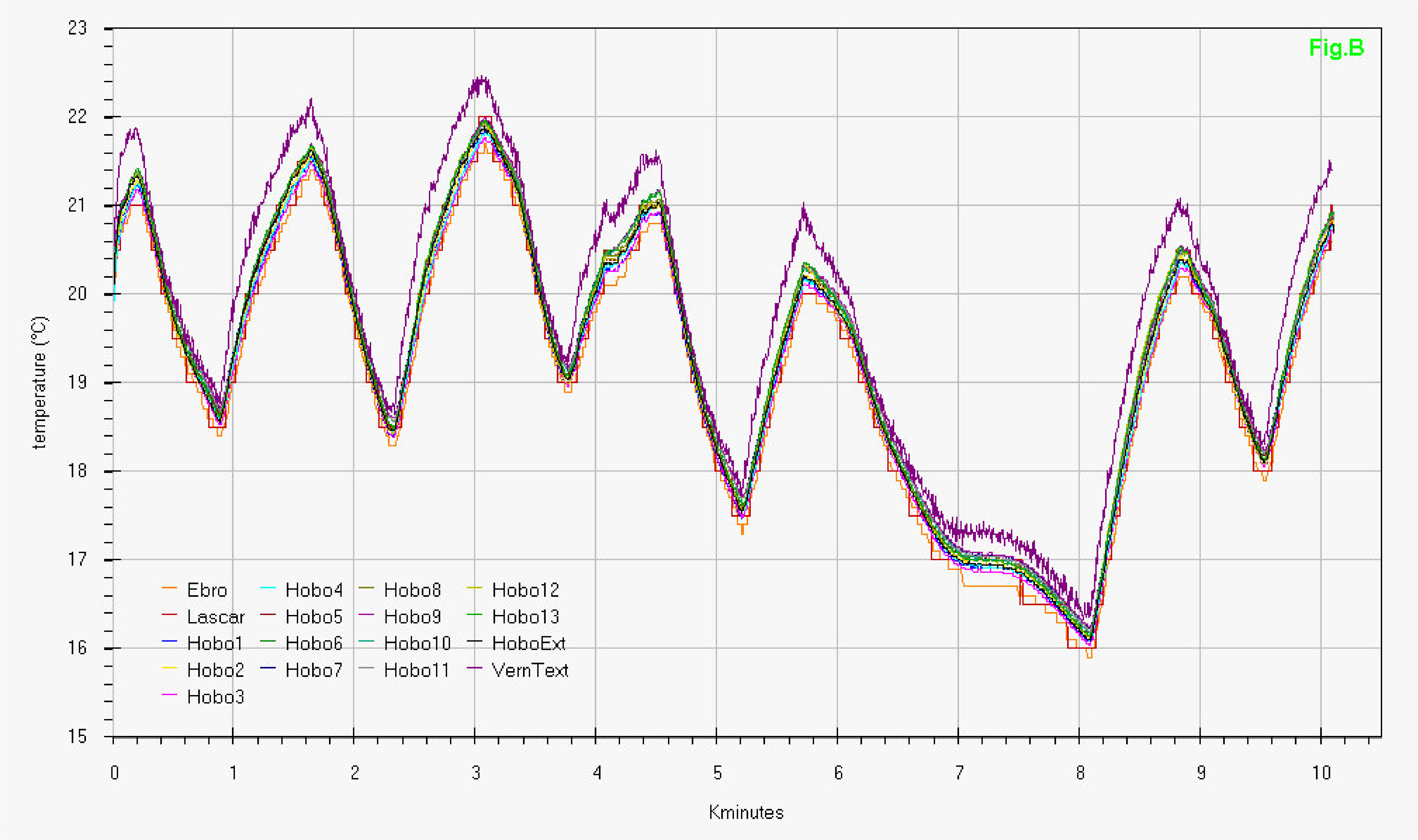

Fig.B
temperature (°C)
Kminutes
23
22
21
20
19
18
17
16
15
0
1
2
3
4
5
6
7
8
9
10
Ebro
Lascar
Hobo1
Hobo2
Hobo3
Hobo4
Hobo5
Hobo6
Hobo7
Hobo8
Hobo9
Hobo10
Hobo11
Hobo12
Hobo13
HoboExt
VernText

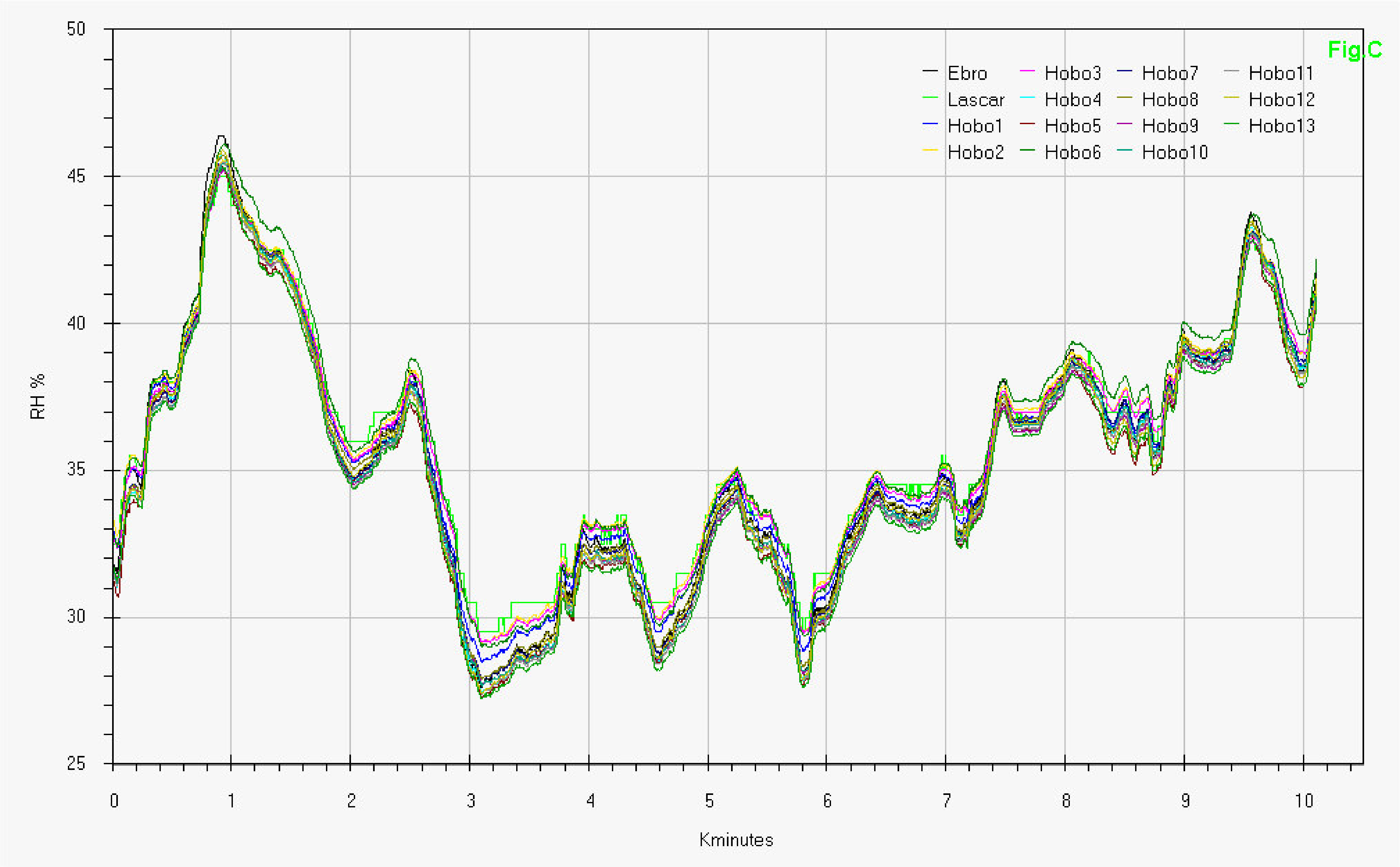
Fig.C
Ebro
Lascar
Hobo1
Hobo2
Hobo3
Hobo4
Hobo5
Hobo6
Hobo7
Hobo8
Hobo9
Hobo10
Hobo11
Hobo12
Hobo13
RH %
50
45
40
35
30
25
0
1
2
3
4
5
6
7
8
9
10
Kminutes

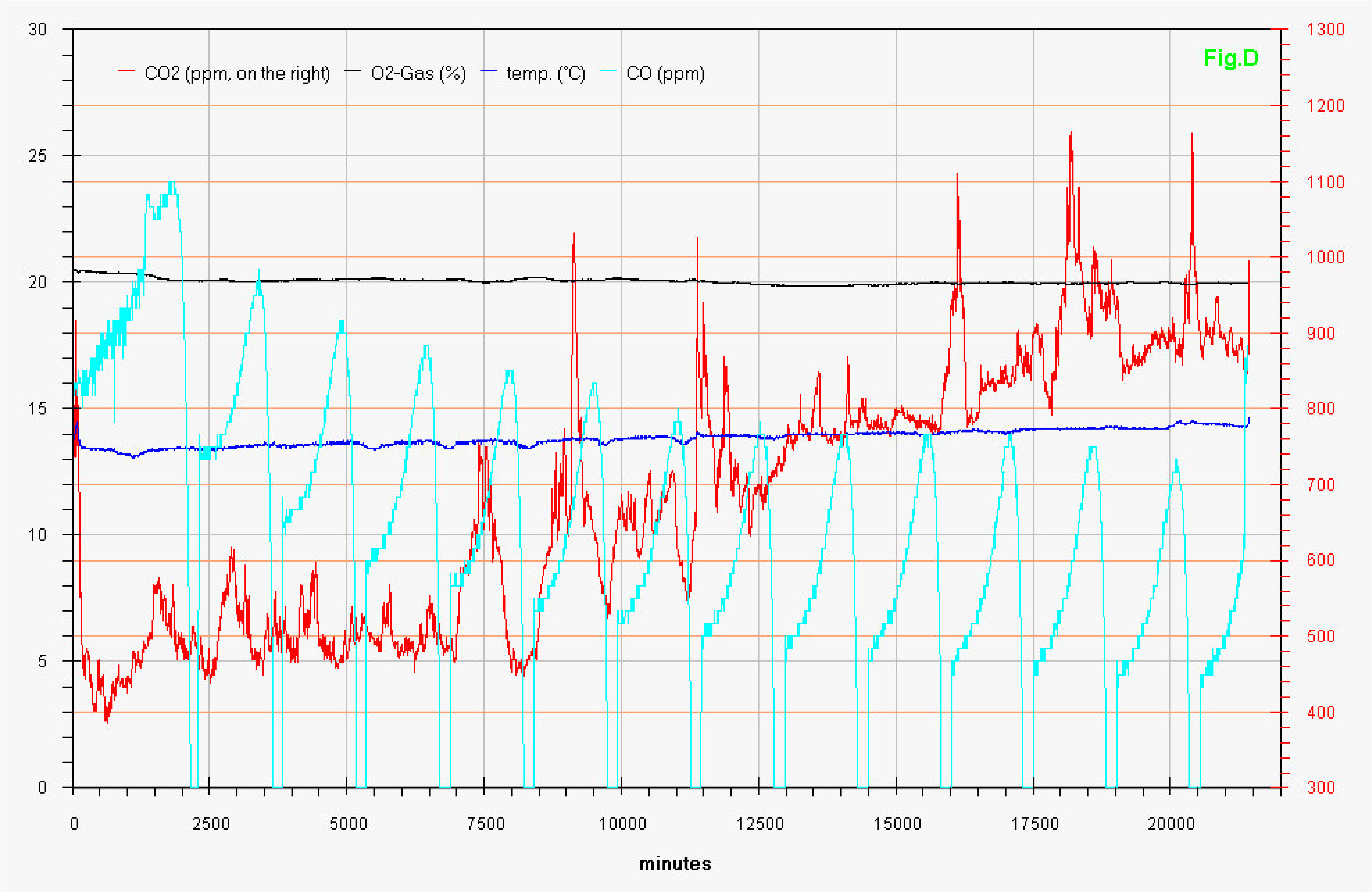
CO2 (ppm, on the right)
O2-Gas (%)
temp. (°C)
CO (ppm)
Fig.D
minutes
0
2500
5000
7500
10000
12500
15000
17500
20000
30
25
20
15
10
5
0
1300
1200
1100
1000
900
800
700
600
500
400
300

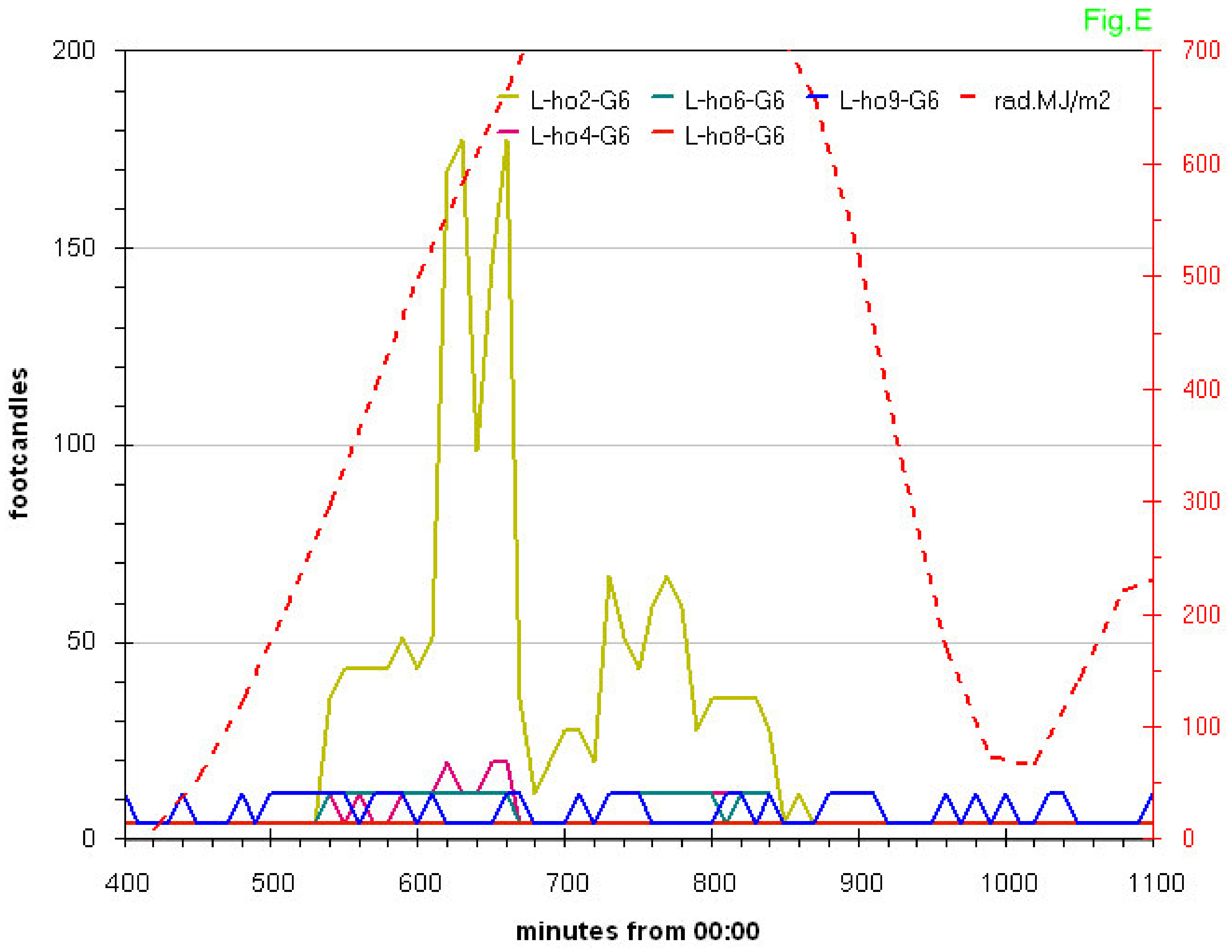
Fig.E
footcandles
minutes from 00:00
L-ho2-G6
L-ho4-G6
L-ho6-G6
L-ho8-G6
L-ho9-G6
rad.MJ/m2
0
50
100
150
200
400
500
600
700
800
900
1000
1100
0
100
200
300
400
500
600
700

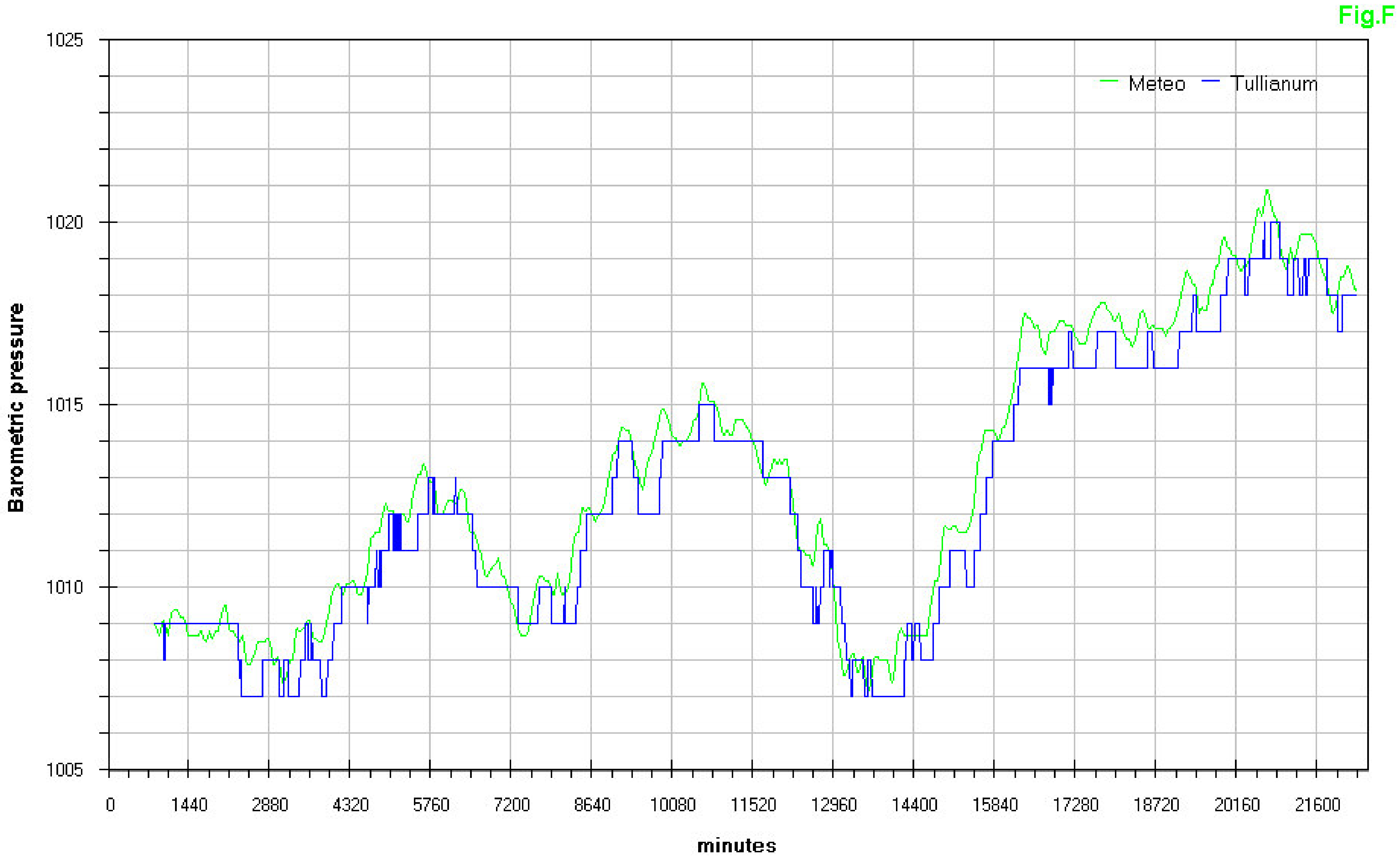
Fig.F
Barometric pressure
1025
1020
1015
1010
1005
0
1440
2880
4320
5760
7200
8640
10080
11520
12960
14400
15840
17280
18720
20160
21600
minutes
Meteo
Tullianum

Fig.Ga

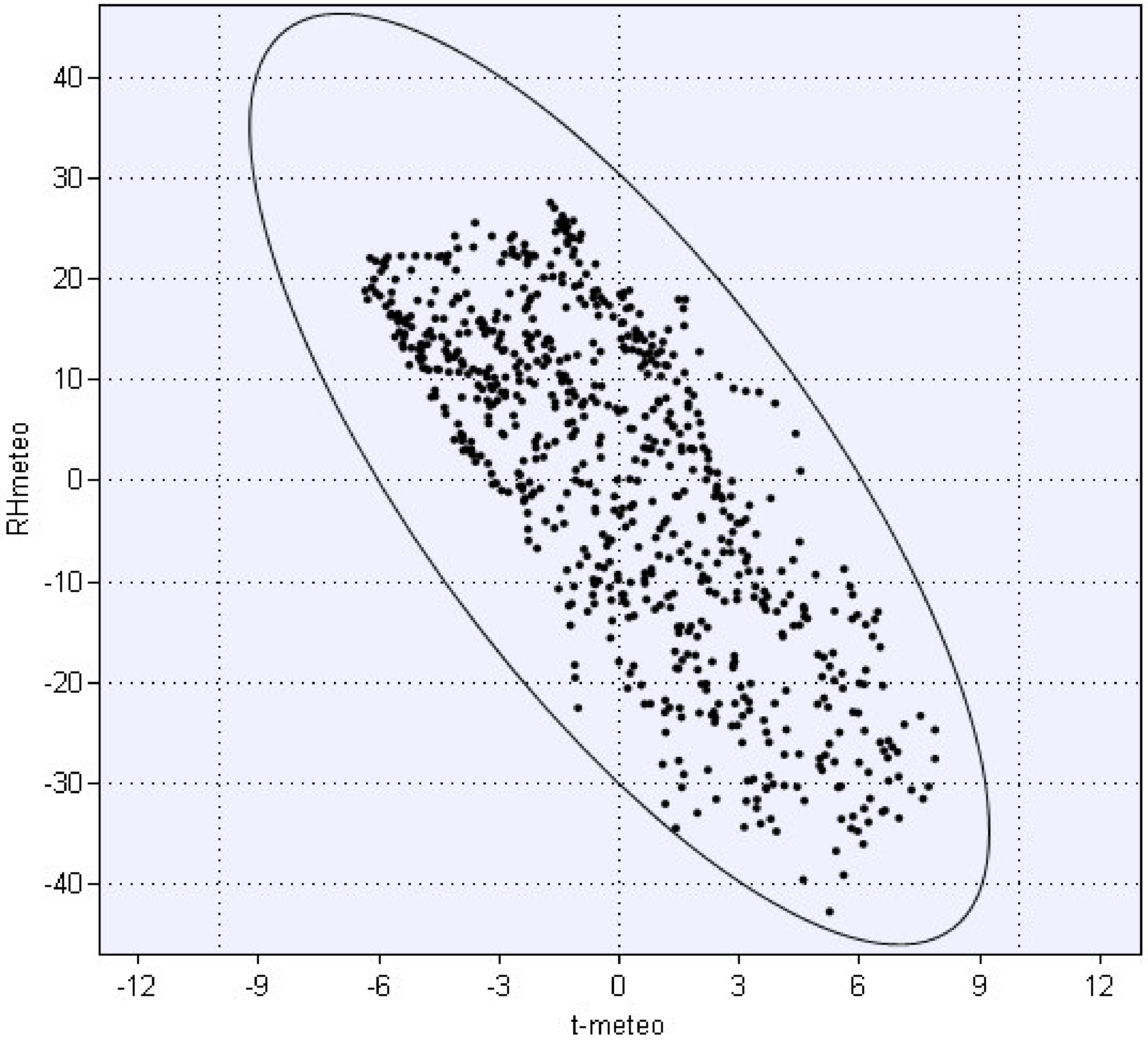

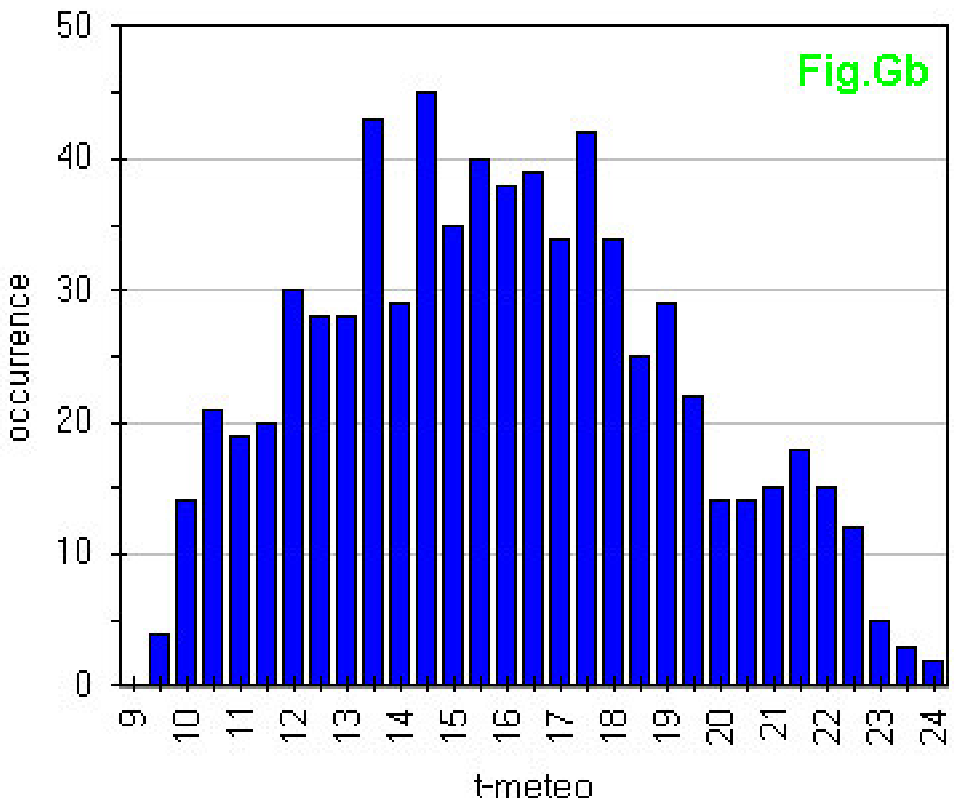
Fig.Gb
occurrence
t-meteo
0
10
20
30
40
50
9
10
11
12
13
14
15
16
17
18
19
20
21
22
23
24

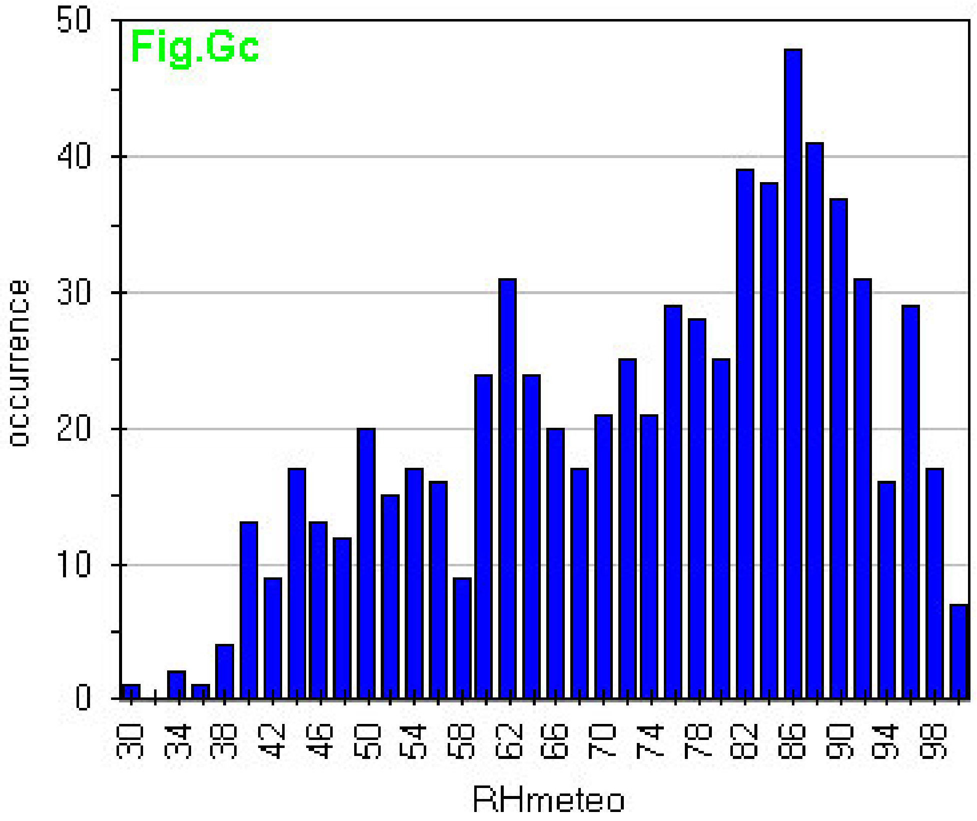

Fig.Gc
occurrence
RHmeteo
0
10
20
30
40
50
30
34
38
42
46
50
54
58
62
66
70
74
78
82
86
90
94
98

Fig.Ha

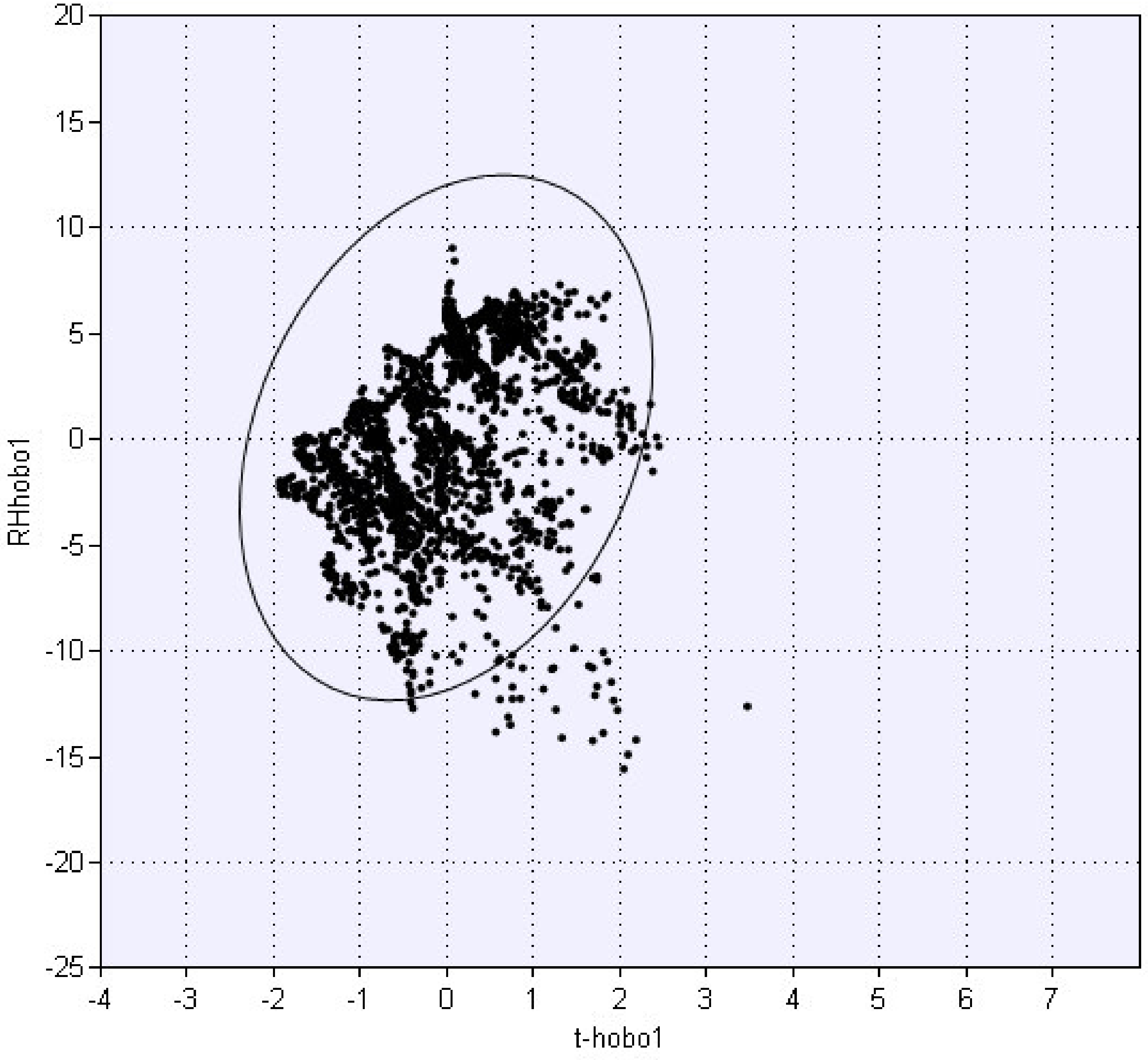

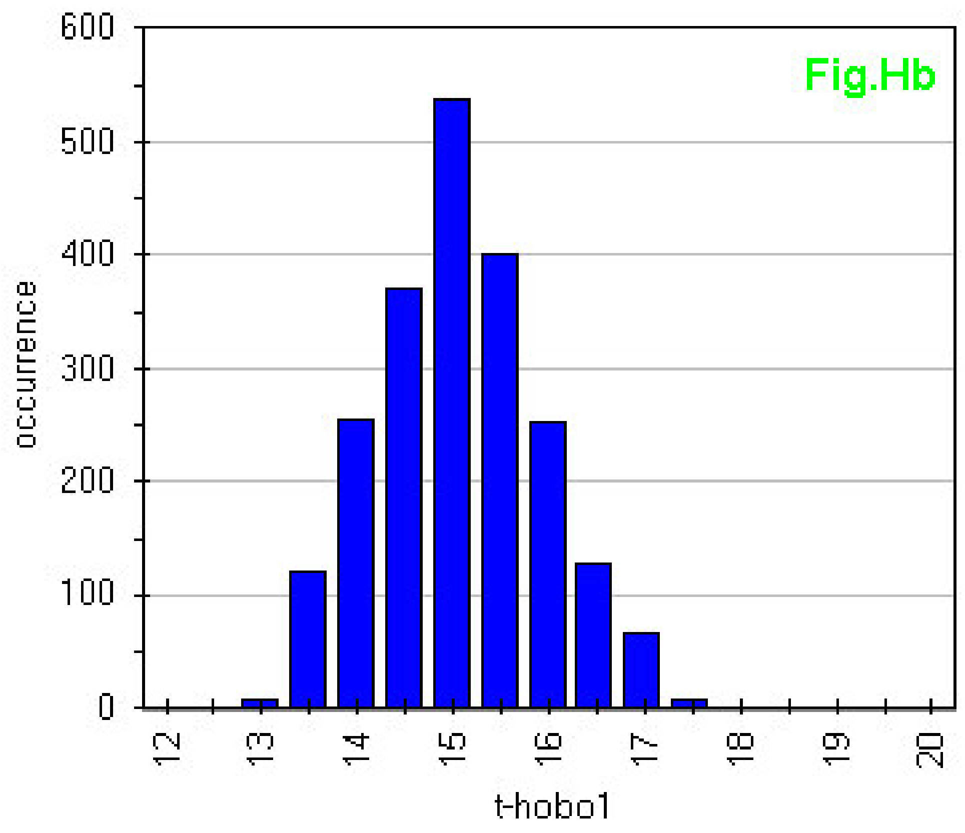

Fig.Hb
occurrence
t-hobo1
600
500
400
300
200
100
0
12
13
14
15
16
17
18
19
20

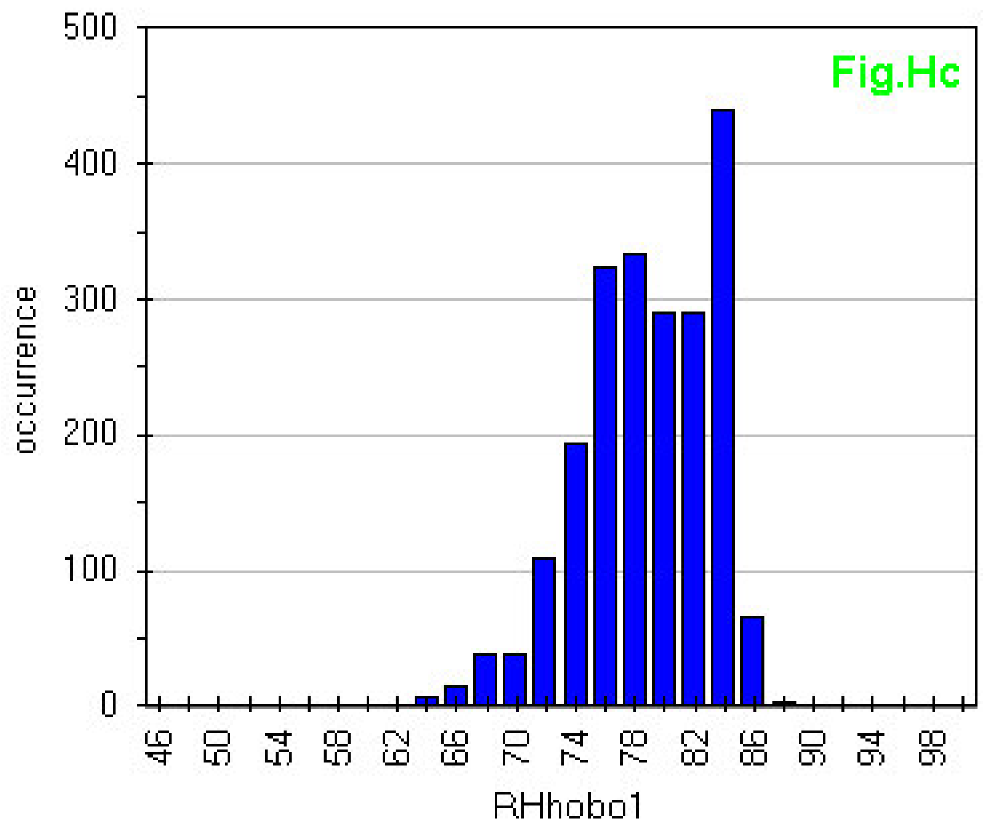

Fig.Hc
500
400
300
200
100
0
occurrence
46
50
54
58
62
66
70
74
78
82
86
90
94
98
RHhobo1

Fig.la

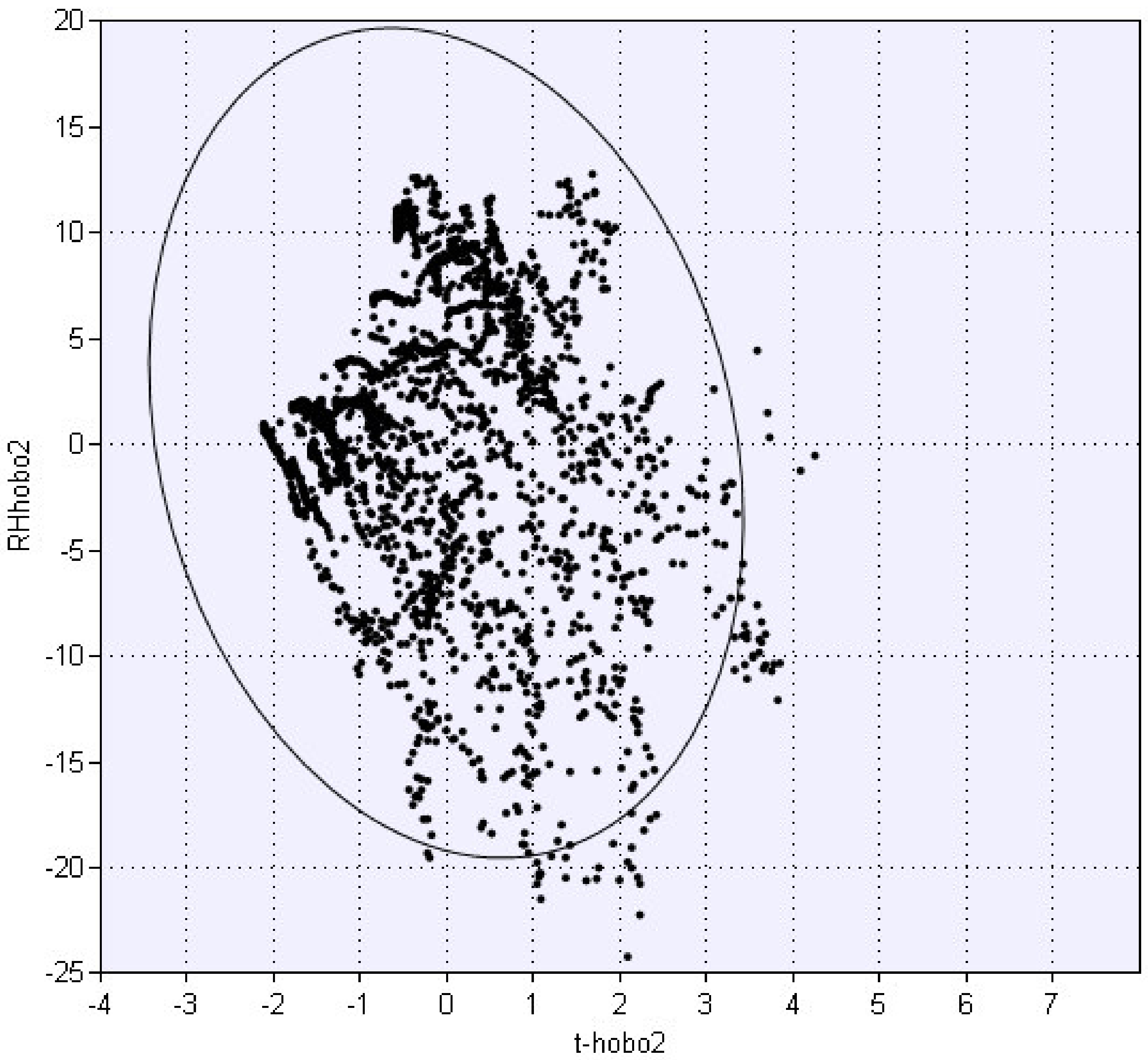

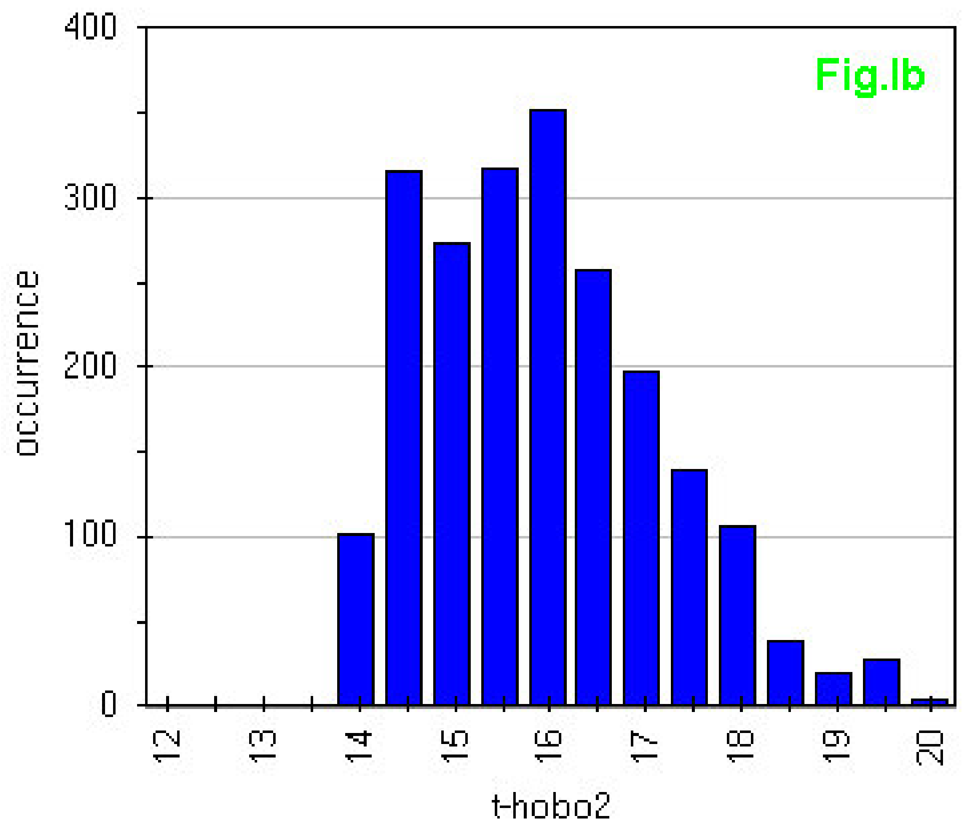

Fig.Ib
400
300
200
100
0
occurrence
12
13
14
15
16
17
18
19
20
t-hobo2

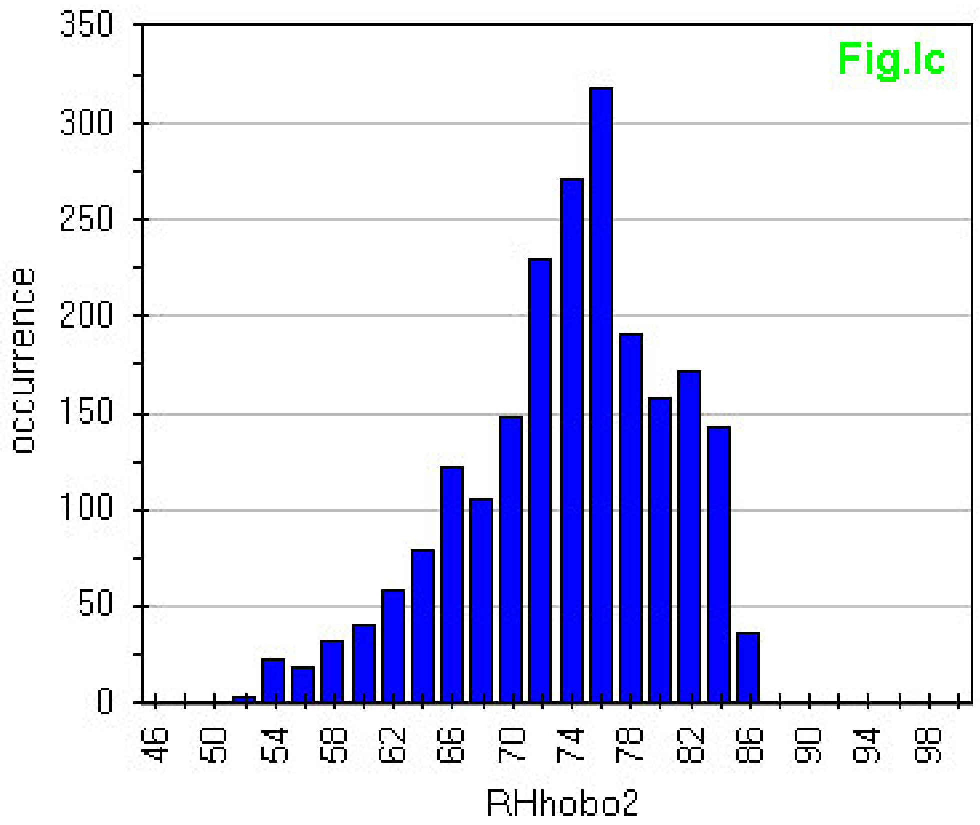
Fig.lc
occurrence
RHhobo2
350
300
250
200
150
100
50
0
46
50
54
58
62
66
70
74
78
82
86
90
94
98

Fig.Ja

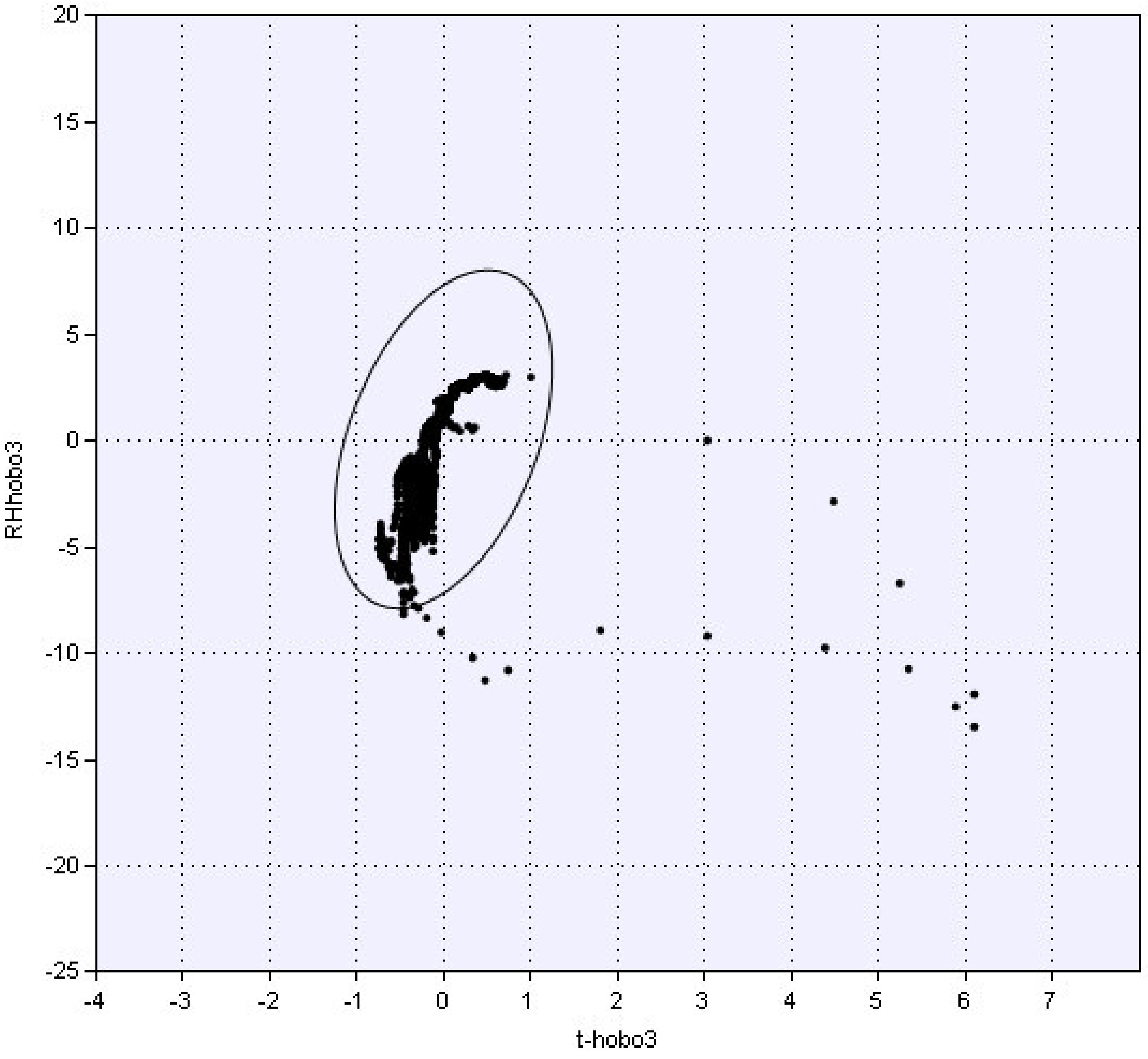

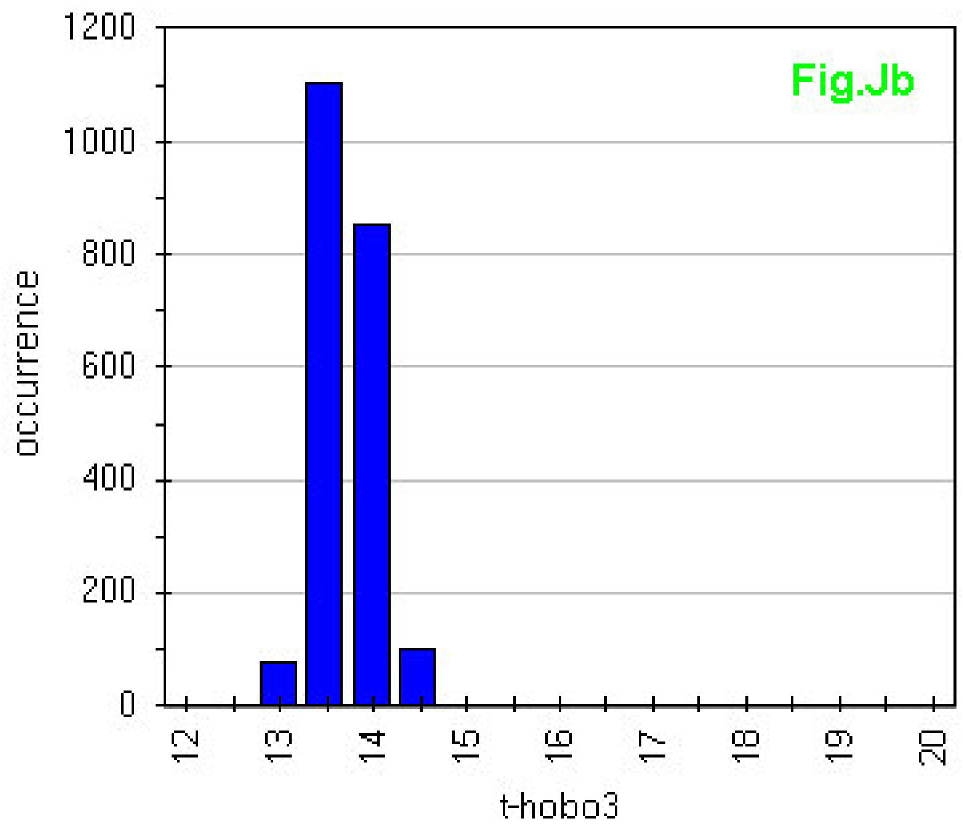
Fig.Jb
1200
1000
800
600
400
200
0
occurrence
12
13
14
15
16
17
18
19
20
t-hobo3

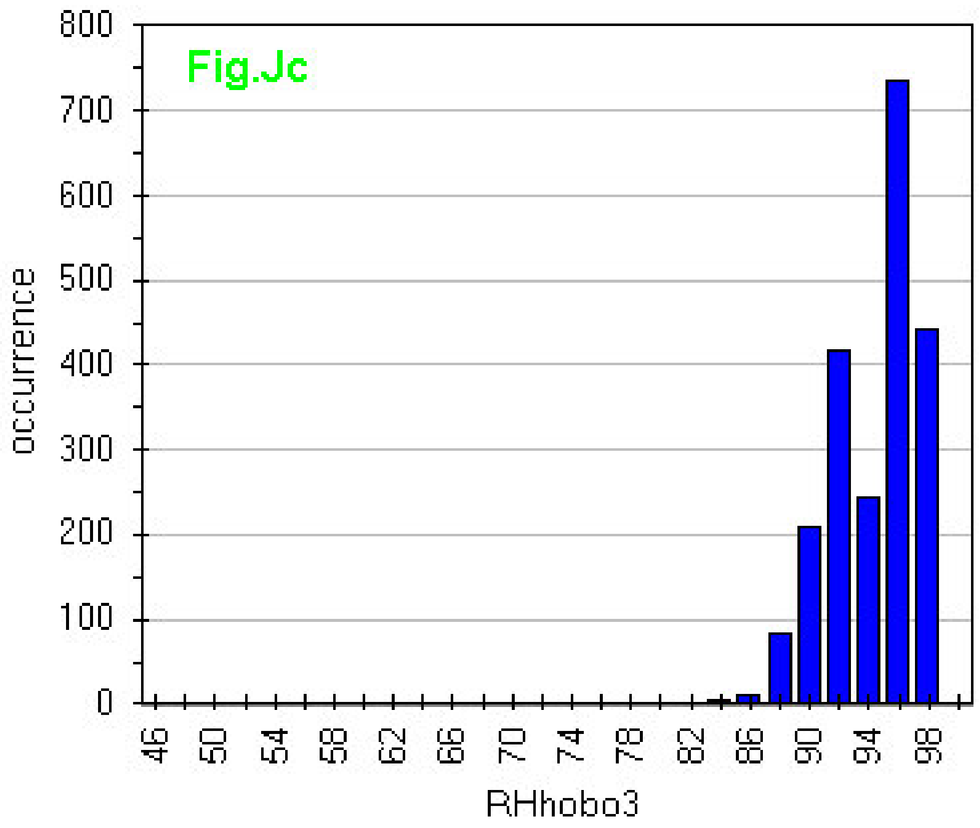
Fig.Jc
occurrence
800
700
600
500
400
300
200
100
0
46
50
54
58
62
66
70
74
78
82
86
90
94
98
RHhobo3

Fig.Ka

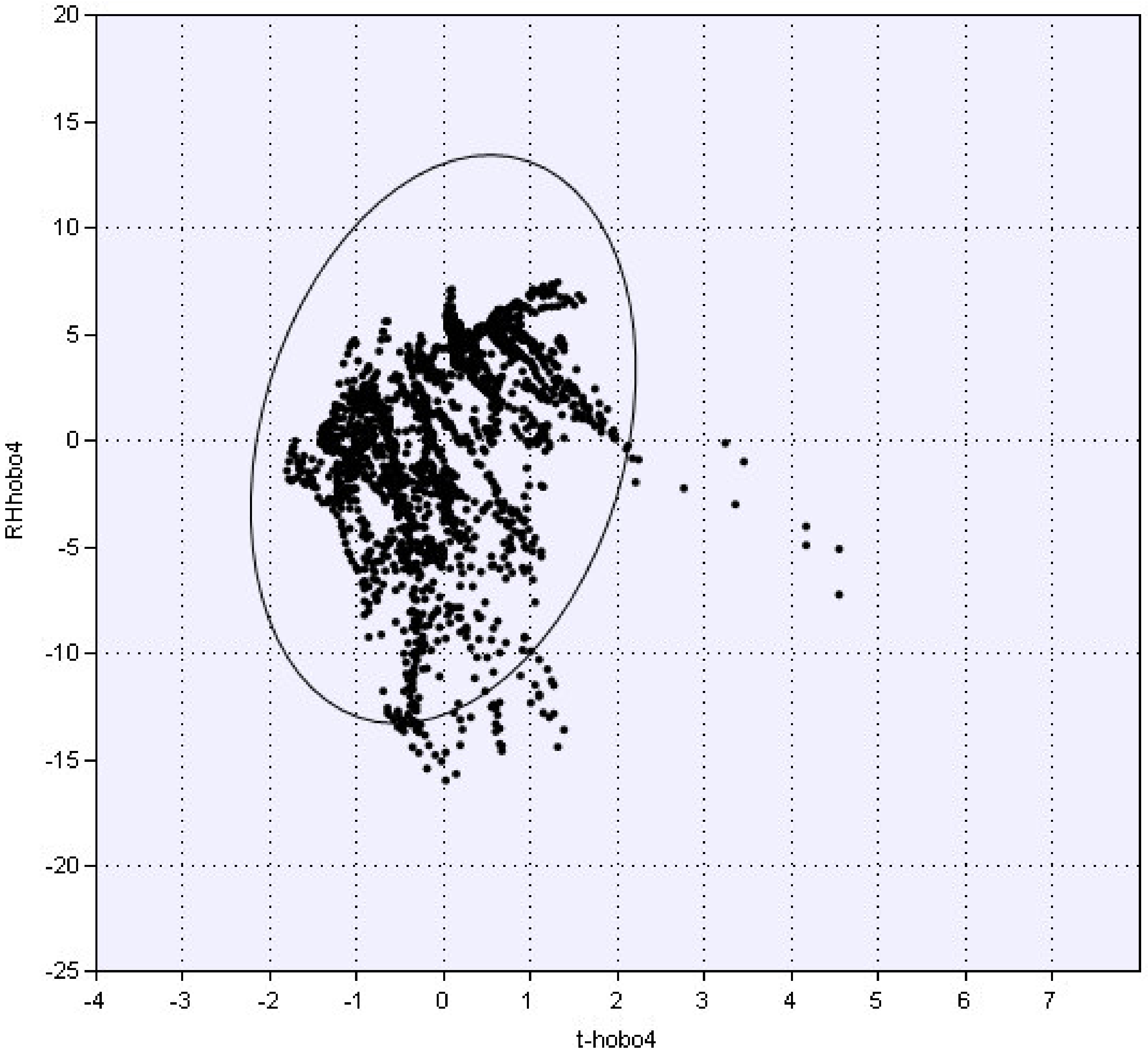

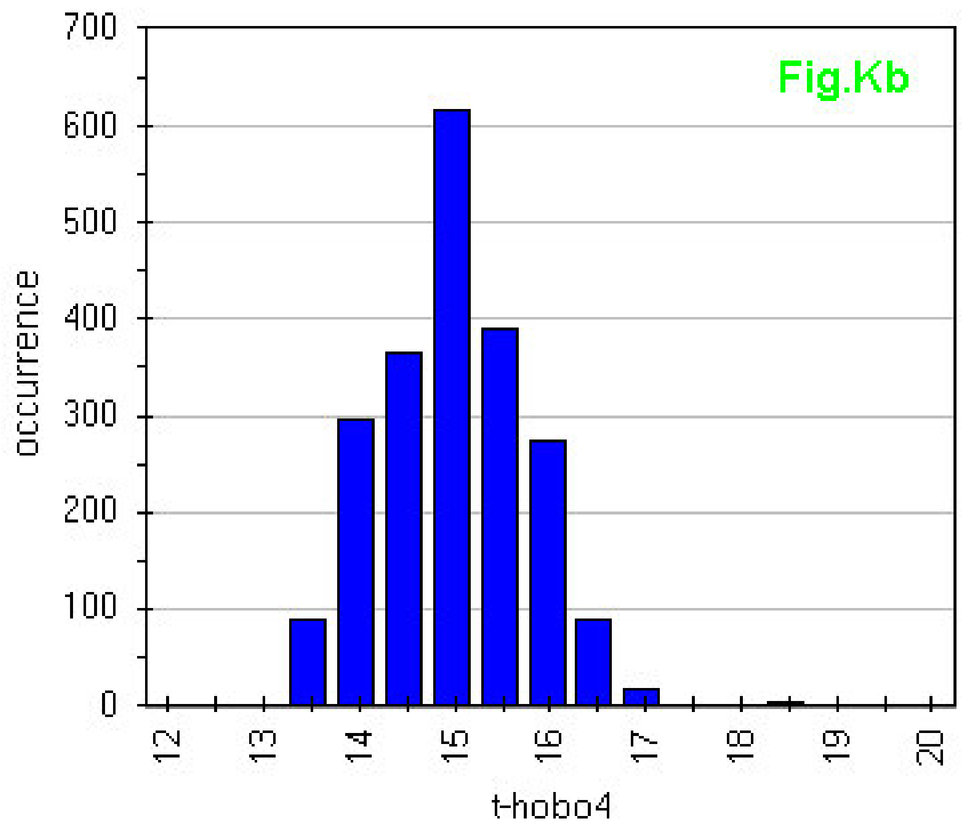
Fig.Kb
occurrence
t-hobo4
0
100
200
300
400
500
600
700
12
13
14
15
16
17
18
19
20

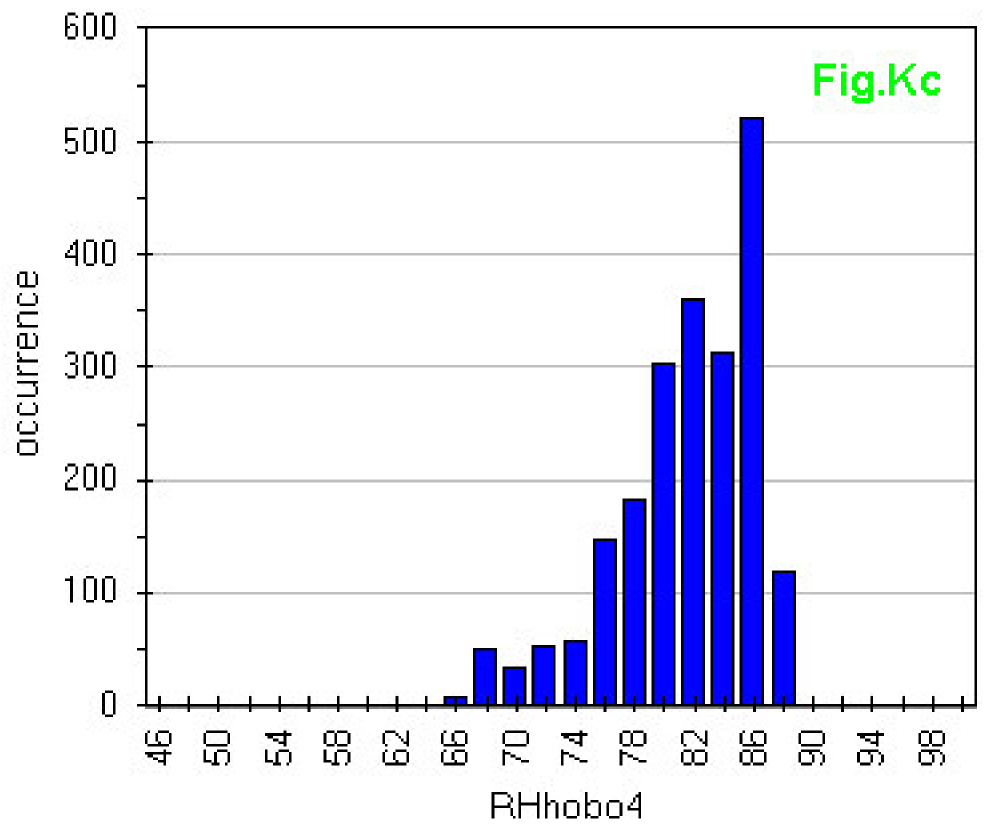

Fig.Kc
600
500
400
300
200
100
0
occurrence
46
50
54
58
62
66
70
74
78
82
86
90
94
98
RHhobo4

Fig.La

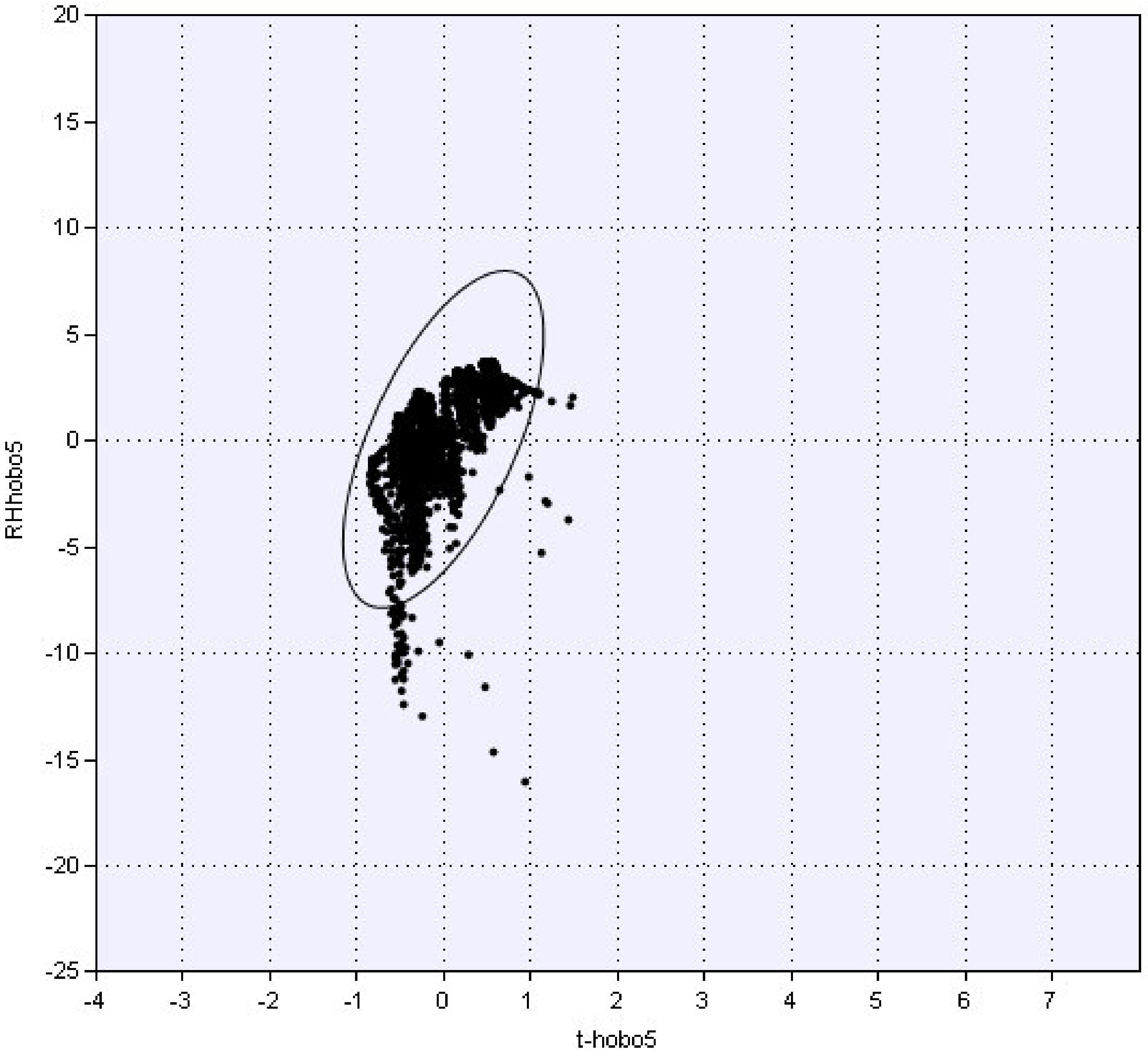

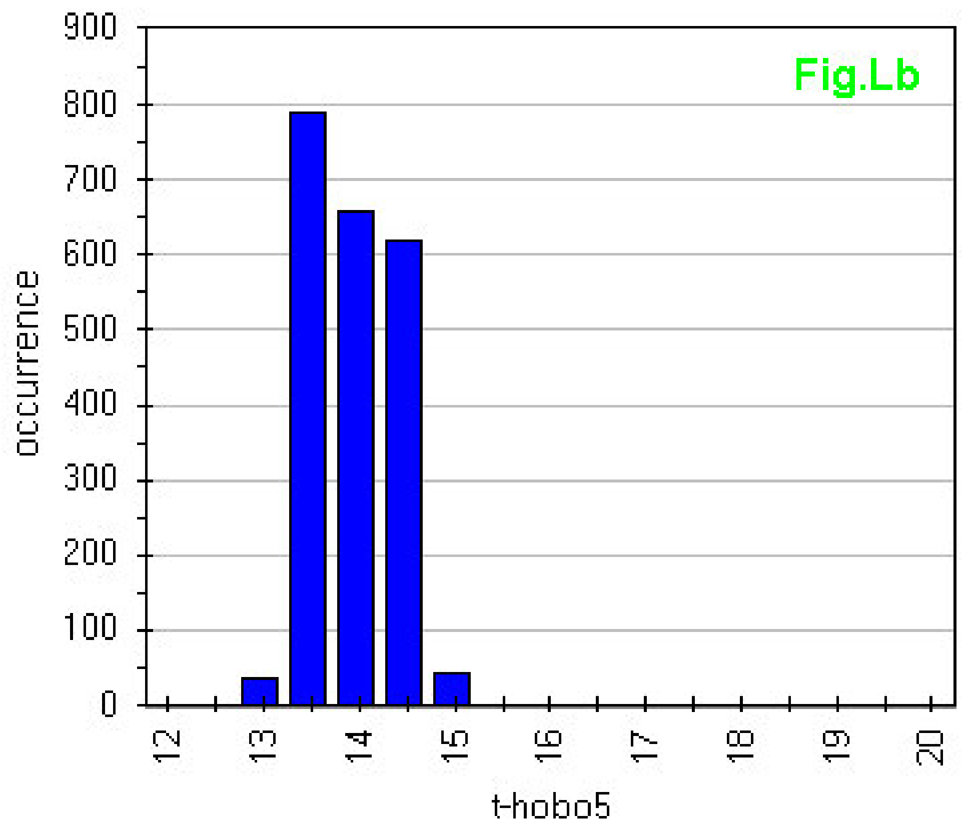
Fig.Lb
occurrence
0
100
200
300
400
500
600
700
800
900
12
13
14
15
16
17
18
19
20
t-hobo5

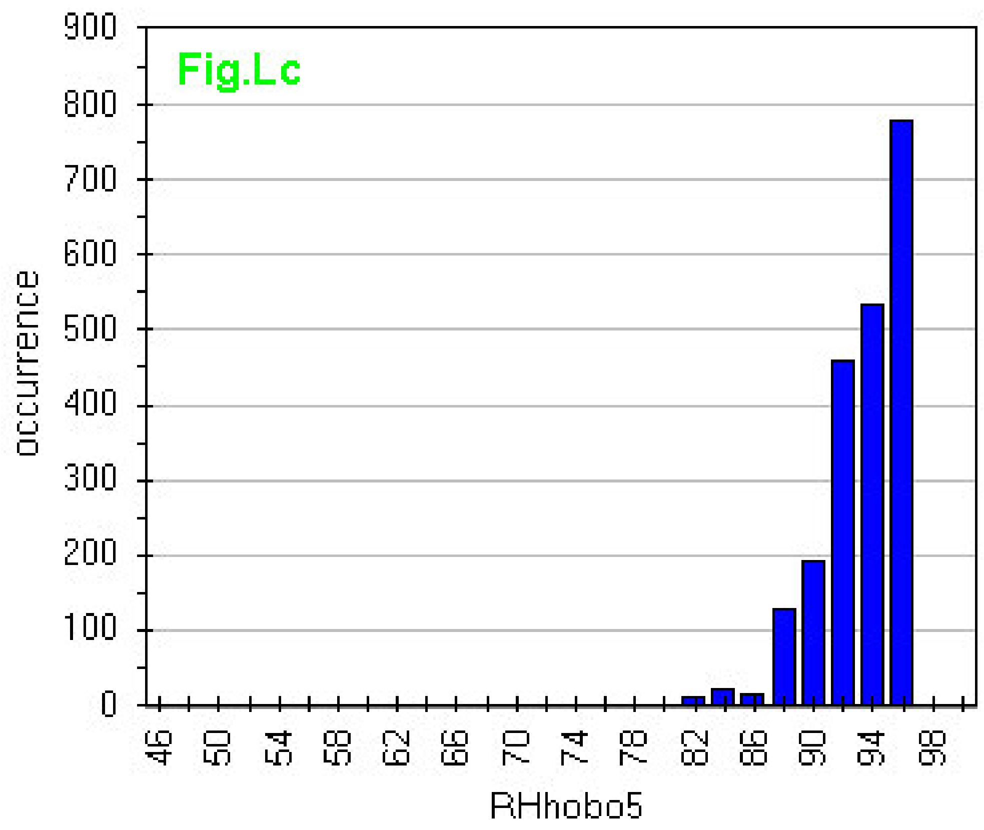

Fig.Lc
occurrence
900
800
700
600
500
400
300
200
100
0
46
50
54
58
62
66
70
74
78
82
86
90
94
98
RHhobo5

Fig.Ma

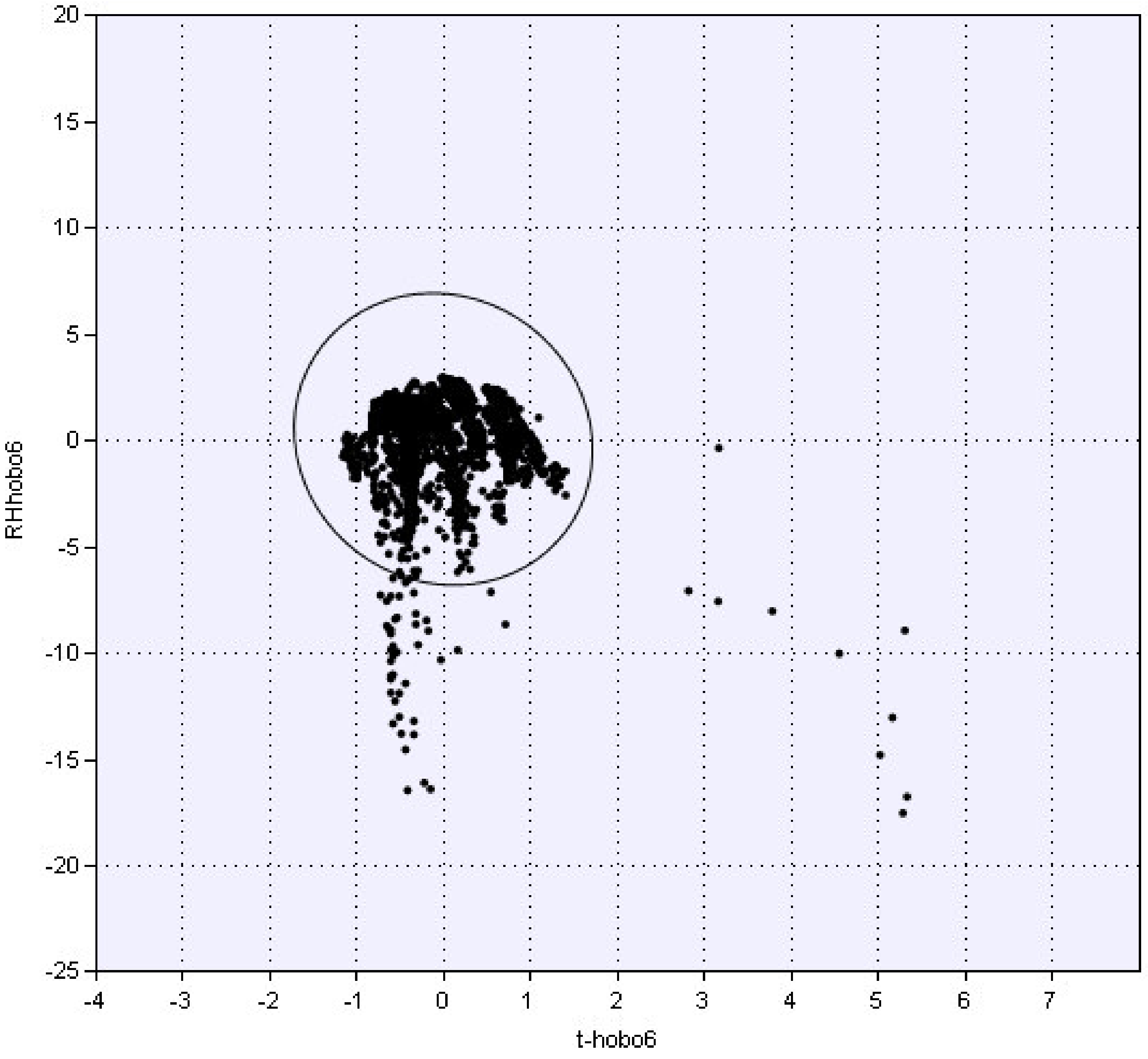

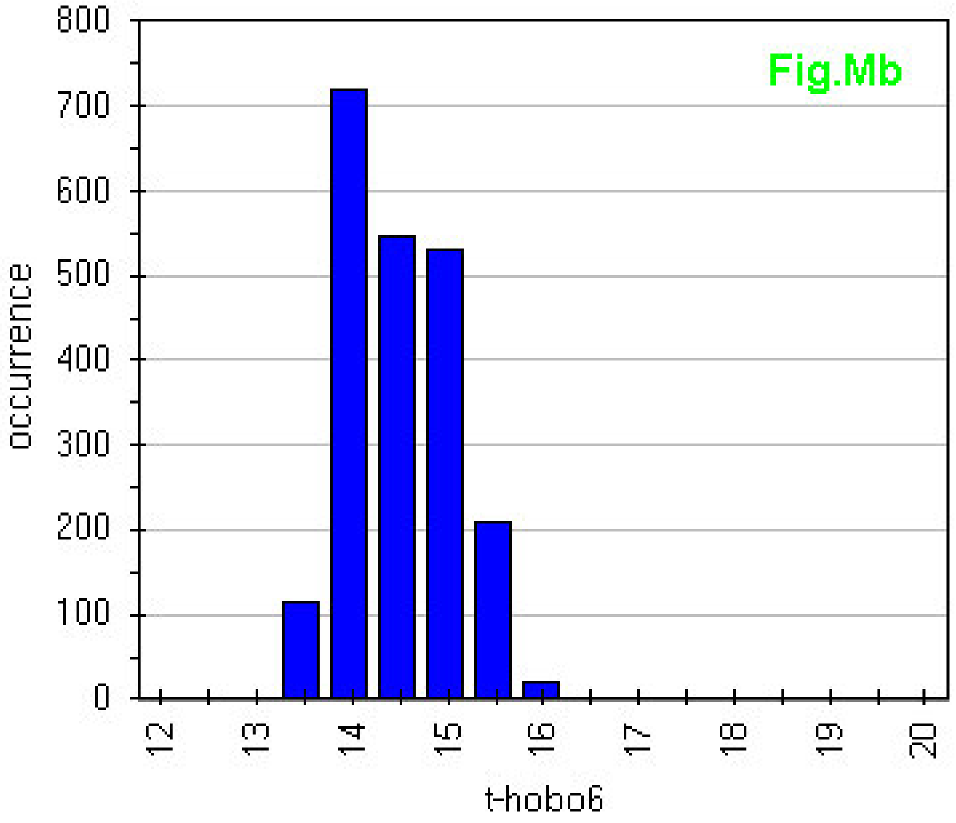
Fig.Mb
800
700
600
500
400
300
200
100
0
occurrence
12
13
14
15
16
17
18
19
20
t-hobo6

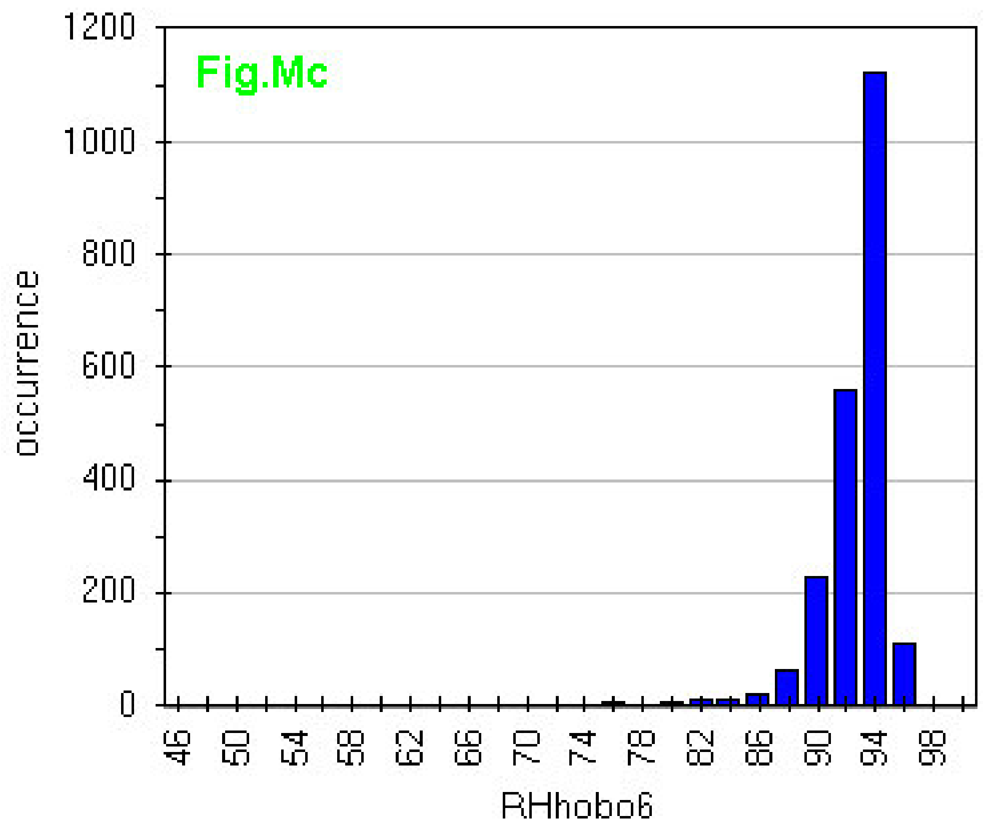
Fig.Mc
1200
1000
800
600
400
200
0
occurrence
46
50
54
58
62
66
70
74
78
82
86
90
94
98
RHhobo6

Fig.Na

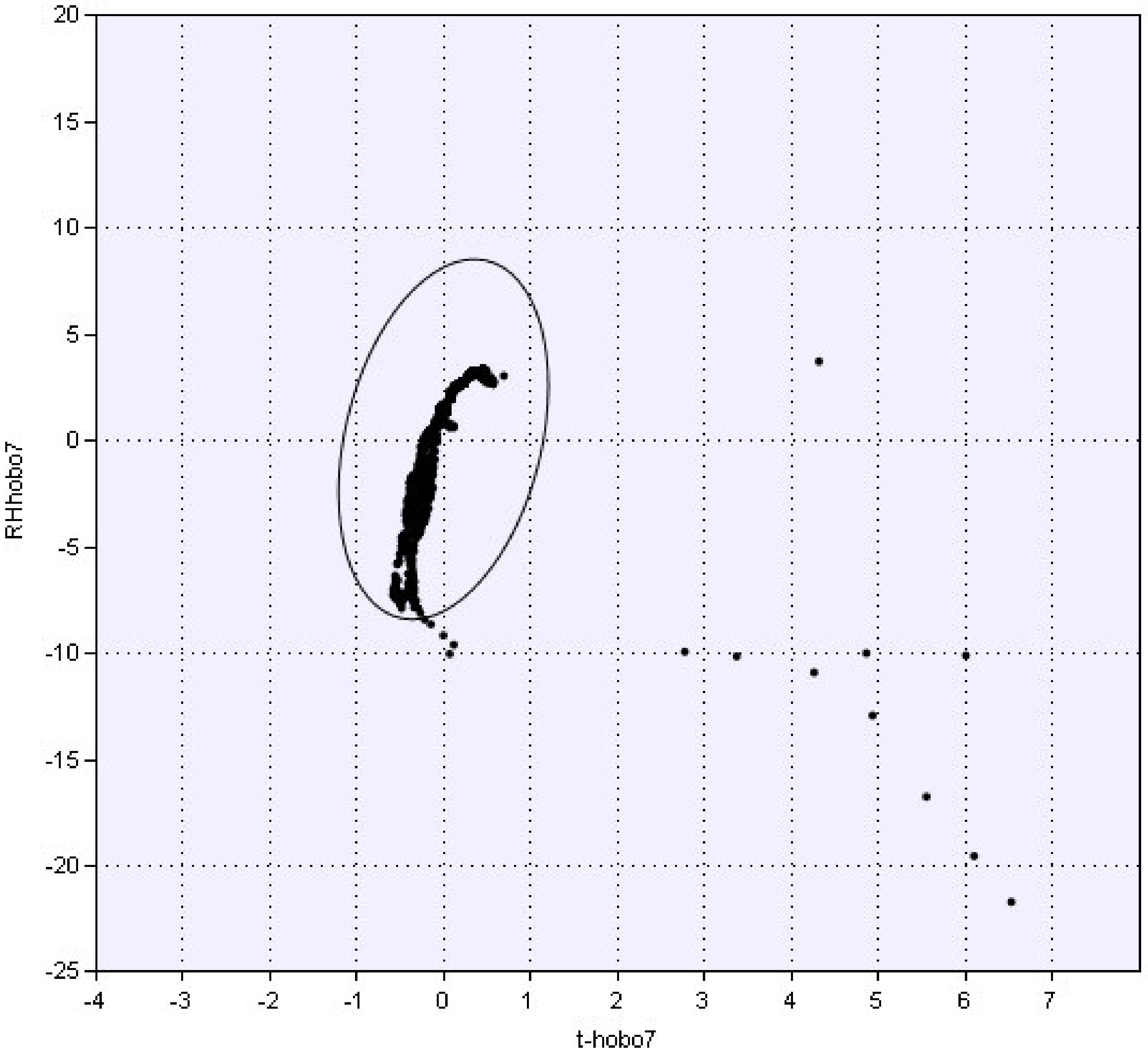

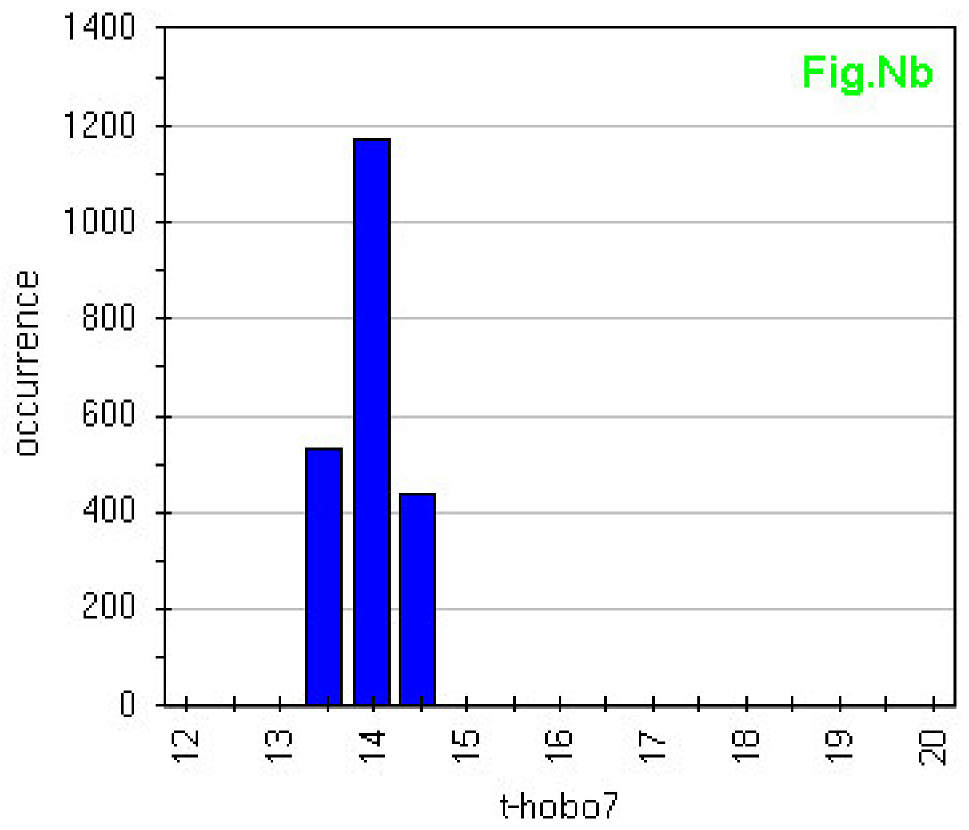
Fig.Nb
1400
1200
1000
800
600
400
200
0
occurrence
12
13
14
15
16
17
18
19
20
t-hobo7

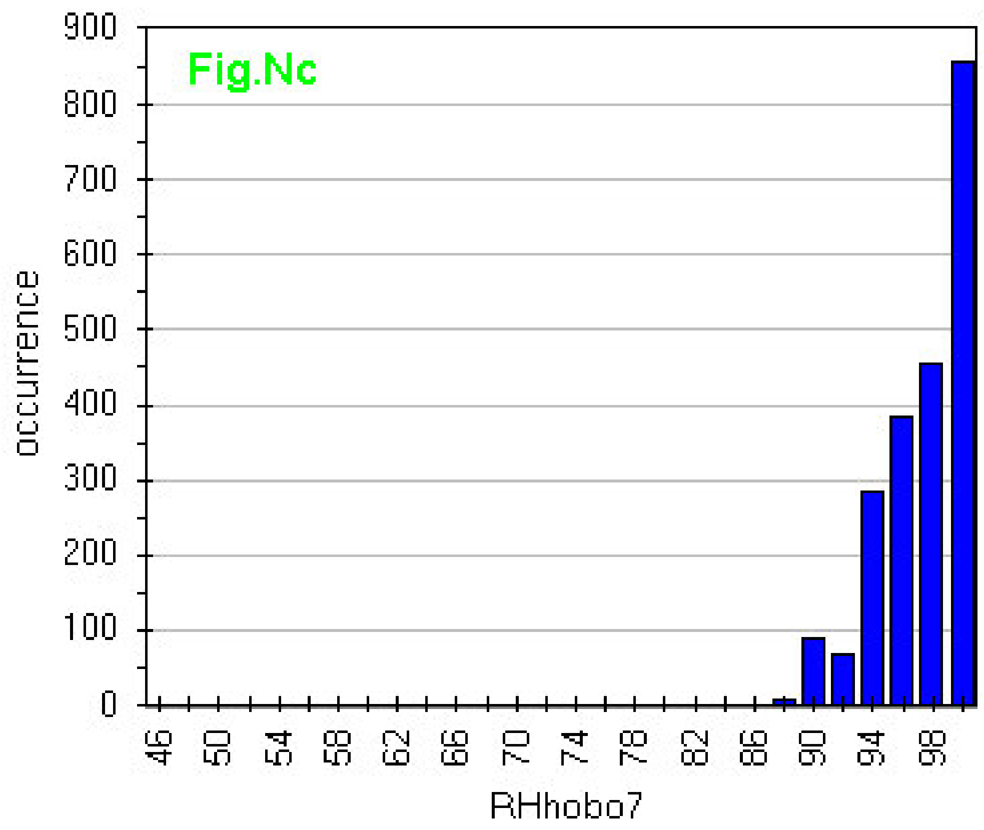
Fig.Nc
occurrence
RHhobo7
0
100
200
300
400
500
600
700
800
900
46
50
54
58
62
66
70
74
78
82
86
90
94
98

Fig.Oa

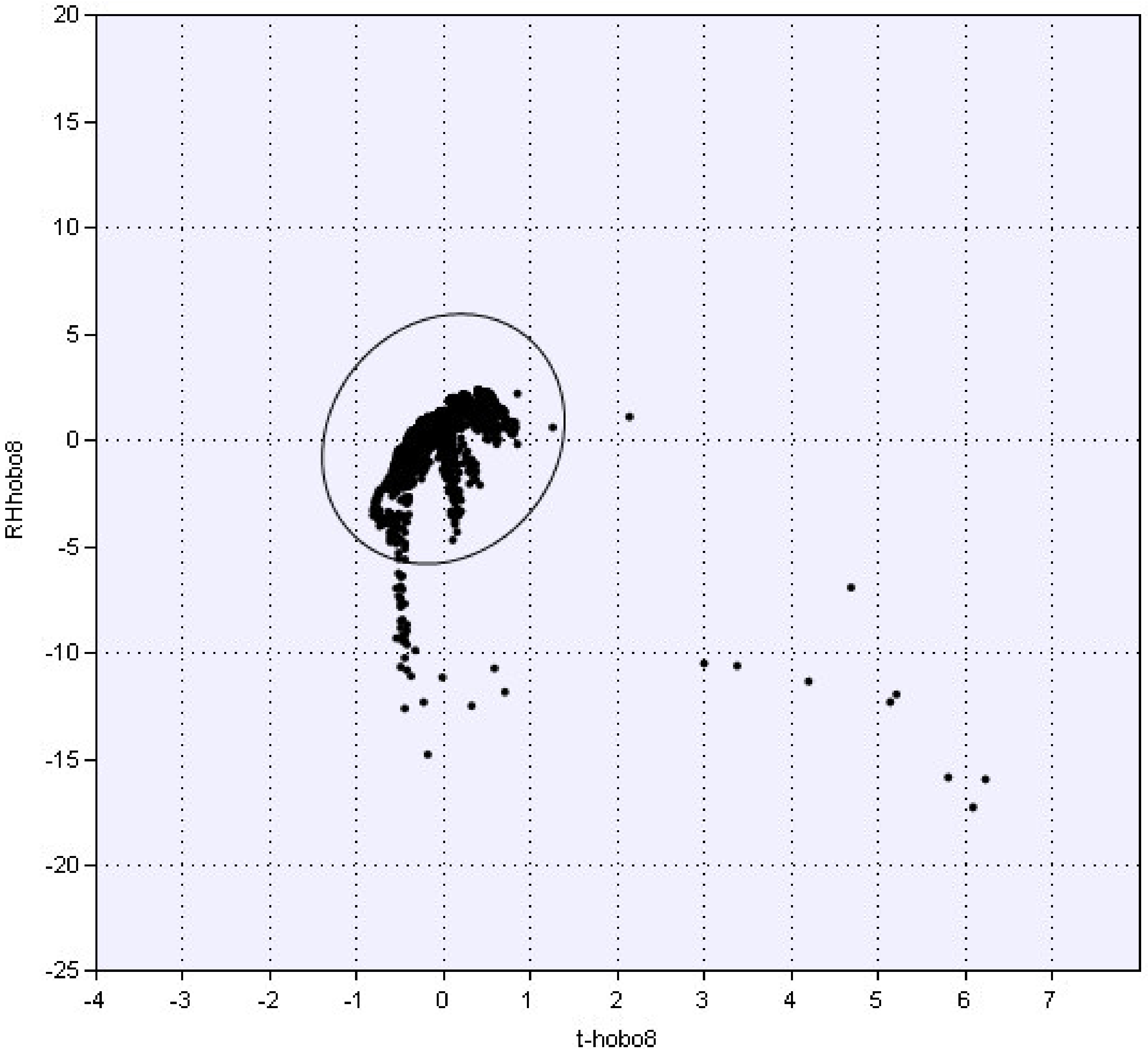

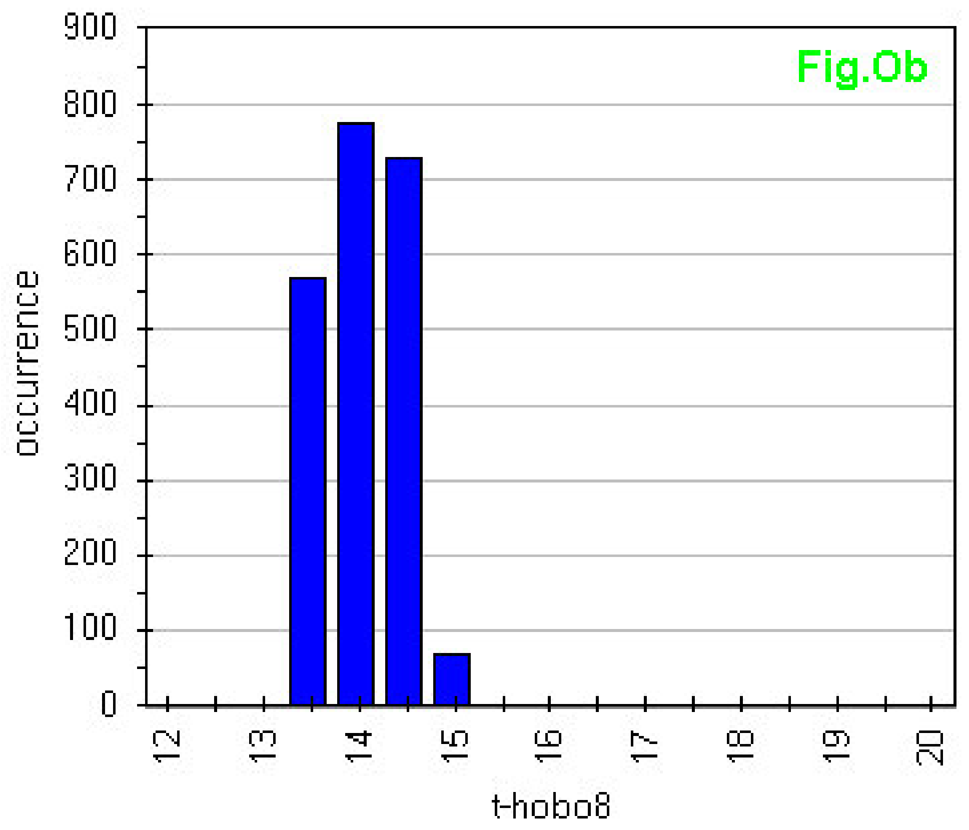

Fig.Ob
occurrence
t-hobo8
0
100
200
300
400
500
600
700
800
900
12
13
14
15
16
17
18
19
20

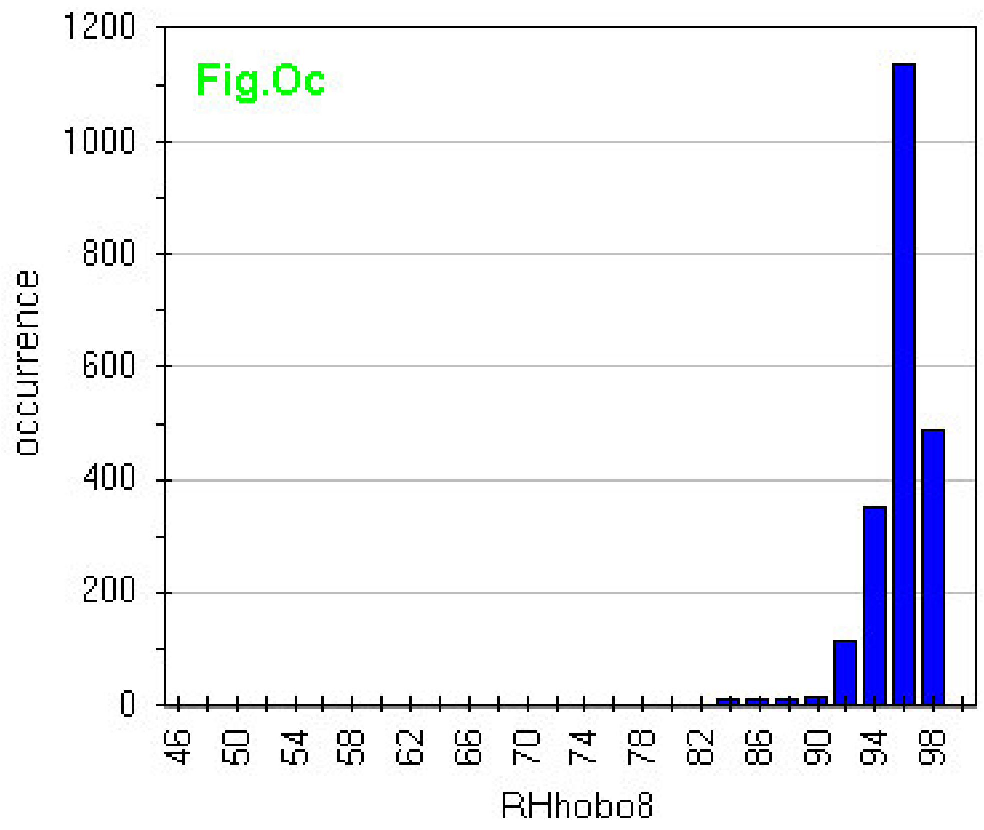
Fig.Oc
1200
1000
800
600
400
200
0
occurrence
46
50
54
58
62
66
70
74
78
82
86
90
94
98
RHhobo8

Fig.Pa

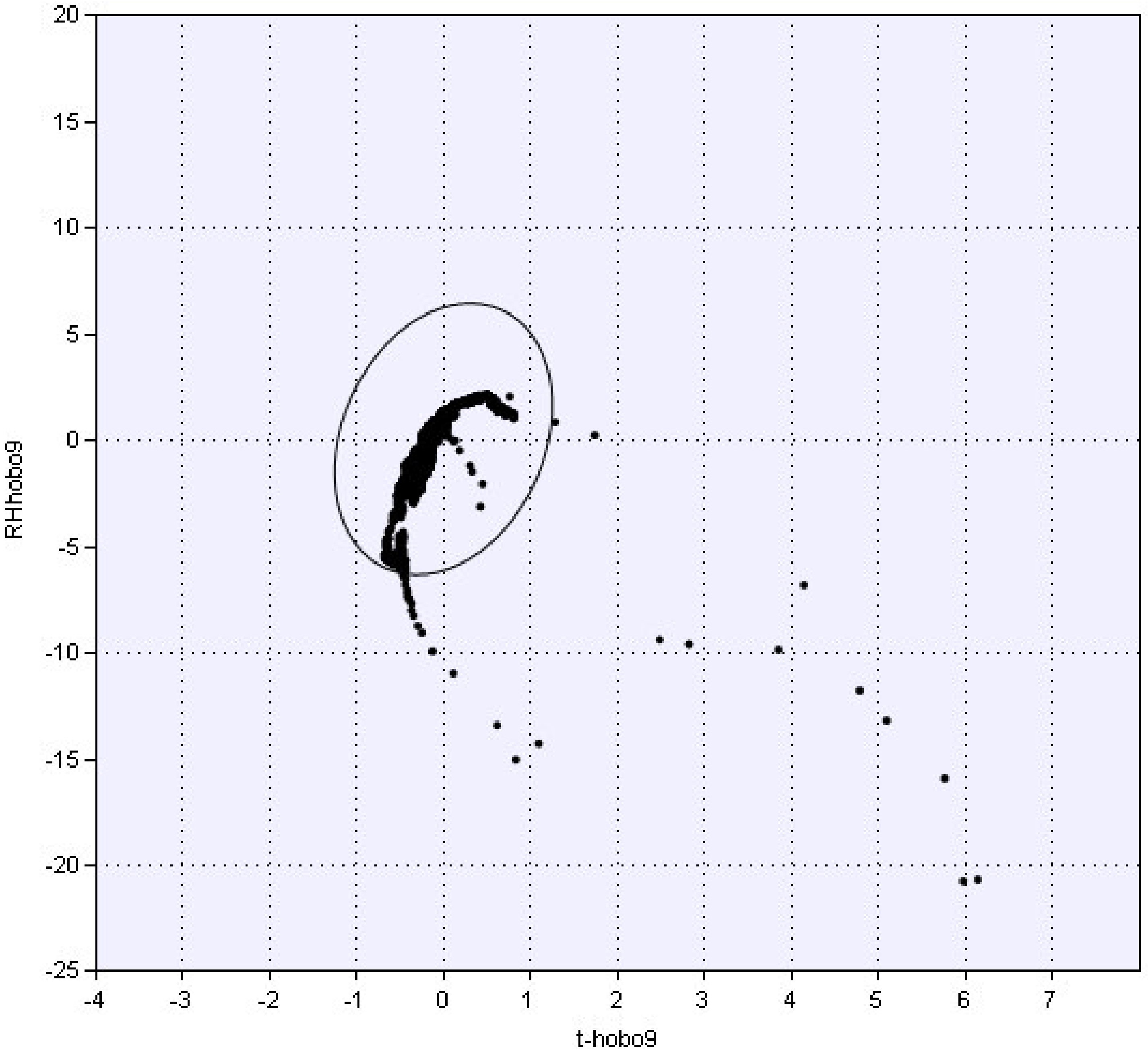

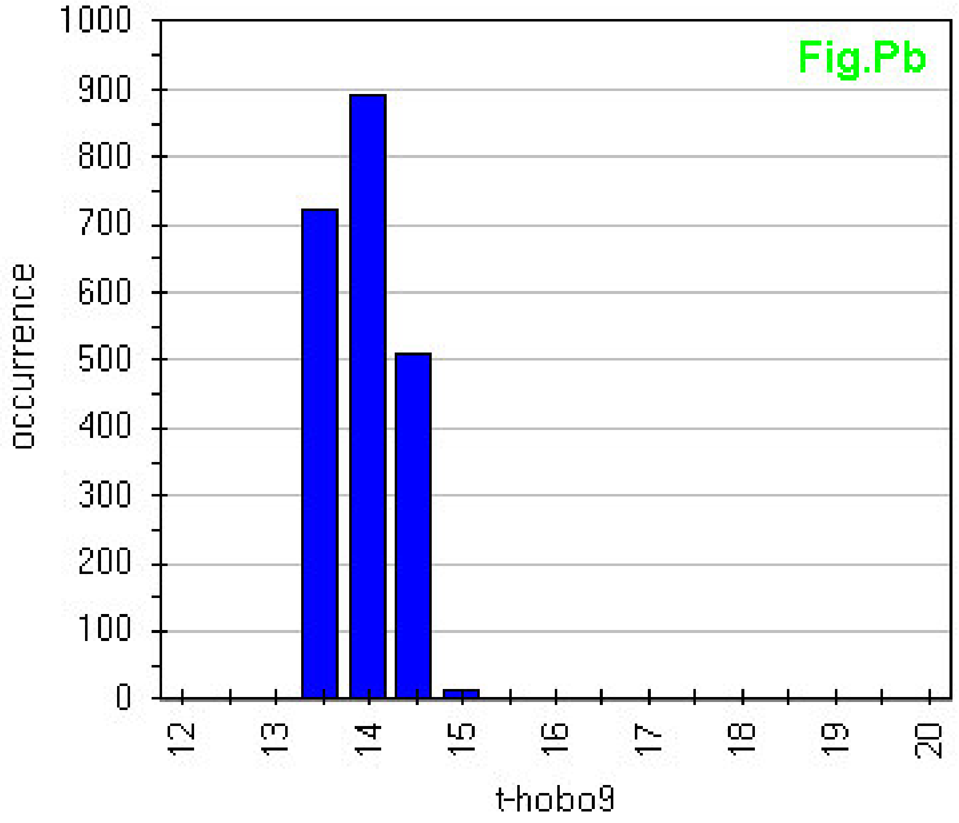
Fig.Pb
occurrence
1000
900
800
700
600
500
400
300
200
100
0
12
13
14
15
16
17
18
19
20
t-hobo9

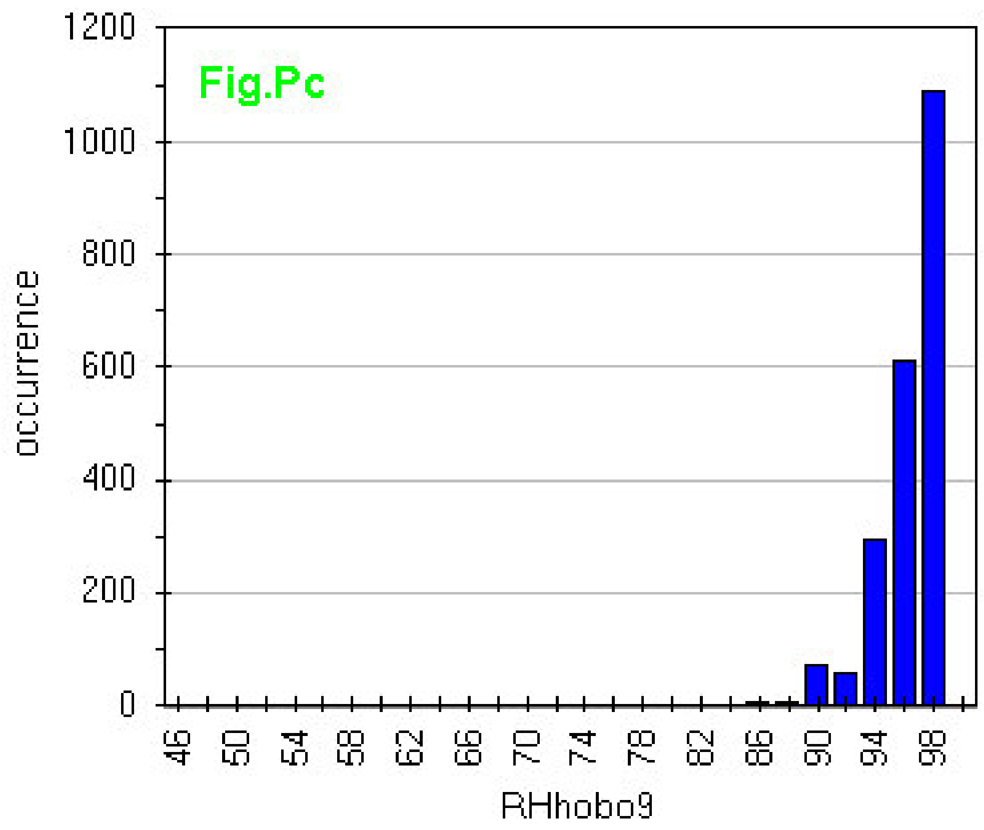
Fig.Pc
1200
1000
800
600
400
200
0
occurrence
46
50
54
58
62
66
70
74
78
82
86
90
94
98
RHhobo9

Fig.Qa

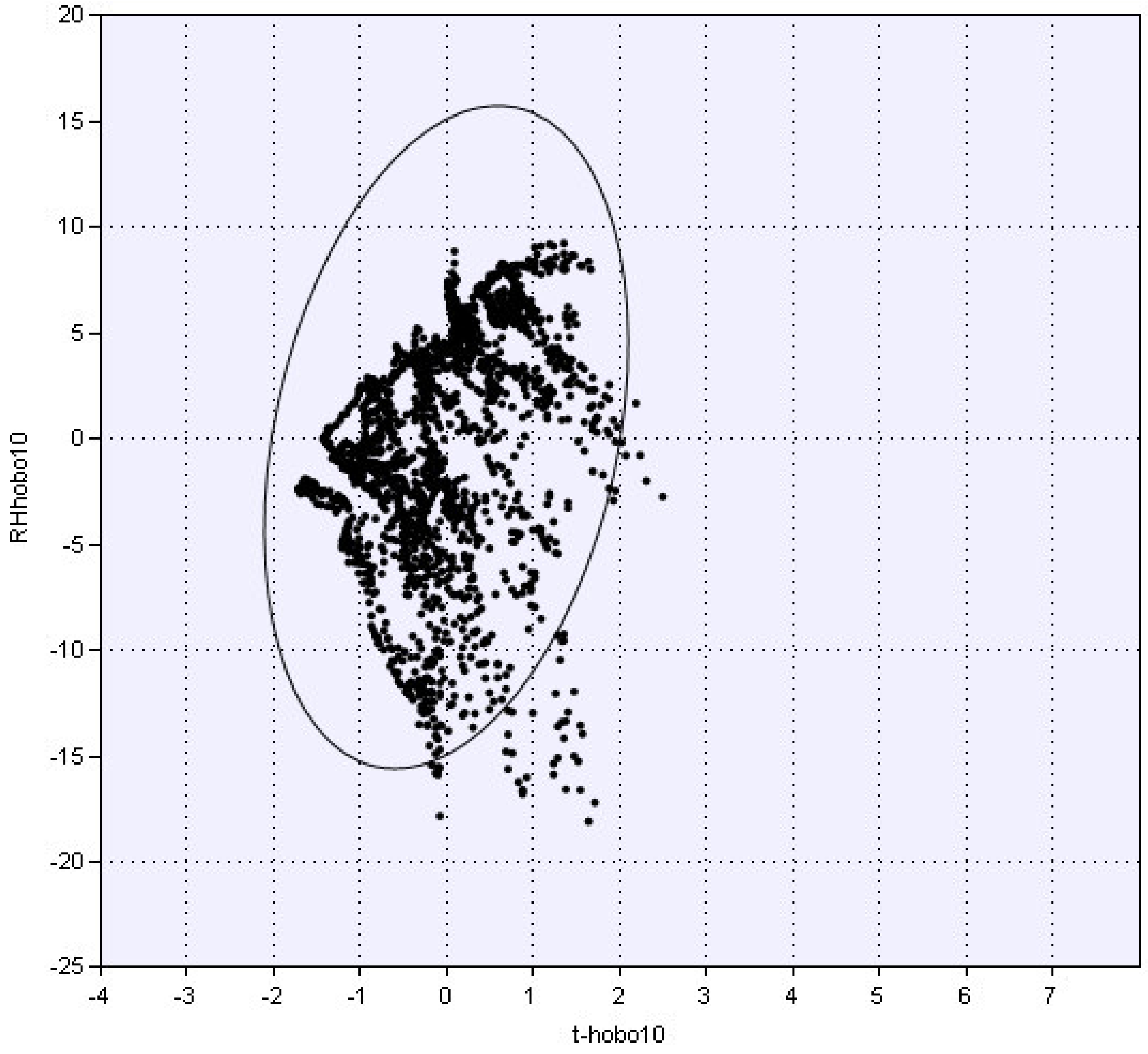

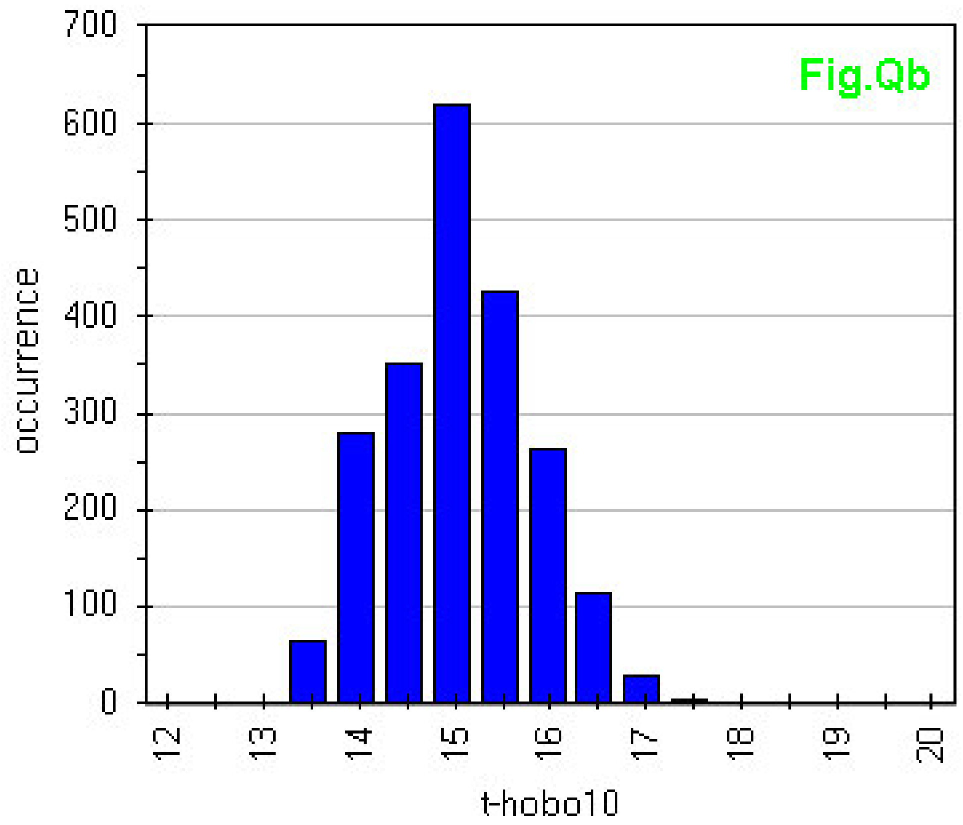

Fig.Qb
occurrence
t-hobo10

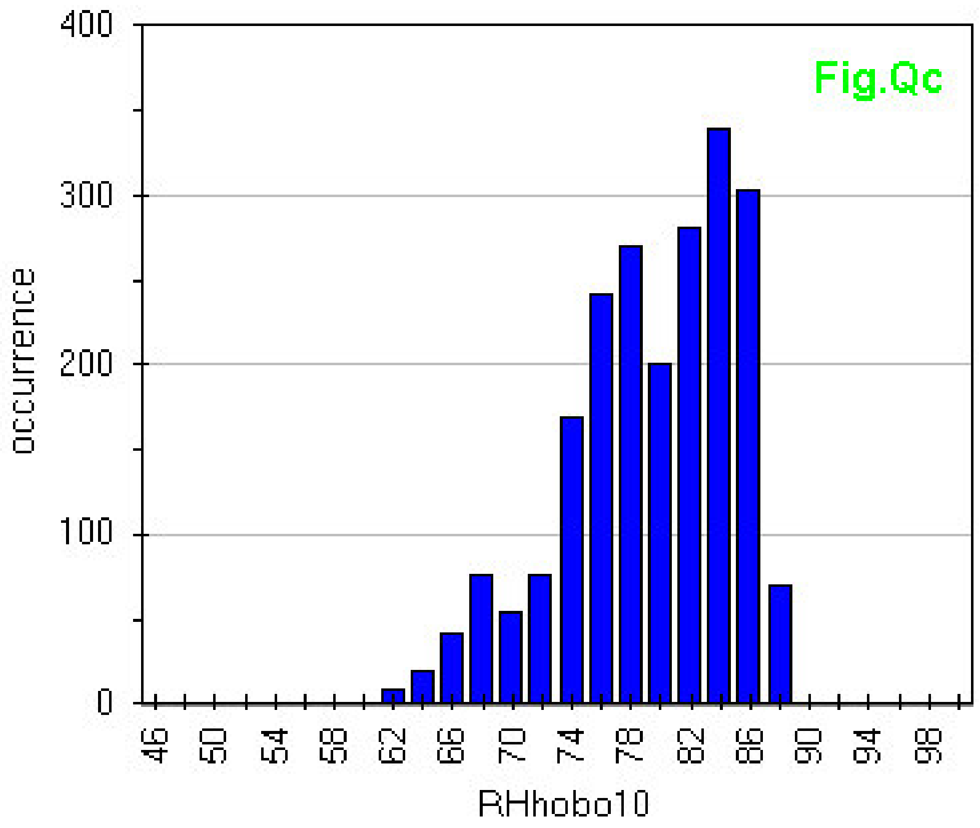

Fig.Qc
400
300
200
100
0
occurrence
46
50
54
58
62
66
70
74
78
82
86
90
94
98
RHhobo10

Fig.Ra

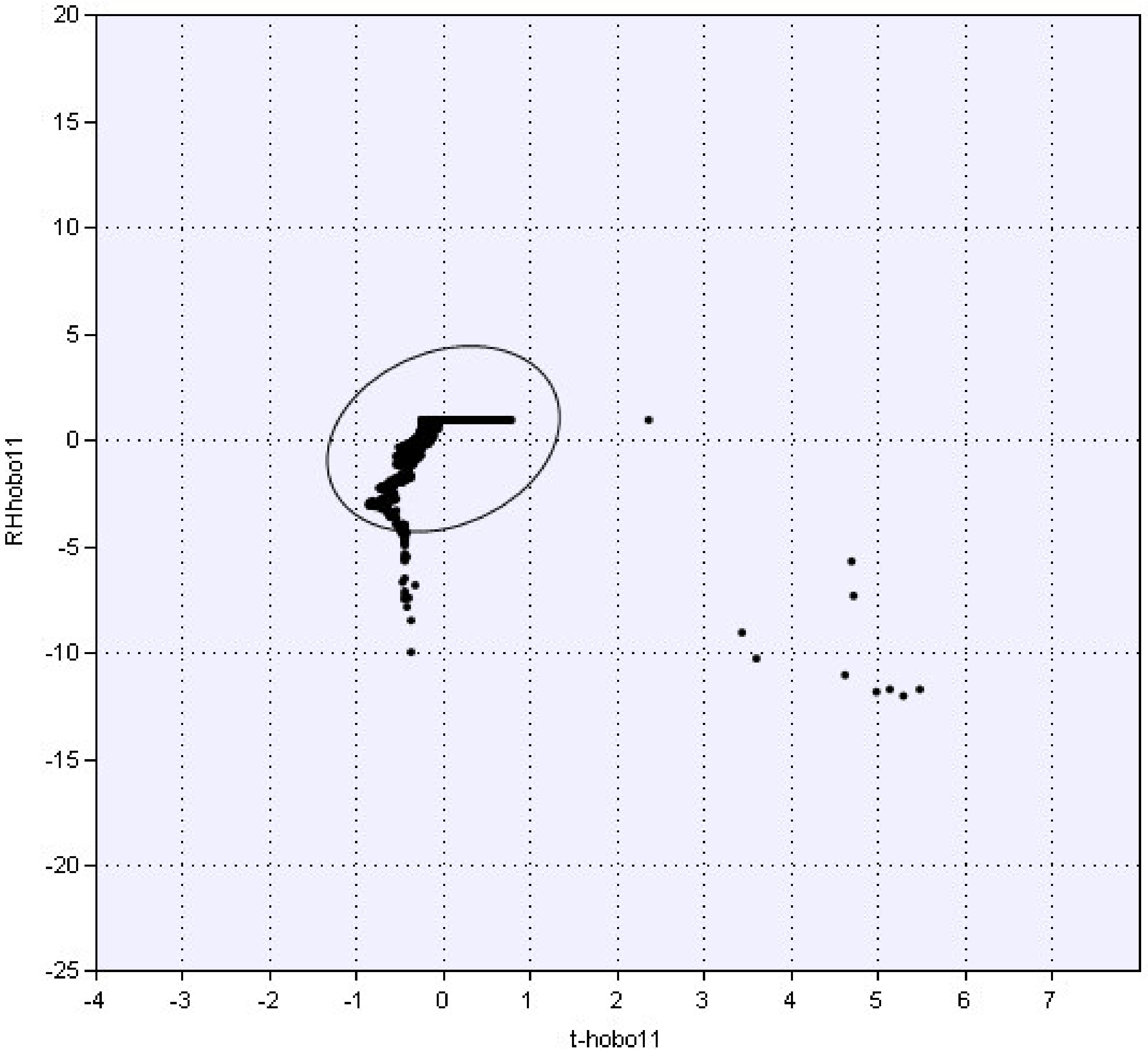

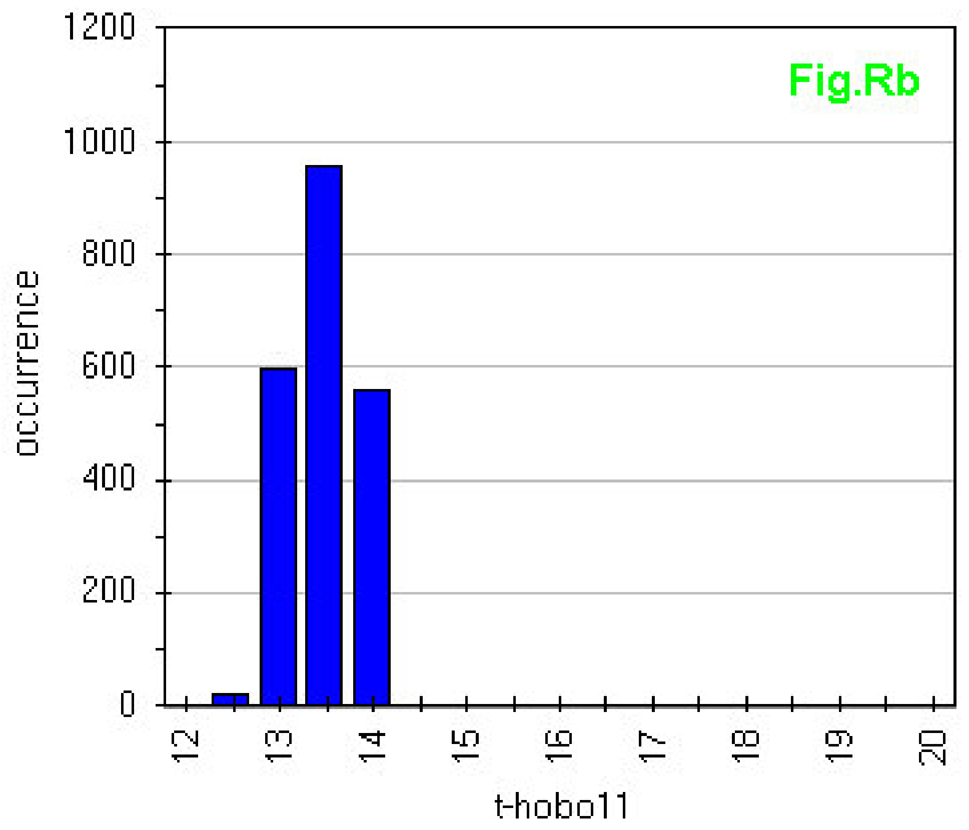

Fig.Rb
1200
1000
800
600
400
200
0
occurrence
12
13
14
15
16
17
18
19
20
t-hobo11

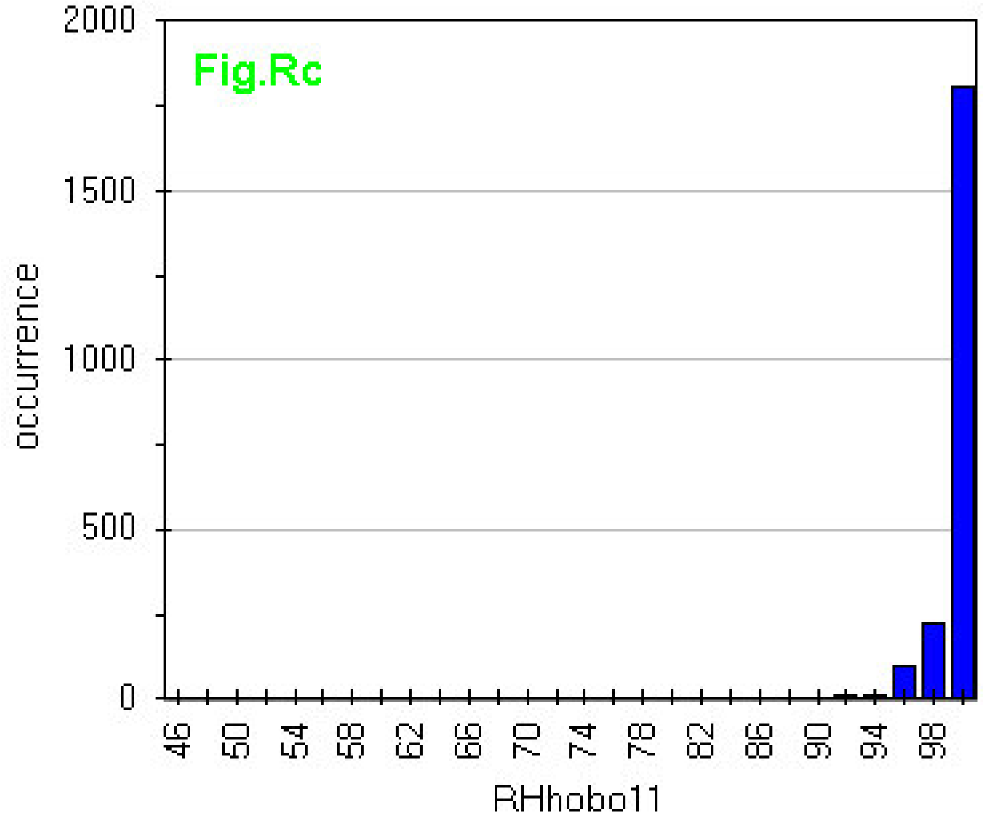
Fig.Rc
2000
1500
1000
500
0
occurrence
46
50
54
58
62
66
70
74
78
82
86
90
94
98
RHhobo11

Fig.Sa

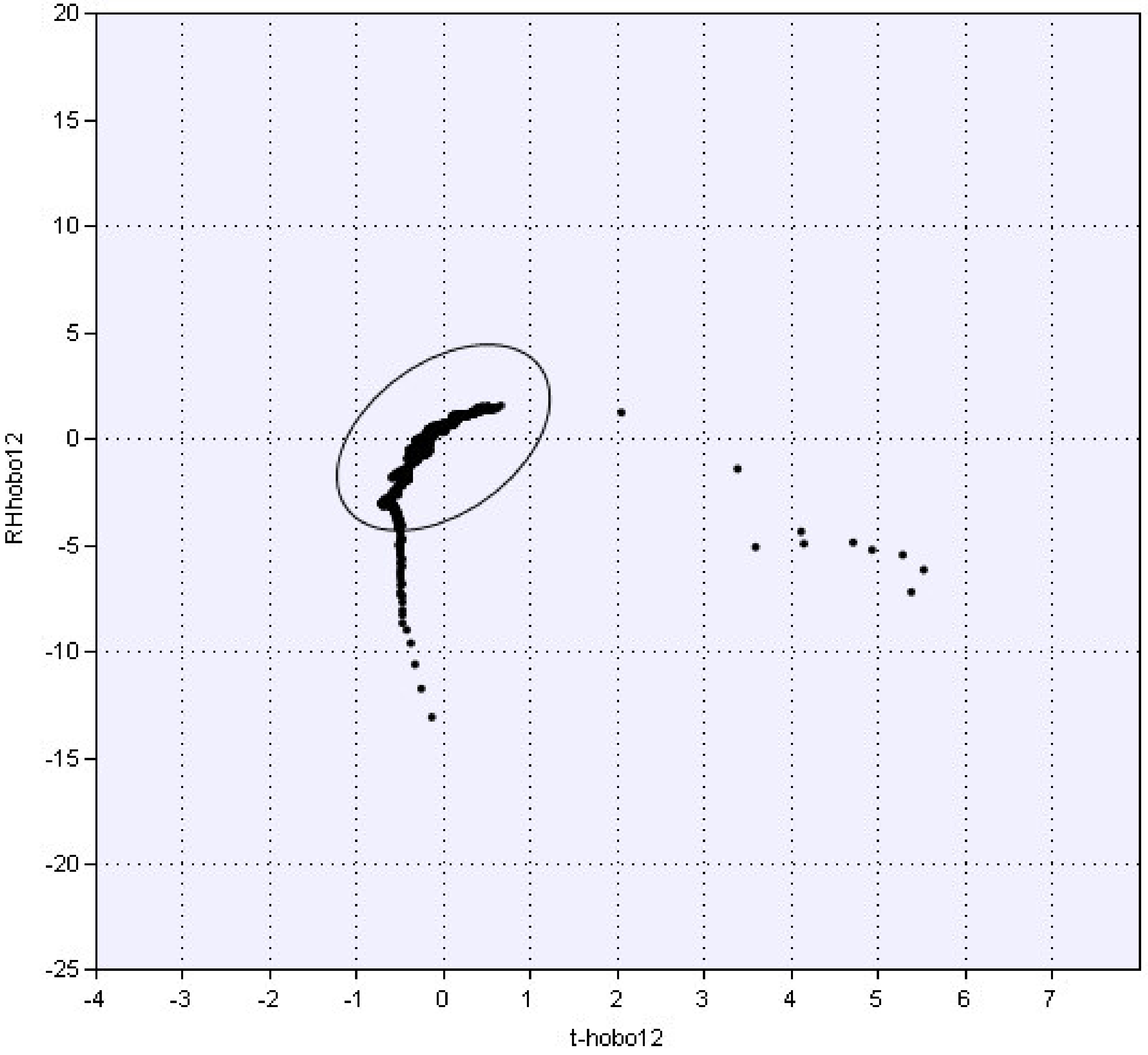

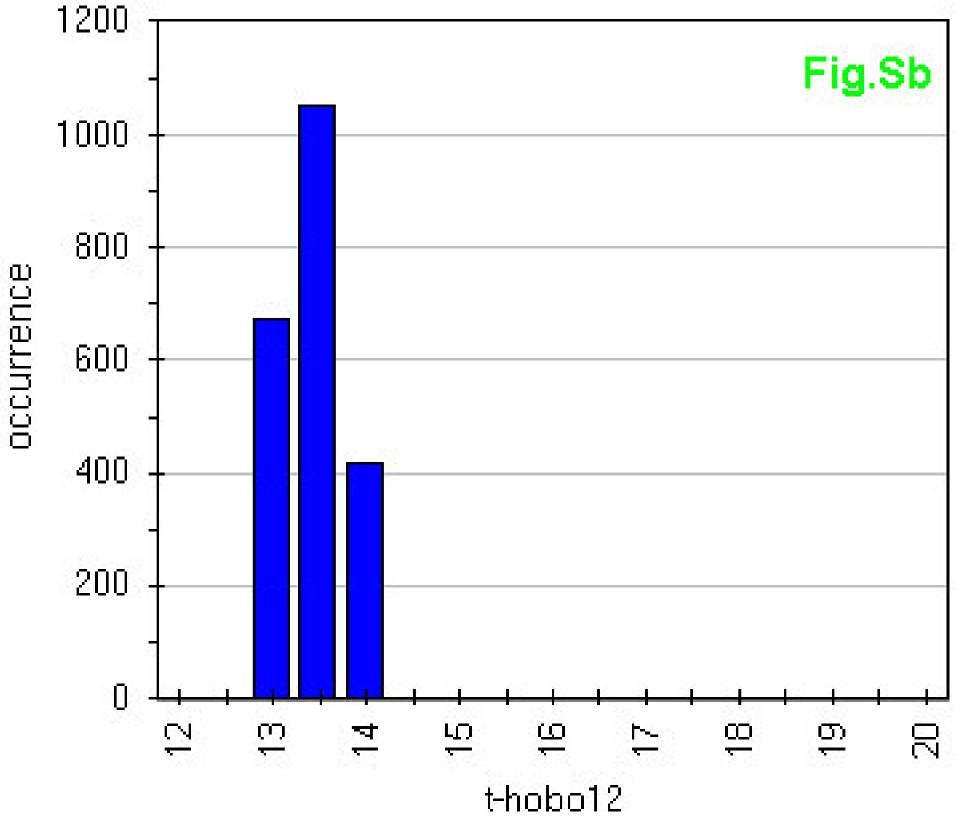
Fig.Sb
occurrence
1200
1000
800
600
400
200
0
12
13
14
15
16
17
18
19
20
t-hobo12

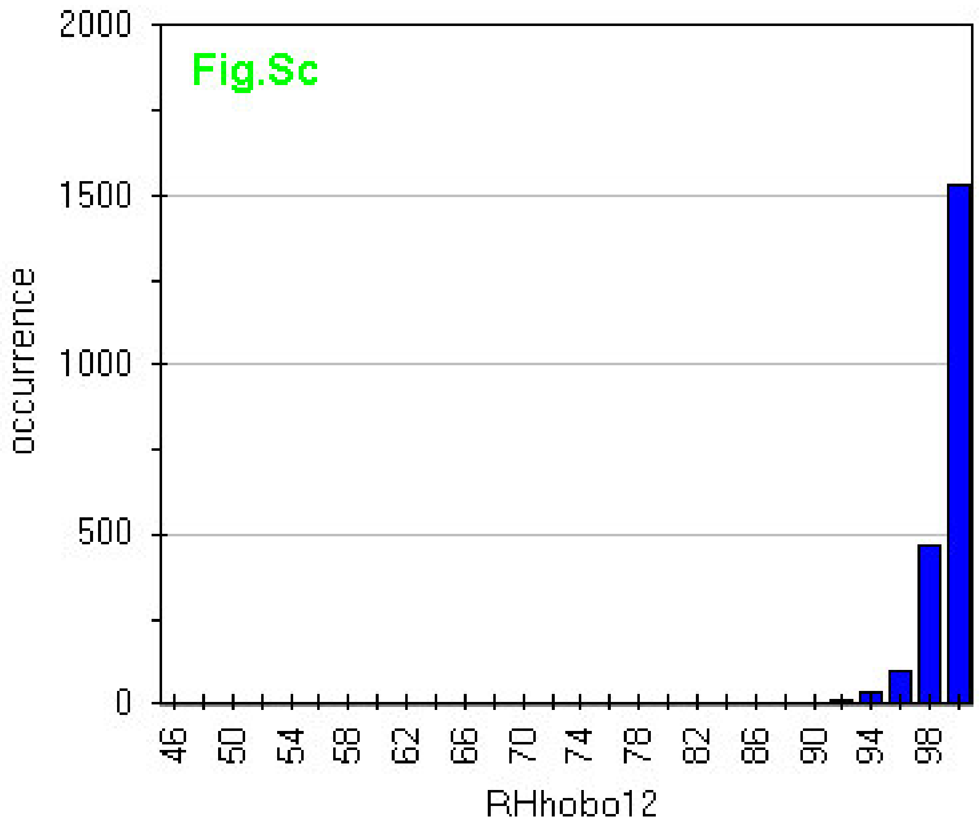

Fig.Sc
2000
1500
1000
500
0
occurrence
46
50
54
58
62
66
70
74
78
82
86
90
94
98
RHhobo12

Fig.Ta

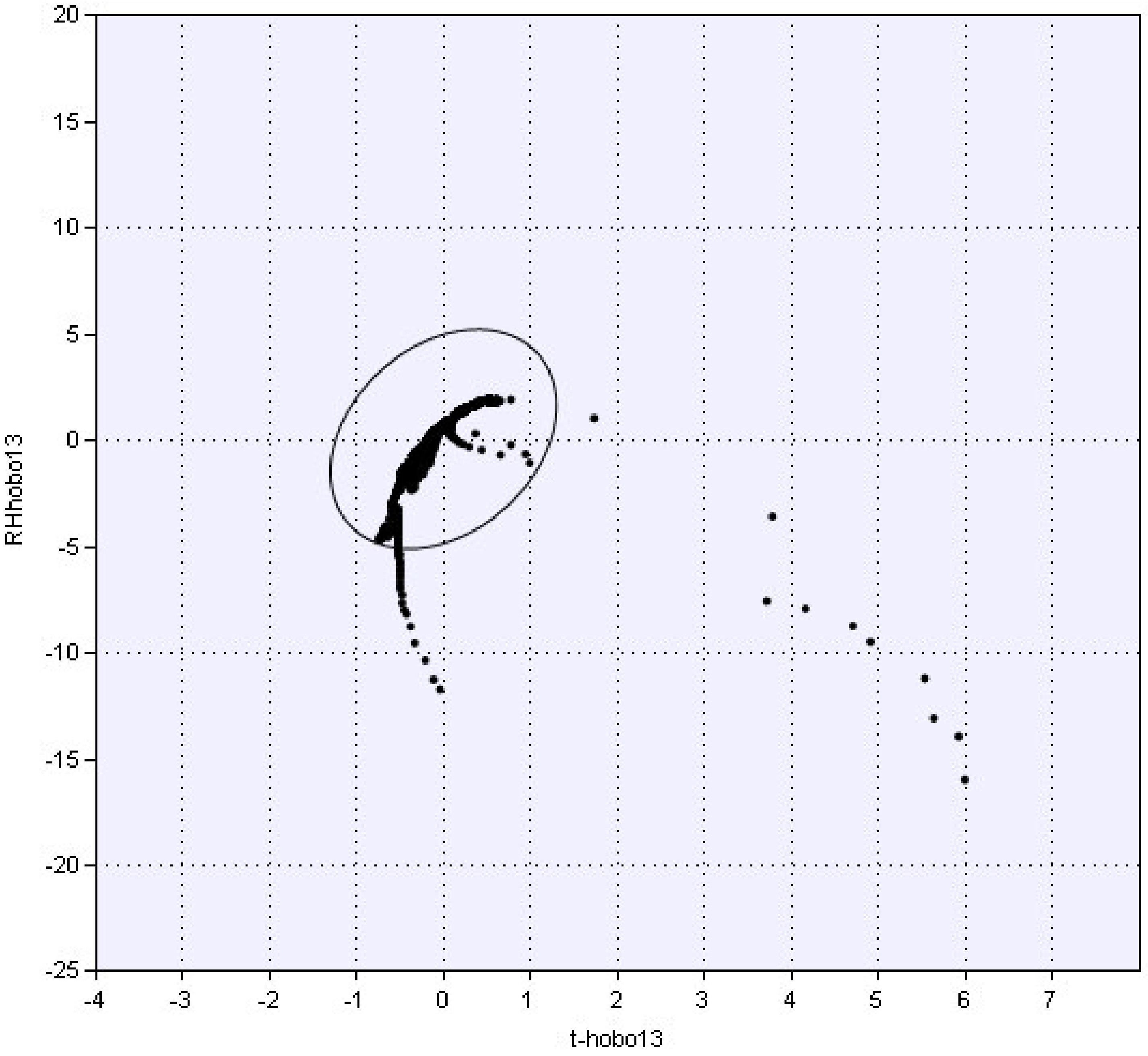

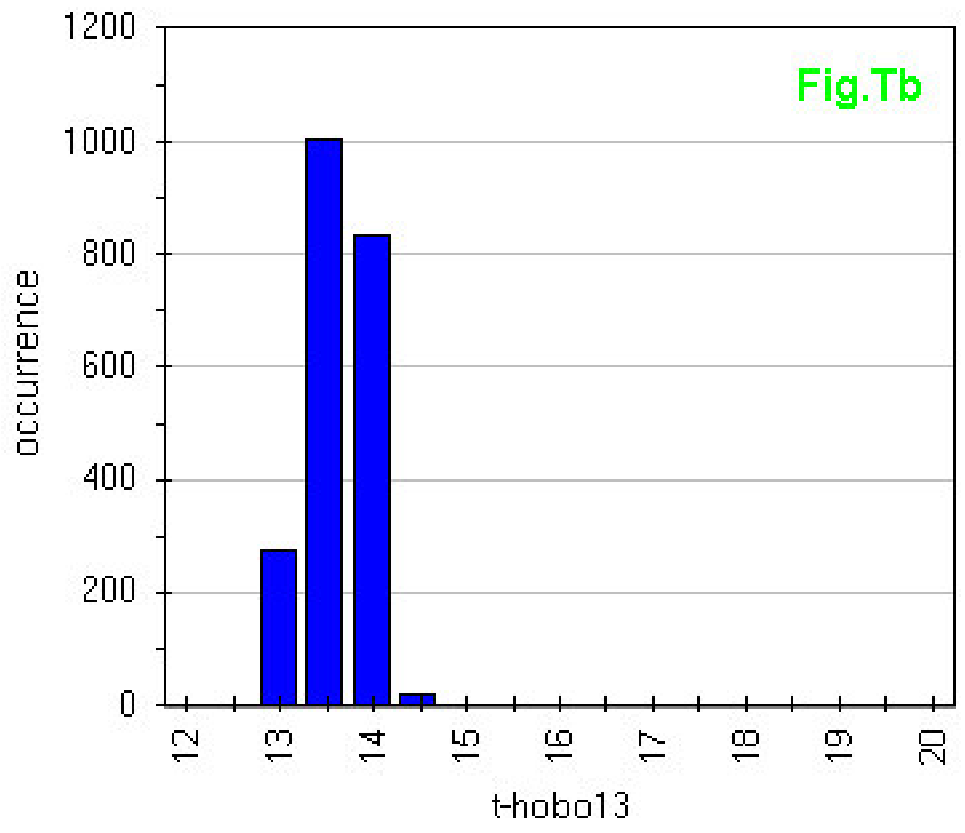

Fig.Tb
occurrence
1200
1000
800
600
400
200
0
12
13
14
15
16
17
18
19
20
t-hobo13

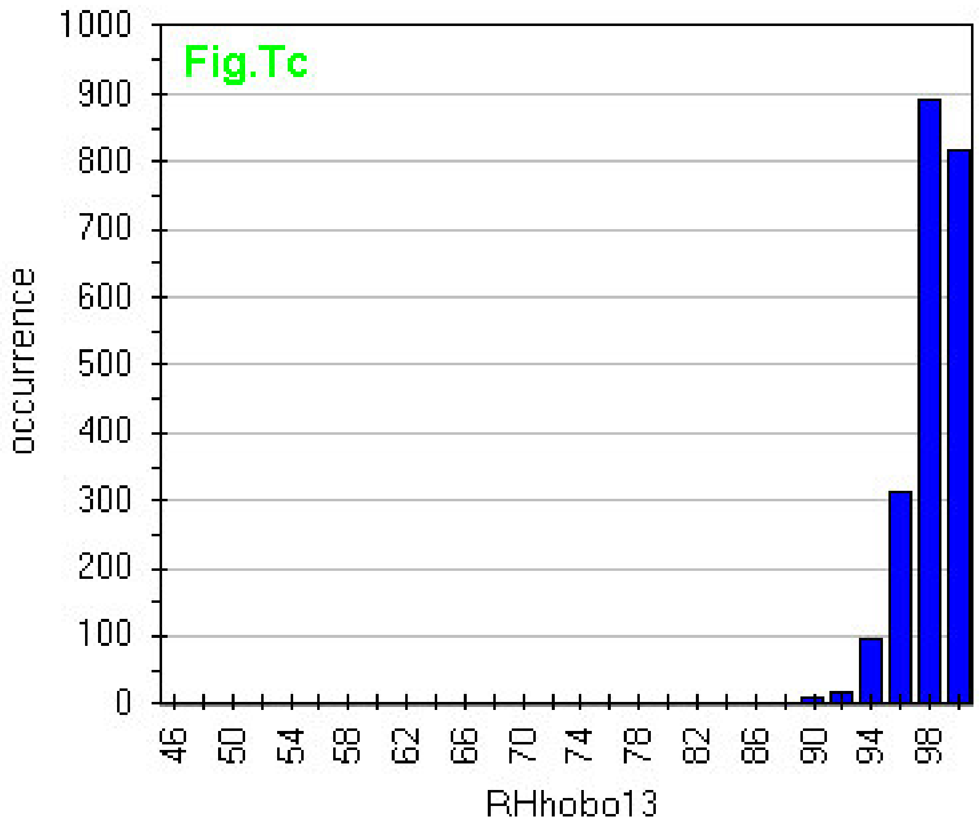
Fig.Tc
occurrence
1000
900
800
700
600
500
400
300
200
100
0
46
50
54
58
62
66
70
74
78
82
86
90
94
98
RHhobo13

Fig.Ua

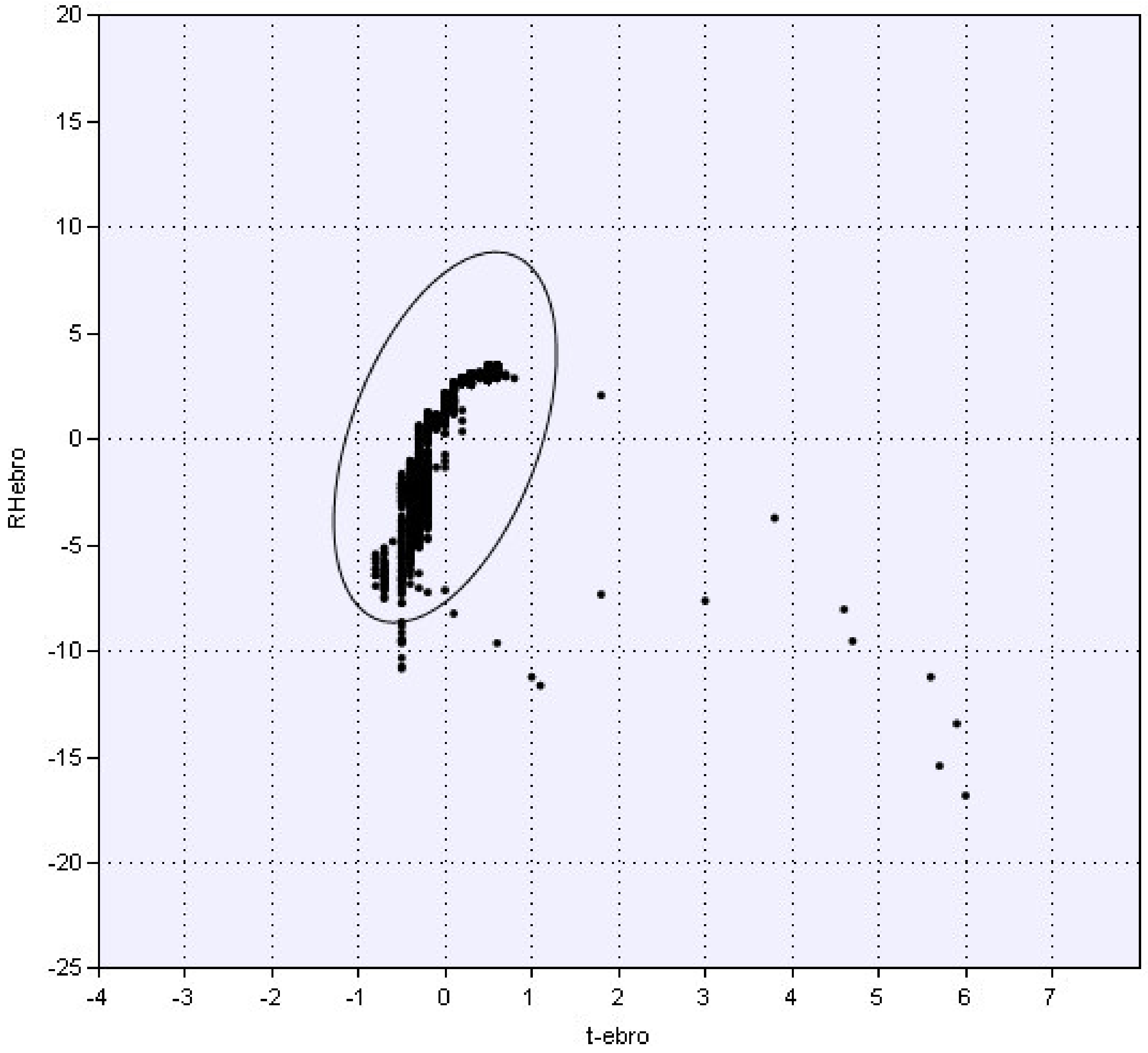

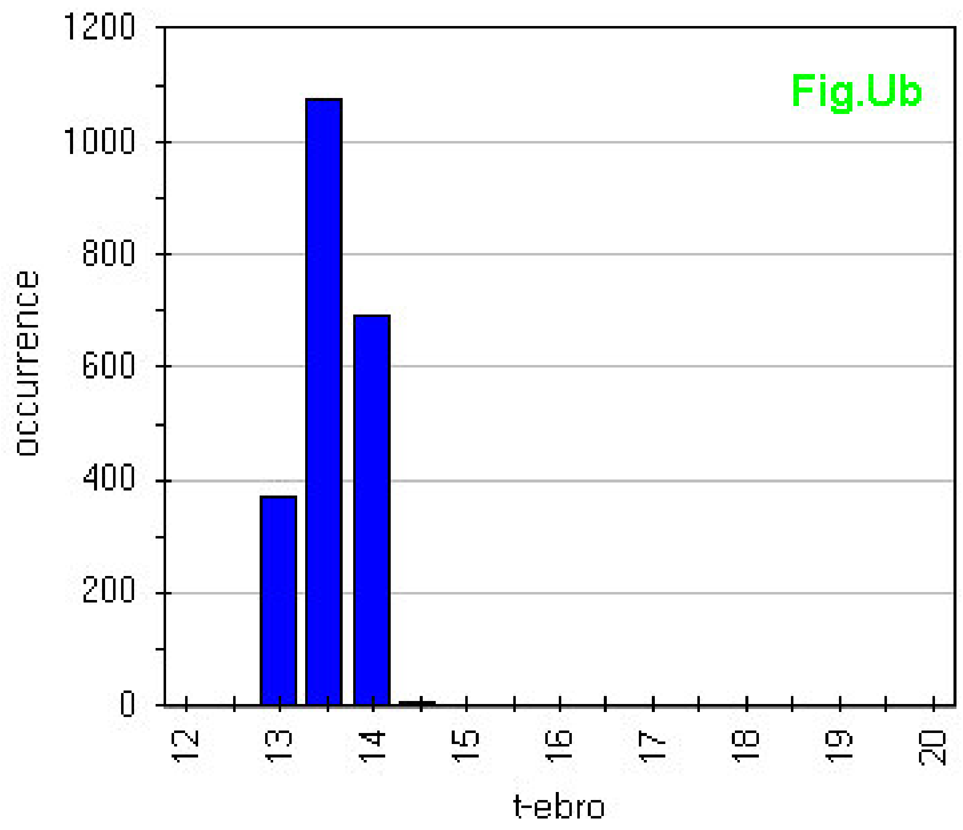
Fig.Ub
occurrence
1200
1000
800
600
400
200
0
12
13
14
15
16
17
18
19
20
t-ebro

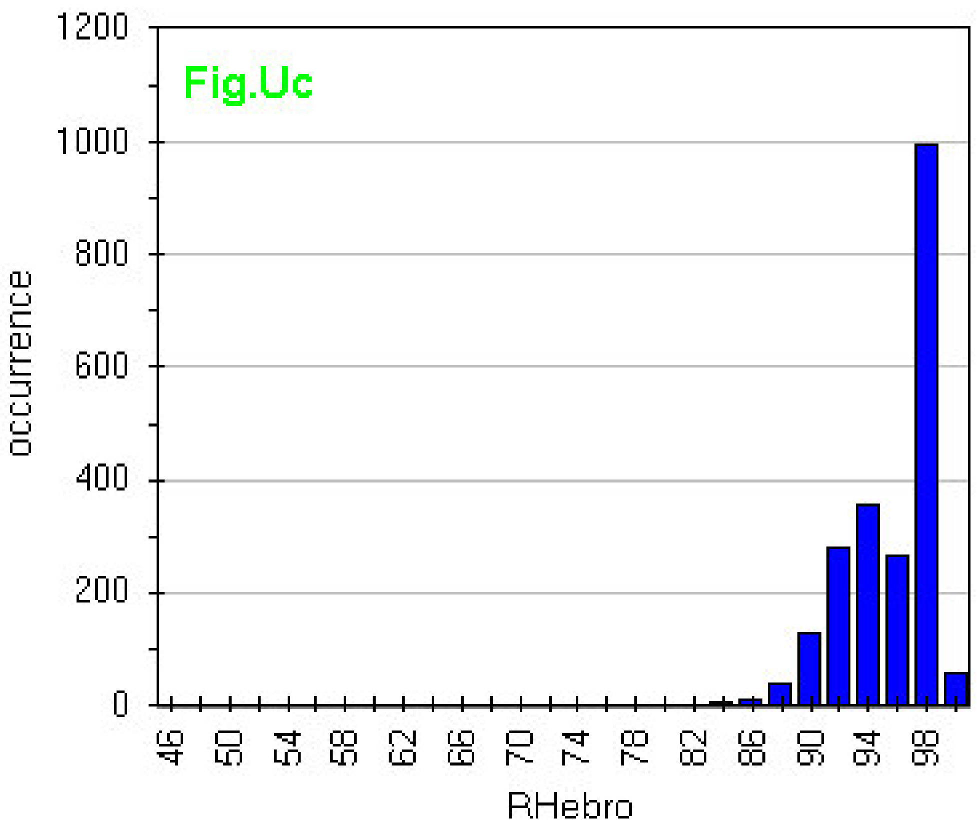
Fig.Uc
1200
1000
800
600
400
200
0
occurrence
46
50
54
58
62
66
70
74
78
82
86
90
94
98
RHebro

Fig.Va

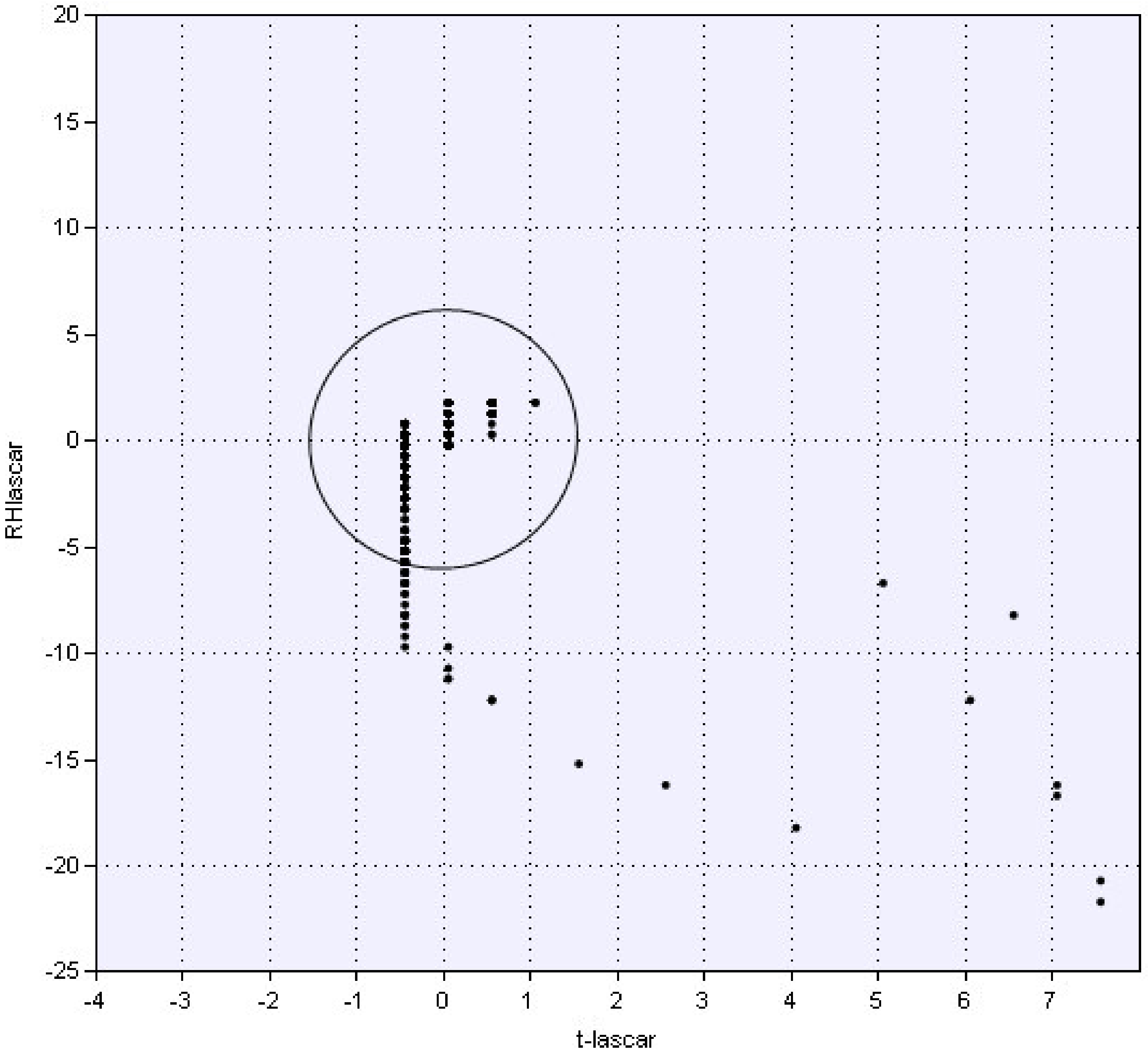

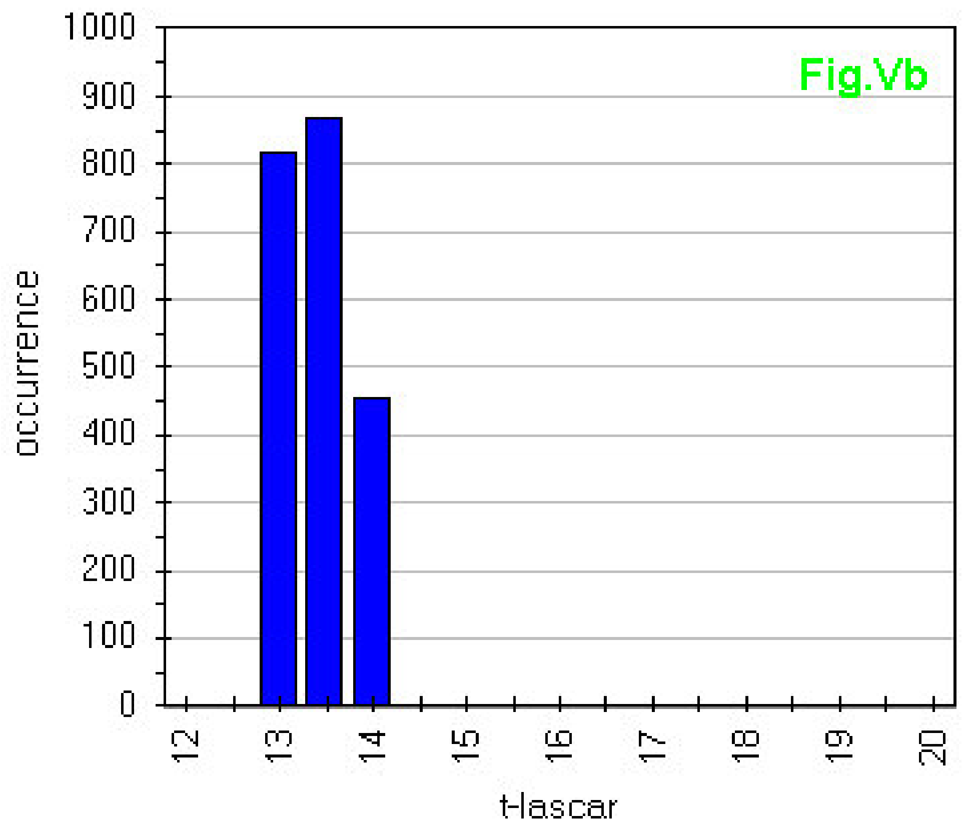

Fig.Vb
1000
900
800
700
600
500
400
300
200
100
0
occurrence
12
13
14
15
16
17
18
19
20
t-Tascar

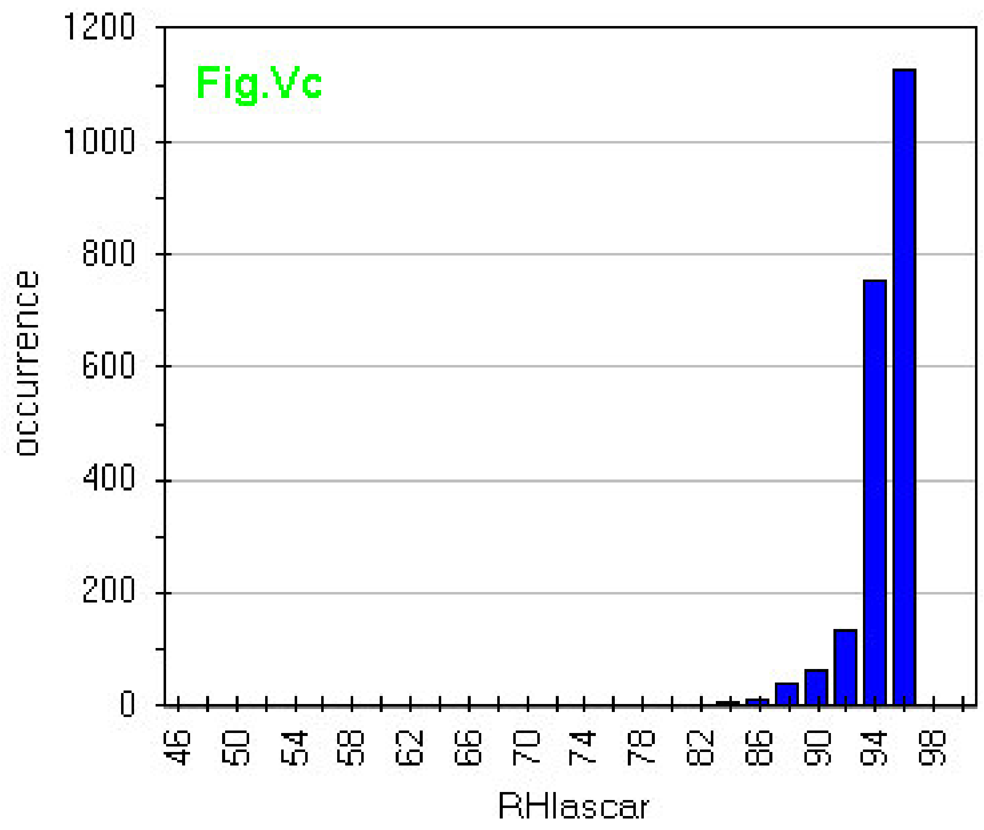

Fig.Vc
occurrence
1200
1000
800
600
400
200
0
46
50
54
58
62
66
70
74
78
82
86
90
94
98
RHlascar

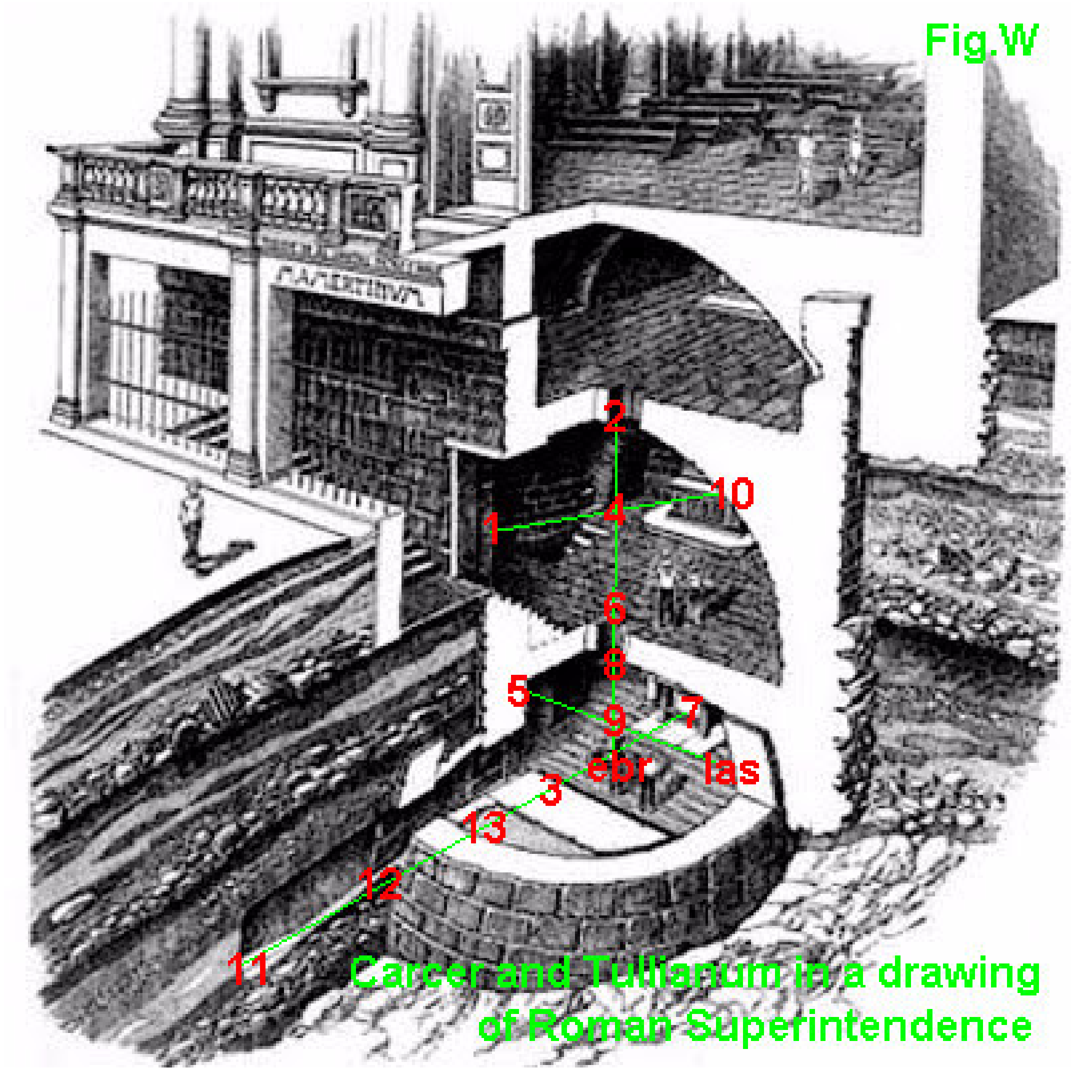

Fig.W

Carcer and Tullianum in a drawing of Roman Superintendence
